# The Combination of Untargeted Metabolomics and Machine Learning Predicts the Biosynthesis of Phenolic Compounds in *Bryophyllum* Medicinal Plants (Genus *Kalanchoe*)

**DOI:** 10.3390/plants10112430

**Published:** 2021-11-10

**Authors:** Pascual García-Pérez, Leilei Zhang, Begoña Miras-Moreno, Eva Lozano-Milo, Mariana Landin, Luigi Lucini, Pedro P. Gallego

**Affiliations:** 1 Agrobiotech for Health Group, Plant Biology and Soil Science Department, Biology Faculty, University of Vigo, E-36310 Vigo, Spain; pasgarcia@uvigo.es (P.G.-P.); elozano@alumnos.uvigo.es (E.L.-M.); pgallego@uvigo.es (P.P.G.); 2 CITACA—Agri-Food Research and Transfer Cluster, University of Vigo, E-32004 Ourense, Spain; 3 Department for Sustainable Food Process, Università Cattolica del Sacro Cuore, Via Emilia Parmense 84, 29122 Piacenza, Italy; leilei.zhang@unicatt.it (L.Z.); mariabegona.mirasmoreno@unicatt.it (B.M.-M.); 4 I+D Farma Group (GI-1645), Department of Pharmacology, Pharmacy and Pharmaceutical Technology, Faculty of Pharmacy, Universidade de Santiago de Compostela, E-15782 Santiago de Compostela, Spain; m.landin@usc.es; 5 Health Research Institute of Santiago de Compostela (IDIS), E-15706 Santiago de Compostela, Spain

**Keywords:** *Kalanchoe*, plant tissue culture, bioactive compounds, artificial intelligence, plant biotechnology, mineral nutrition, phytochemistry, polyphenols, secondary metabolism

## Abstract

Phenolic compounds constitute an important family of natural bioactive compounds responsible for the medicinal properties attributed to *Bryophyllum* plants (genus *Kalanchoe*, Crassulaceae), but their production by these medicinal plants has not been characterized to date. In this work, a combinatorial approach including plant tissue culture, untargeted metabolomics, and machine learning is proposed to unravel the critical factors behind the biosynthesis of phenolic compounds in these species. The untargeted metabolomics revealed 485 annotated compounds that were produced by three *Bryophyllum* species cultured in vitro in a genotype and organ-dependent manner. Neurofuzzy logic (NFL) predictive models assessed the significant influence of genotypes and organs and identified the key nutrients from culture media formulations involved in phenolic compound biosynthesis. Sulfate played a critical role in tyrosol and lignan biosynthesis, copper in phenolic acid biosynthesis, calcium in stilbene biosynthesis, and magnesium in flavanol biosynthesis. Flavonol and anthocyanin biosynthesis was not significantly affected by mineral components. As a result, a predictive biosynthetic model for all the *Bryophyllum* genotypes was proposed. The combination of untargeted metabolomics with machine learning provided a robust approach to achieve the phytochemical characterization of the previously unexplored species belonging to the *Bryophyllum* subgenus, facilitating their biotechnological exploitation as a promising source of bioactive compounds.

## 1. Introduction

*Bryophyllum* constitutes a subgenus within the *Kalanchoe* genus (Crassulaceae family) that contains several plant species commonly known as “*Bryophyllum*” in Ethnomedicine [1,2]. Accordingly, different *Bryophyllum*-derived formulations have been traditionally used worldwide for the treatment of diabetes, cardiovascular, and neoplastic diseases [3,4]. *Bryophyllum* spp. medicinal properties are a consequence of the production of phenolic compounds as recently established [4,5,6]. Thus, different extracts from *Bryophyllum* have been reported to exhibit valuable health-promoting properties because of the high contents of phenolic compounds, as determined through different in vitro assays. For instance, antioxidant activity was assessed in terms of free radical scavenging activity, inhibition of lipid peroxidation, and prevention of oxidative hemolysis [6,7], together with anti-inflammatory activity, antimicrobial activity towards a wide range of both bacterial and fungal strains, and anticancer activity. Indeed, the anticancer activity of *Bryophyllum* was determined towards a wide panel of cancer cell lines, such as MCF-7 breast adenocarcinoma, NCI-H460 non-small cell lung carcinoma, HeLa cervical carcinoma, and HepG2 hepatocellular carcinoma cell lines [6]. Consequently, due to the aforementioned health properties associated with *Bryophyllum* extracts, novel strategies should be proposed to ensure the large-scale production of phenolic compounds from these underexplored medicinal plants.

Nevertheless, little information is available on the biosynthesis of phenolic compounds in *Bryophyllum* plants, making difficult their exploitation as a valuable source of bioactive compounds. Concerning the biosynthetic pathway of phenolic compounds, in brief, the major precursor is cinnamic acid, synthesized after the action of phenylalanine ammonia-lyase on the amino acid phenylalanine [8]. Afterwards, cinnamic acid may either be incorporated into the biosynthesis of phenolic acids (C6-C1 and C6-C3 compounds) or undergo an enzymatic transformation to produce coumaroyl-CoA that is assumed to be the common basic structure for the biosynthesis of other subfamilies, namely: lignans ((C6-C3)2), stilbenes (C6-C2-C6), and flavonoids (C6-C3-C6) [9]. Finally, these subfamilies are later subjected to condensation to give rise to polymeric phenolic compounds, as is the case for lignin and tannins [9]. In particular, phenolic acids (protocatechuic acid, caffeic acid, and ferulic acid), flavonoids (myricetin, quercetin, and kaempferol glycosides), and anthocyanins (malvidin glycosides) have been identified as the main phenolic compounds in *Bryophyllum* plants [5,6,10]. In this sense, to gain insight into the biosynthesis of phenolic compounds, the application of untargeted metabolomics (UM) becomes an essential high-throughput approach: it confers a fast, reliable, and detailed perspective of the metabolite pool found in plants, thus contributing to the identification of their wide array of compounds present in cells and tissues [11,12] but also facilitating the rapid industrial exploitation of those promising bioactive compounds.

Additionally, the establishment of plant tissue culture (PTC) has emerged as a solid methodology to characterize phenolic compound biosynthesis. PTC constitutes a reliable and controlled biotechnological system able to achieve homogeneous and standard bioactive compound production [13,14,15]. For this purpose, the design of optimized culture media formulations is crucial. Its development must be carefully carried out to achieve a correct balance between bioactive compounds accumulation and the preservation of tissue culture integrity [15,16]. Additional factors, such as the genotype or the type of explant, also play important roles in the biosynthesis of phenolic compounds [17,18]. Thus, the great number of factors affecting this process limits the interpretation of results and the achievement of general conclusions [19,20,21].

In the last decade, the application of machine learning (ML) technology has been replacing the traditional statistical methods to easily reveal exhaustive information about multivariate processes in which occluded patterns and complex interactions occur [22,23,24]. Among the different ML algorithms available, the combination of artificial neural networks (ANNs) with fuzzy logic (neurofuzzy logic, NFL) has already been successfully applied in the field of PTC for the characterization and optimization of diverse multifactorial processes, including seed germination [25], micropropagation [26], and the identification of physiological disorders [27]. The application of ANNs provides the establishment of predictive mathematical models obtained after training the empirical data, including the independent variables or factors as inputs and the dependent variables or parameters as outputs, to predict the key factors involved in each parameter, as well as their potential interactions [28]. To enhance the interpretation of the resulting ANN model, the application of fuzzy logic facilitates this task by the formulation of ‘IF–THEN’ rules, which confers an understandable linguistic definition of the model results [29]. In this way, NFL contributes to the characterization and understanding of complex processes and, simultaneously, it may be regarded as a useful decision-making tool for optimization, as it has been previously used for maximizing the production of phenolic compounds [18].

In this work, a combinatorial approach including UM and ML is proposed in order to decipher the critical factors affecting the biosynthesis of phenolic compounds in three *Bryophyllum* species cultured in vitro. We hypothesize that the combination of both cutting-edge methodologies will assist in the phytochemical valorization of these unexplored medicinal plants and will confer a novel approach to contribute to their biotechnological exploitation in different sectors, including the food, cosmeceutical, and pharmaceutical industries. Furthermore, due to the vast information provided by both UM and ML, and thanks to their plasticity, their combination will be priceless to increase the knowledge of novel sources of bioactive compounds with beneficial properties on human health, from unexplored plant sources, thus conferring a multidisciplinary workflow regarding the large-scale production of those phytochemicals.

## 2. Results

### 2.1. Determination of Phenolic Profiling by Untargeted Metabolomics

The phenolic profiling of *Bryophyllum* plants was determined by using ultra-high-pressure liquid chromatography coupled to a quadrupole-time-of-flight mass spectrometer (UHPLC-QTOF/MS), considering three species—i.e., *Bryophyllum daigremontianum* (BD), *Bryophyllum × houghtonii* (BH), and *Bryophyllum tubiflorum* (BT), two organ parts—i.e., aerial parts and roots, and seven culture media formulations—i.e., full-strength medium (MS), half-strength medium (1/2MS), quarter-strength medium (1/4MS), and eighth-strength medium (1/8MS) of both macronutrients (M) and micronutrients (µ).

The resulting profile showed a total of 485 putatively annotated compounds. The full list of annotated compounds is provided accompanied by their retention time and composite mass spectrum (Table S1). Flavonoids were the most abundant subfamily of phenolic compounds, mainly characterized by anthocyanins, flavonols, and flavones, followed by phenolic acids, low-molecular-weight phenolics, and other subfamilies. Among the flavonoids, malvidin, and pelargonidin presenting 3-O- or 3,5-O- glycoside bonds were the most abundant anthocyanins, followed by myricetin 3-O-, kaempferol 3-O-, and quercetin 3-O- glycosides as the most representative members of the flavonol subfamily, and apigenin 7-O-, apigenin 6,8-C-, luteolin 6-O-, and luteolin 6-C belonging to the flavone subfamily (Table S1). Concerning the phenolic acid subfamily, hydroxybenzoic acids and hydroxycinnamic acids were the most prevalent compounds, with protocatechuic acid 4-O-glucoside, caffeoylquinic acid mono- and di- glycoside, and cinnamic acid as the most abundant ones. In addition, a significant number of low-molecular-weight phenolics, i.e., alkylphenols, hydroxybenzaldehydes, hydroxycoumarins, hydroxyphenylpropenes, tyrosols, and other simple phenylpropanoids, were also detected (Table S1).

**Figure 1:**
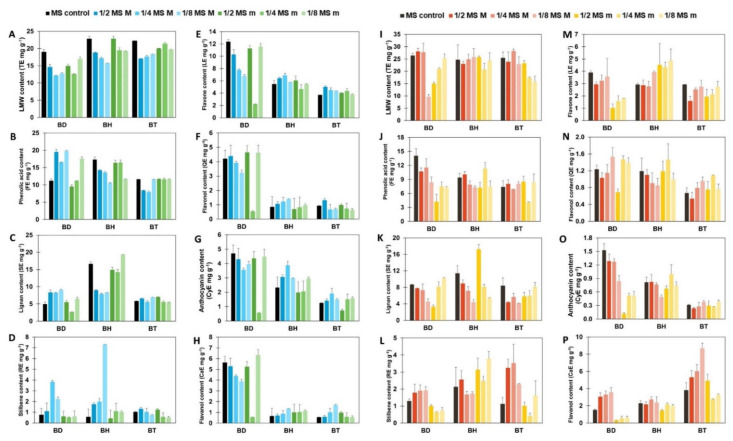
Semi-quantification of each subfamily of phenolic compounds. All results are expressed in mg g^−1^ of equivalents for each reference compound. Vertical bars indicate the standard deviation. (**A**–**H**): phenolic content of aerial parts. (**I**–**P**): phenolic content of roots. TE, tyrosol equivalents, for the LMW content; FE, ferulic acid equivalents, for the phenolic acid content; SE, sesamin equivalents, for the lignan content; RE, resveratrol equivalents, for the stilbene content; LE, luteolin equivalents for the flavone content; QE, quercetin equivalents, for the flavonol content; CyE, cyanidin equivalents, for the anthocyanin content; CaE, catechin equivalents, for the flavanol equivalents.

**Figure 2:**
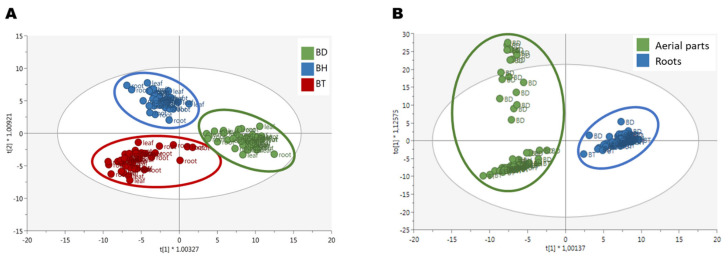
OPLS-DA score plots for the discriminant phenolic profile of (**A**) *Bryophyllum* species and (**B**) plant organs.

Afterward, a semi-quantification of phenolic compounds was performed, using reference compounds for each subfamily. The results are shown in Figure 1, for both aerial parts (Figure 1A–H) and roots (Figure 1I–P). As it can be seen by the statistical analysis performed by factorial analysis of variance (ANOVA), all the factors tested: genotypes, plant organs, and culture media composition, influenced the accumulation of phenolic compounds, as well as their potential interactions (*p* < 0.001; Table S2). Due to a large number of factors and data collected, the information provided by such analysis for the identification of simple patterns and/or identification of interactions between variables was limited. Consequently, a multivariate statistical approach was carried out to determine the influence of the genotypes and the organs on the biosynthesis of phenolic compounds. An Orthogonal Projection in Latent Structures—Discriminant Analysis (OPLS-DA) was used, revealing the discriminant compounds between species (BD, BH, and BT) and their organs (aerial parts and roots) (Figure 2). Additionally, the Variable Importance in Projection (VIP) selection method was used to identify the VIP markers implicated in the discrimination between genotypes (Table S3) and plant organs (Table S4).

**Figure 3:**
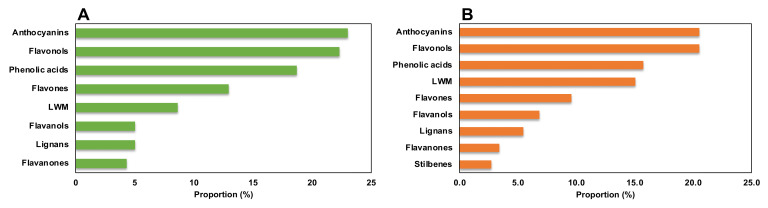
Proportion of compounds predicted as discriminant for the phenolic profile among (**A**) *Bryophyllum* species and (**B**) plant organs.

The three different genotypes, BD, BH, and BT, presented a differential composition in terms of phenolic compound production (Figure 2A), as assessed by the high quality of the generated model in terms of linearity and predictability (R^2^Y = 0.907 and Q^2^Y = 0.856, respectively). Regarding the compounds with the highest contribution to the discrimination between genotypes, anthocyanins, flavonols, and phenolic acids were the most prevalent subfamilies (Figure 3A), accounting for > 60% of total discriminant compounds. Anthocyanins were mainly represented by cyanidin, malvidin, and pelargonidin glycosides; flavonols were mainly represented by quercetin, kaempferol, and myricetin glycosides; and phenolic acids showed a great heterogeneity, but caffeic, ferulic, and gallic acid derivatives had a higher prevalence (Table S3). Thus, such compounds are predicted to be present in a genotype-dependent manner.

Regarding the discrimination of the phenolic profiles of plant organs (Figure 2B), there was a clear differential pattern between aerial parts and roots, as reflected by the OPLS-DA model with linearity and predictability parameters of high quality (R^2^Y = 0.960 and Q^2^Y = 0.926, respectively). Similar to the genotype-based discrimination, anthocyanins, flavonols, and phenolic acids were predicted as the most relevant subfamilies contributing to such differences, together with low-molecular-weight phenolics (LMW), which included more than 70% of total annotated compounds (Figure 3B). In this case, anthocyanins were mainly represented by malvidin and cyanidin glycosides, flavonols were represented by quercetin, kaempferol, and myricetin glycosides, phenolic acids were represented by cinnamic acids derivatives, and LMW compounds were mainly represented by catechols and coumarins (Table S4). Accordingly, these compounds are suggested to present an organ-restricted distribution in *Bryophyllum*.

### 2.2. Machine Learning Prediction of the Biosynthesis of Phenolic Compounds

Once the influence of genotypes and organs on the biosynthesis of phenolic compounds was assessed, the composition of culture media formulations was the remaining factor whose influence had to be determined, as well as the interactions between them. For that purpose, ML modeling was carried out to identify the critical factors affecting the biosynthesis of phenolic compounds in *Bryophyllum* and deciphering the potential multivariate interactions that may occur. The NFL results are shown in Table 1. The model efficiently predicted seven out of the eight outputs evaluated, showing a high predictability with training set R^2^ values > 70% (Table 1). MSE values also assessed the model quality, together with the ANOVA performed, which showed no statistical differences between the experimental and predicted values (F ratio > *f* critical; Table 1). Only one output could not be predicted by the model: the flavone production (training set R^2^ < 70%), probably due to the heterogeneous composition of this subfamily employed for its semi-quantification, which included flavones, flavanones, and related compounds. To interpret how each output was affected by the predicted inputs, the model was accompanied by the generation of ‘IF–THEN’ rules together with their membership degree, which are shown in Table 2. The ranked values provided for the inputs are displayed in Figure S1.

LMW biosynthesis was mainly predicted as a function of the interaction organ × sulfate (Table 1). However, additional submodels indicated that the interaction genotype × copper and phosphate concentration played a secondary role in LMW biosynthesis. In fact, LMW concentration was the output with the highest number of submodels, which can be explained by the great heterogeneity of compounds that make up this subfamily: tyrosols, coumarins, and catechols (Table S1). Due to their different biosynthetic origins, it is reasonable to find many factors causing the production of LMW compounds. According to the model, the interaction of roots with high sulfate concentrations (>1.43 mM) caused a high LMW content with the highest membership degree (Table 2; rule 8). Generally, a high LMW content was observed in both aerial parts and roots under mid- to high sulfate concentrations (0.94 mM; Table 2; rules 3–4, 7–8). In contrast, the combination of aerial parts and low sulfate concentrations (<0.61 mM) caused a low LMW content with the highest membership degree (Table 2; rule 1). In the case of copper, high LMW content values were obtained by low concentrations in the case of BD and BT (<0.03 mM; Table 2; rules 9 and 15, respectively) and low and mid concentrations in the case of BH (<0.08 mM; Table 2, rules 12–13). In the same way, high LMW contents were caused by low phosphate concentrations (<0.71 mM), thus suggesting an inhibitory role (Table 2; rule 18).

Phenolic acid biosynthesis was mainly predicted by the interaction genotype × copper and, secondarily, by the organ (Table 1). The rules for phenolic acid content indicated that high values were primarily due to BD and high copper concentrations (> 0.08 µM; Table 2; rule 24) and, to a lower extent, aerial parts (Table 2; rule 20). In contrast, a low phenolic acid content was determined for the rest of the conditions, being the combination of BD with moderate copper concentrations (0.03–0.08 µM), the condition showing the low value with the highest membership degree (Table 2; rule 23).

In the case of lignans, only one model was generated, predicted by the interaction genotype × sulfate × organ (Table 1). In this way, high levels were only observed in the case of BH combined with high sulfate concentrations (>1.11 mM; Table 2; rules 37 and 38), but aerial parts presented the highest membership degree (0.84; Table 2; rule 37). On the contrary, a low lignan content was observed for the rest of the conditions, being the combination of BT, roots, and low sulfate concentrations (<1.11 mM) that showed the highest membership degree (0.90; Table 2; rule 40).

Stilbene biosynthesis showed the most complex prediction since it was mainly predicted by the combination of calcium × organ × genotype and, secondarily, by the interaction genotype × phosphate × organ (Table 1). Thus, a high stilbene content was predominantly caused by the combination of BH, aerial parts, and low calcium concentrations (<1.68 mM; Table 2; rule 44), whereas a low stilbene content was obtained also by the combination of BH and aerial parts but with a high calcium concentration (>1.68 mM; Table 2; rule 50). In the second submodel, high stilbene concentrations were caused by low phosphate concentrations (<0.43 mM) in BD and BT (Table 2; rules 55–56 and 67–68, respectively), but high phosphate concentrations (>0.98 mM) were required for BH to achieve high levels (Table 2; rules 65 and 66).

For flavonol and anthocyanin contents, only one model was generated in both cases, represented by the interaction genotype × organ, with the independence of culture media formulation (Table 1). In the case of flavonols, high values were only obtained in the case of aerial parts from BD (Table 2; rule 73). Under the rest of the conditions (Table 2; rules 74–78), low values were obtained; the interaction of BT with roots showed the highest membership degree (0.94; Table 2; rule 78). Similar results were obtained for the anthocyanin content, showing high values with the combination of aerial parts with BH and BD, the latter showing the highest contribution, according to their membership degree (0.57 and 0.78, respectively; Table 2; rules 79 and 80). Similar to flavonols, the low anthocyanin content was predominantly caused by the combination of roots from BT (Table 2; rule 84).

Finally, the flavanol content was mainly caused by the combination of genotype × organ and, secondarily by magnesium × organ (Table 1). BT was the most critical genotype associated with flavanol biosynthesis showing the highest contribution to the high flavanol content in roots (Table 2; rule 94) and a low content in aerial parts (Table 2; rule 93). Concerning the influence of magnesium, only high levels were observed under low magnesium concentrations (<0.85 mM) in roots (Table 2; rule 86).

### 2.3. Proposed Mechanism of Phenolic Compound Biosynthesis of Bryophyllum Plants Cultured In Vitro

**Figure 4:**
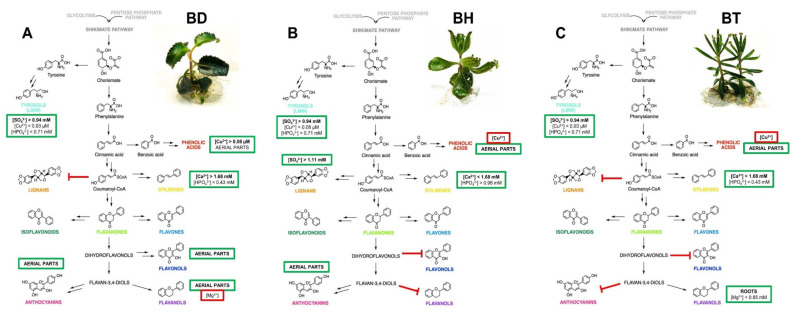
Proposed mechanism, as predicted by NFL modeling, for the biosynthesis of phenolic compounds in *Bryophyllum* cultured in vitro. (**A**) *B. daigremontianum*, BD; (**B**) *B.* × *houghtonii*, BH; (**C**) *B. tubiflorum*, BT. Green squares include the factors playing a positive role in the biosynthesis of each subfamily, whereas red squares include the factors with an inhibitory role. Bold letters indicate the most prevalent factor for each output, showing the highest contribution, according to the predictive model.

The wide variety of interactions predicted between all the factors involved in the biosynthesis of phenolic compounds in *Bryophyllum* makes the interpretation of the obtained results difficult. As a solution, the generation of the NFL model provided valuable knowledge on this complex process, which is in accordance with the previously performed by ANOVA and OPLS-DA models, since all the outputs predicted by NFL modeling showed a significant influence of genotype, organ, and culture media composition, thus conferring strong evidence: the biosynthesis of phenolic compounds in these plants followed a genotype- and organ-dependent behavior, which was affected by mineral nutrition. Due to the complexity associated with the great number of rules given by the NFL, a graphical representation better represents all of the integrative information obtained. Thus, a proposed biosynthetic pathway, reflecting all the factors involved in the production of phenolic compounds for each species included in this study, is shown in Figure 4.

Similar patterns are shown for the biosynthesis of LMW compounds in all three species, whereas a differential behavior was observed for the rest of the phenolic subfamilies (Figure 4). In the case of phenolic acids, they mostly accumulated in aerial parts, but copper played a pivotal role depending on the species, causing a positive effect on BD, whereas it was shown as an inhibitor on BH and BT. Concerning lignans and stilbenes, BH followed a differential pattern with respect to BD and BT, since lignans mainly accumulated in BH, whereas the mineral requirements for stilbene biosynthesis were contrary to those found for BD and BT. Flavonols mainly accumulated in the aerial parts of BD, together with anthocyanins, the latter being also present in the aerial parts of BH, whereas both subfamilies were present in low concentrations in BT. Finally, flavanols showed a characteristic pattern, since they mostly accumulated in the roots of BT, with magnesium playing a positive role, whereas in the case of BD, they accumulated in aerial parts and were inhibited by magnesium.

In consequence, the combination of UM with NFL emerged as a promising approach to characterize highly complex processes by providing exhaustive information that is easy to interpret. Our results clearly show that, although these three *Bryophyllum* species are closely related, a genotype and organ-dependent pattern was observed for the biosynthesis of phenolic compounds in *Bryophyllum* cultured in vitro, depending on the composition of culture media. Such results are the consequence of ML modeling of the experimental results (Figure 1), which displayed cryptic information that did not show clear patterns and, therefore, conferred scarce information, thus limiting the enormous potential offered by the phenolic profile obtained by the untargeted metabolomics approach.

## 3. Discussion

Phenolic compounds play an essential role in the therapeutic properties of *Bryophyllum* plants since they have been seen to be efficient antioxidant, cytotoxic, anti-inflammatory, and antimicrobial agents [6,30]. In recent years, these metabolites have attracted much attention from a biotechnological point of view due to their pleiotropic beneficial effects on human health [31,32]. The establishment of PTC constitutes a reliable biological platform for the perpetual production of industrially important bioactive compounds, largely exploited in the field of plant biotechnology [33,34]. PTC has been recently assessed as an efficient approach for achieving the phytochemical valorization of multiple *Bryophyllum* species, including *B. daigremontianum* (BD), *B. × houghtonii* (BH), and *B. tubiflorum* (BT) [6,35]. In fact, the establishment of PTC promoted the production of several phenolic compounds, such as anthocyanins, which have not been described in plants based on conventional breeding [6].

In this work, the above-mentioned species were selected because of their wide application in the traditional medicine for the treatment of several prevalent diseases, ranging from wound healing and cough alleviation to chronic diseases, such as diabetes, neoplastic, cardiovascular, and neurological diseases, among others [1,2]. Nonetheless, most investigations have been exclusively focused on the study of *Bryophyllum pinnatum* (Lam.) Oken [2], resulting in a gap of knowledge on the phytochemical valorization of BD, BH, and BT. Moreover, to date, the study of phenolic compounds of *Bryophyllum* has been limited to their identification and description, with phenolic acids and flavonols as the main polyphenols found in this subgenus [5,36,37]. Little is known about the biosynthesis of phenolic compounds in *Bryophyllum* plants, and untargeted approaches are required for rapid and robust metabolic profiling of unexplored plant species [38]. The application of UM revealed new subfamilies of phenolic compounds in *Bryophyllum* plants, such as tyrosols, coumarins, catechols, lignans, stilbenes, and flavanols, together with the previously described phenolic acids, flavonols, and anthocyanins, although all of these subfamilies were already identified in elicited plant cell suspension cultures (PCSCs) from BD and BH [39]. According to our results (Figure 1), all three species have been determined as a potent source of phenolic compounds from different subfamilies.

Regarding the OPLS discriminant analyses performed, a genotype-dependent biosynthesis of phenolic compounds was revealed (Figure 2A). Although the three species are genetically close [40], our results indicated a differential phenolic profile for each one, in agreement with previous results, where these species showed different patterns related to important physiological processes, such as organogenesis [41], mineral nutrition [35], and the production of phenolic compounds [18].

The biosynthesis of phenolic compounds also followed an organ-dependent pattern (Figure 2B): anthocyanins, flavonols, and phenolic acids were the metabolites with the highest contribution to such discrimination. This compartmentalization may be a consequence of the physiological features associated with polyphenols since anthocyanins and flavonols usually accumulate in the aerial parts due to their role as protectants of the oxidative burst upon environmental stresses, UV-light absorbers, and pollinator attractants [42,43,44,45]. Our results are in agreement with previous reports, which determined that flavonols and anthocyanins predominantly accumulate in the aerial parts compared to roots [6,18]. Furthermore, the existence of specialized cell types within leaf tissues devoted to the storage of anthocyanins and other flavonoids, known as idioblasts, has been described in BD and BT [36,46].

The influence of genotype and organ on the biosynthesis of phenolic compounds, together with their interaction with nutrients, was assessed by the NFL predictive model (Table 1). The accuracy of the NFL-based prediction was assessed by the coefficient of determination of the training dataset (training set R^2^), together with the ANOVA parameters (F ratio > *f* critical), as described by Shao and co-workers [47]. Due this high predictability, the ML application enabled the identification of critical factors in the biosynthesis of each phenolic subfamily, thus conferring useful information that is easily understandable through the generation of the model rules (Table 2). Such a computer-based tool was successfully applied to predict the critical factors affecting the total phenolic and flavonoid contents of *Bryophyllum* cultured in vitro, revealing a significant influence of genotype and organs [16]. Concerning mineral nutrients, among the 18 different ions present in the universal Murashige and Skoog medium formulation [48], the NFL model only selected five as critical in the biosynthesis of phenolic compounds: sulfate, phosphate, calcium, magnesium, and copper (Table 1). This efficiency in the selection of mineral factors was previously demonstrated for other physiological processes, thus contributing to the optimization of PTC protocols [24,49,50].

With respect to the experimental design proposed, a reduction in both macronutrients and micronutrients from the universal Murashige and Skoog medium [48] was established (Section 4.2). Such a nutrient decrease was previously determined to exert a positive impact on the growth and multiplication of *Bryophyllum* cultured in vitro, motivated by their enhanced adaptation to arid regions where they are naturalized, with poor mineral accessibility that leads to low mineral requirements [35,51,52]. Furthermore, the reduction in mineral concentrations was already reported to promote an elicitor effect on *Bryophyllum* cultured in vitro [6,18], thus promoting an efficient strategy to assess the viability of this biotechnological system in the production of bioactive compounds. It must be considered that the modification of mineral concentrations may eventually have a significant effect on the buffer capacity, osmolarity, etc., thus promoting a possible effect on the modulation of phenolic compound biosynthesis that cannot be excluded.

Among the different subfamilies of phenolic compounds obtained by UM-mediated annotation, flavonols and anthocyanins were the only subfamilies that did not show a significant dependence on the mineral composition of the media employed, being predicted as a function of the interaction of genotype and organ (Table 1). The same behavior was previously reported for hydroethanolic *Bryophyllum* extracts, in which a genotype-dependent content of both flavonol and anthocyanin glycosides was observed [6]. Due to the high plasticity that flavonols and anthocyanins exhibit on plant physiology depending on mineral nutrition [7], our results suggested that the biosynthesis of these phenolic compounds could be stimulated by other mineral compositions different from those performed in this work for *Bryophyllum* spp. cultured in vitro.

The NFL model predicted that the biosynthesis of LMW compounds, which includes mainly tyrosols, coumarins, and catechols, was mainly influenced by sulfate concentration and, secondarily, by copper and phosphate. Sulfate was required in high concentrations (>0.94 mM) to promote LMW biosynthesis, probably due to its role in alleviating the autotoxicity caused by the prooxidant effects associated with the overaccumulation of tyrosols, as was demonstrated for hydroxytyrosol [53]. In addition, copper sulfate was reported as an elicitor of the biosynthesis of the coumarin scopoletin in PCSCs of *Angelica archangelica* [54], thus revealing the role of sulfate in plant stress tolerance [55]. The effect of copper and phosphate identifed by the NFL model also agreed with previous observations: a minimal copper concentration is required for the biosynthesis of tyrosols since this metal ion constitutes part of the active center of copper amine oxidase, which catalyzes the generation of hydroxytyrosol from dopamine [56]. In contrast, low phosphate requirements were predicted to enhance LMW biosynthesis, thus suggesting an inhibitory role of this salt, in agreement with the results reported for the roots of *Arabidopsis thaliana*, where coumarin biosynthesis is controlled by phosphate deficiency [57].

In the case of phenolic acids, the predictive model identified copper, in combination with genotype and organs, as the only nutrient involved in their biosynthesis. In this sense, phenolic acids predominantly accumulated in the aerial parts of the three *Bryophyllum* species, but copper was suggested to play a positive role in BD, causing an inhibitory effect on BH and BT (Figure 4). Thus, the results found for BD agree with those found for other medicinal plants, such as *Catharanthus roseus* [58], and *Raphanus sativus* [59], in which copper promoted the accumulation of phenolic acids. Such influence is driven by the copper-mediated stimulation of nitric oxide production, which acts as an inductor of phenylalanine ammonia lyase, driving the transformation of phenylalanine into cinnamic acid [58,60].

Lignan biosynthesis was predominantly found in BH, and it was enhanced by high sulfate concentrations, according to the predictive NFL model (>1.11 mM; Figure 4). The impact of sulfate, as a sulfur-containing ion, on lignan biosynthesis was already studied in *Linum album* hairy roots [61]. The authors proved that sulfur-containing signaling molecules, such as hydrogen sulfide, regulate the shift between lignan and flavonoid biosynthesis. This shifting behavior of copper might also play a role as a master regulator of lignan biosynthesis in *Bryophyllum* plants, due to the differential effects found on BH against its parental species, BD and BT.

Stilbene biosynthesis was predicted to be mainly affected by calcium concentration together with genotype and organ (Table 1). In this case, the genotypes showed the same pattern for lignans, since the mineral requirements for BD and BT were the same, being opposite to those predicted for BH (Figure 4). Thus, in the case of BD and BT, high calcium concentrations (>1.68 mM) were shown to enhance stilbene biosynthesis. Such an observation could be explained on the basis of the role of stilbenes as calcium complexing agents, thus providing evidence of the role of this subfamily of phenolic compounds as metal ion scavengers [62]. Moreover, this intraspecific differential role of calcium in stilbene biosynthesis was reported in *Vitis* spp., since calcium promoted stilbene biosynthesis in PCSCs of *Vitis amurensis* by stimulating stilbene synthase (STS) expression, via induction of calcium-dependent kinases [63], whereas calcium did not affect STS in PCSCs of *Vitis vinifera* [64].

Finally, flavanols constituted the last subfamily of phenolic compounds potentially affected by mineral nutrients in *Bryophyllum* spp., exhibiting a genotype-dependent accumulation of these compounds, as predicted by the NFL model (Table 1): high values for the flavanol content were observed in the roots of BT, mainly, and in the aerial parts of BD (Table 2). In addition, magnesium was found to exert a positive role on the flavanol accumulation in roots, showing an inhibitory effect on aerial parts (Figure 4). Since flavanols are the major phytoconstituents found in tea, the influence of magnesium on their production has been analyzed. The beneficial effects of magnesium on flavanol biosynthesis have been thoroughly investigated, being considered a relevant factor [65]. Thus, the exogenous soil addition of magnesium in open field experiments promoted the production of flavanols in black tea, via amino acid transferase induction [66]. In addition, molecular studies indicated that the metal complexing properties of catechins efficiently promoted the formation of stable complexes with magnesium [67], providing evidence of the role of magnesium as a regulator of catechin biosynthesis. On the other hand, the improved flavanol biosynthesis predicted for BT roots may be supported by the observations found for *Centaurea maculosa*, in which (-)-catechin is present in roots exudates developing an allelochemical effect responsible for the invasiveness of this species [44,68]. Since BT, together with other *Bryophyllum* species, is considered an invasive species [69], this enhanced production of flavanols by roots may contribute to the enhancement of such invasive mechanism.

## 4. Materials and Methods

### 4.1. Plant Material and Culture Conditions

Three different species from the *Bryophyllum* subgenus (genus *Kalanchoe*, Crassulaceae) were subjected to plant in vitro culture establishment: *Bryophyllum daigremontianum* Raym.-Hamet et Perr. (syn. *Kalanchoe daigremontiana*, BD), *Bryophyllum × houghtonii* D. B. Ward (*Bryophyllum daigremontianum × tubiflorum,* syn. *Kalanchoe × houghtonii*, BH), and *Bryophyllum tubiflorum* Harv. (syn. *Kalanchoe tubiflora*, BT).

Greenhouse-grown plants were selected as the source of epiphyllous plantlets. Thus, plantlets from the three species were collected, subjected to surface sterilization, and transferred to in vitro conditions, following the previously described protocol [70]. After disinfection, plantlets were cultured in pairs in glass culture vessels containing 25 mL of previously autoclaved Murashige and Skoog medium [48], supplemented with 3% (*w*/*v*) sucrose and solidified with 0.8% (*w*/*v*) agar at pH = 5.8. Then, plant cultures were randomly placed in growth chambers under a photoperiod of 16 h light and 8 h dark at 25 ± 1 °C. Periodical subcultures were performed every 12 weeks, by transferring new epiphyllous plantlets to fresh culture media.

### 4.2. Experimental Design

An experimental design for three variables at three, two, and seven levels was established: genotype (BD, BH, BT species), organ (aerial parts or roots), and culture media (7 formulations), resulting in a total of 42 treatments.

Seven culture media formulations, derived from Murashige and Skoog medium [48] were used for the nutrition experiment (Table 3). Murashige and Skoog-derived formulations contained a reduced content of both macronutrients (M) and micronutrients (µ) [35]. Treatments consisted of a half-strength medium (1/2MSM and 1/2MSµ), a quarter-strength medium (1/4MSM and 1/4MSµ), and an eighth-strength medium (1/8MSM and 1/8MSµ). Full-strength Murashige and Skoog medium was used as a control. To prevent additional interactions, EDTA-chelated iron, vitamin, and organic molecule concentrations were maintained for all the treatments as in the original formulation. All media were supplemented with 3% (*w*/*v*) sucrose and solidified with 0.8% (*w*/*v*) agar at pH = 5.8.

Epiphyllous plantlets with their own root system were selected from 12-week-old Murashige and Skoog-grown plants. The growth conditions were the same as previously described. Cultures were maintained in the same culture media formulation for successive four subcultures every 12 weeks. Plantlets were cultured in pairs, using 10 glass culture vessels per media formulation, accounting for a total of 20 replicates per treatment and species. After each subculture, plants were divided into aerial parts and roots and were separately stored at −20 °C until use.

### 4.3. Sample Preparation and Extraction

Collected plant materials were frozen-dried and powdered to obtain fine particles that were stored at −20 °C until extraction.

Sample extraction was performed using the solvent mixture MeOH:HCOOH:H_2_O (80:0.1:19.99) at a final concentration of 50 mg mL^−1^. The mixture was homogenized by a high-speed rotor (Polytron PT 1600-E) for 2 min and centrifuged at 8000 *g* for 10 min at 4 °C (Eppendorf 5810R, Hamburg, Germany). Supernatants were collected and filtered throughout syringe filters (pore size: 0.22 µm). Finally, extracts were transferred to vials and subsequently analyzed or stored at −20 °C until use.

### 4.4. Phenolic Profiling Using Untargeted Metabolomics

Phenolic compounds were profiled through an UM approach based on UHPLC-QTOF/MS, as previously reported [71,72]. Briefly, reverse phase chromatographic separation was achieved using a water–acetonitrile gradient, and then compounds were detected in SCAN mode (100–1200 m/z) at a nominal resolution of 40,000 FWHM. Quality controls were prepared by pooling each sample and were analyzed under the same chromatographic conditions, with acquisition using data-dependent tandem mass spectrometry [73].

The annotation of phenolic compounds was carried out using the Profinder B.07 software tool (Agilent Technologies), following mass (5-ppm tolerance) and retention time (0.05 min) alignment, as previously reported [71,74]. For this aim, the database exported from Phenol-Explorer 3.6 [75] was used, and annotation used the whole isotopic pattern of aligned features (namely, the monoisotopic accurate mass, isotopic ratio, and isotopic accurate spacing) [71,76]. Compounds were filtered by abundance (signal-to-noise >8) and by frequency (only the features annotated in 75% of replications within a treatment were used). A further step of annotation was then carried out in MS-DIAL 4.48 from tandem MS information, using publicly available MS/MS experimental spectra (Mass Bank of North America) and MS-Finder 3.50 for in-silico fragmentation (using Lipid Maps, FoodDB, and PlantCyc). The list of MS/MS compounds annotated is provided in the Supplementary (Table S1). Overall, compound annotation was done under Level-2 identification (putatively annotated compounds, COSMOS Metabolomics Standard Initiative) [77]. Total ion chromatograms were included in Figures S2 and S3.

Finally, identified phenolic compounds were classified into different subclasses and quantified using appropriate calibration curves for a reference standard per class. Results were expressed as equivalents of the reference compounds in mg/g of sample: cyanidin was selected for anthocyanins; catechin for flavanols; quercetin for flavonols; luteolin for flavones and other related flavonoids (flavanones and chalcones); sesamin for lignans; tyrosol for low-molecular-weight phenolics (LMW, including tyrosols, phenolic terpenes, quinones, coumarins, alkylphenols, and other phenylpropanoids); ferulic acid for phenolic acids; and resveratrol for stilbenes. Results were expressed as tyrosol equivalents (TE) for the LMW content, ferulic acid equivalents (FE) for the phenolic acid content, sesamin equivalents (SE) for the lignan content, resveratrol equivalents (RE) for the stilbene content, luteolin equivalents (LE) for the flavone content, quercetin equivalents (QE) for the flavonol content, cyanidin equivalents (CyE) for the anthocyanin content, and catechin equivalents (CaE) for the flavanol equivalents.

### 4.5. Statistical Analysis

Metabolomic profiling was performed with raw data using the software Agilent Mass Profiler Professional B.12.06. The data normalization was performed as previously indicated [78]: compounds were filtered according to their abundance and frequency, normalized at the 75th percentile, and baselined to the median of all samples. Bonferroni multiple testing correction was adopted in multivariate analyses. The obtained dataset was then exported to the software SIMCA 16 (Umetrics, Malmo, Sweden) for orthogonal projection to latent structures discriminant analysis (OPLS-DA). The cross-validation (CV) of the OPLS-DA model generated was developed using CV-ANOVA (α = 0.05), and its fitness and prediction ability were evaluated by the goodness-of-fit R^2^Y and goodness-of-prediction Q^2^Y parameters, respectively. Finally, to determine the most discriminant compounds, a variable importance in projection (VIP) analysis was performed setting a threshold VIP score >1.

Moreover, in order to assess the influence of genotypes, organs, and culture media formulations and their interactions on the production of phenolic compounds, a factorial ANOVA was performed, using the software STATISTICA v. 12 (StatSoft). The significance level was adjusted at α = 0.05.

### 4.6. Modeling Tools

After data collection, all experimental values were included in a database (Table S5) in which salts from culture media formulations were split into their containing ions to avoid ion confounding [79]. Consequently, 18 factors were selected as inputs for modeling: genotype, organ, and 16 ion concentrations from all media formulations tested. The parameters derived from phenolic quantification, including eight subclasses, were selected as outputs.

Data modeling was carried out using FormRules^®^ commercial software (v. 4.03; Intelligensys LTD, Cheshire, UK), as previously described [33]. The training parameters used for model establishment were described as follows: the Adaptive Spline Modeling of Data (ASMOD) was selected for the parameter minimization, as it improves model accuracy by reducing its complexity [80], with a ridge regression factor of 1 × 10^−6^. FormRules^®^ software includes several fitness criteria, such as Cross Validation (CV), Leave One Out Cross Validation (LOOCV), Bayesian Information Criterion (BIC), Minimal Description Length (MDL), and Structural Risk Minimization (SRM). All were tested in this study and the best fitted result, which provided the simplest and most intelligible rules with minimum generalization error, was SRM [35]. The rest of the training parameters were: C1 = 0.884, C2 = 4.8; number of set densities: 2; set densities: 2, 3; adapt nodes: TRUE; maximum inputs per submodel: 4; maximum nodes per input: 15. Thus, the model was divided into submodels in order to achieve an easier interpretation of results by the generation of “IF–THEN” rules [50,81]. Independent models were developed for each output, and a model assessment criterion was selected to avoid data over-fitting [22,82,83]. The application of NFL confers an advantage as a knowledge-obtaining tool since the predicted values for the inputs were expressed by words, ranging from low to high, combined with a corresponding membership degree, which takes values between 0 and 1 [29]. Furthermore, the predictive models for each output were quality-assessed in terms of the coefficient of determination of the training set (training set *R^2^*), expressed as a percentage given by Equation (1) [47], and mean square error, *MSE*, given by Equation (2).

(1) $$R^{2}=\left(1-\frac{\sum_{i=1}^{n}{\left(y_{i}-y_{i}^{\prime }\right)}^{2}}{\sum_{i=1}^{n}{\left(y_{i}-y_{i}^{\prime \prime }\right)}^{2}}\right)\times 100$$

(2) $$MSE=\left(\frac{\sum_{i=1}^{n}{\left(y_{i}-y_{i}^{\prime }\right)}^{2}}{n}\right)$$

where $y_{i}$ is the experimental value from the dataset, $y_{i}^{\prime }$ is the value predicted by the model, and $y_{i}^{\prime \prime }$ is the mean value of the dependent variable. Acceptable predicted values given by training set R^2^ range between 70–99.9% since higher values indicate model overfitting [22,84]. MSE represents the random error component associated with the built model, providing insight into model prediction due to a smaller incidence of random error [21,35]. Finally, to assess model accuracy, ANOVA was performed to check statistical differences between experimental and predicted data.

## 5. Conclusions

In this work, a combinatorial approach including three cutting-edge technologies, plant tissue culture, untargeted metabolomics, and machine learning, was established to gain insight into the biosynthesis of phenolic compounds of medicinal plants responsible for their associated therapeutic properties. The results indicate that *Bryophyllum* plants can be considered a promising source of phenolic compounds including the previously identified, flavonols, phenolic acids, and anthocyanins, together with new subfamilies reported for the first time in these species: tyrosols, catechols, lignans, stilbenes, flavones, flavanones, and flavanols. The knowledge derived from this investigation contributes to the phytochemical valorization of these unexplored medicinal plants. At the same, it may facilitate their exploitation as a natural source of bioactive compounds, promoting the large-scale application of *Bryophyllum* by-products to different biotechnological sectors with limitless purposes in food, cosmeceutical, and pharmacological industries. In addition, thanks to the robustness and plasticity of this multidisciplinary approach, the workflow proposed here can be applied to a plethora of poorly characterized plant species with medicinal potential, thus conferring a rapid and reliable methodology to provide insight into their biosynthetic capacity. In fact, the robustness and high performance associated with the combination of UM and ML presents limitless applications, thus opening new perspectives in the field of natural products research, facing the introduction of uncharacterized plant sources as efficient biofactories of health-promoting compounds of natural origin at an industrial level. The use of NFL as a predictive ML tool confers useful information about the key factors involved in complex processes, as was demonstrated here for the biosynthesis of phenolic compounds. This predicted information should be further validated in order to assess the knowledge conferred by this deep learning approach. Additionally, the application of other ML tools, such as genetic algorithms, will contribute to the computer-based optimization of such a multifactorial process. In this sense, this multidisciplinary strategy has proven extremely useful to improve the current paradigm of plant biotechnology, facilitating the knowledge on the production of phenolic compounds by medicinal plants, with the ability of being easily applied to economically important sectors, as is the case for agricultural, food, and related industries.

## Figures and Tables

**Table 1:** Critical factors identified by the neurofuzzy logic model and their quality parameters for each output. Bold letters indicate the input showing the strongest effect on every output.

Output	Submodel	Significant Inputs	Training set R^2^	MSE	F Ratio	df1, df2	*f* Critical (α = 0.05)
LMW	1	**Organ** × **SO_4_^2−^**	71.08	11.08	3.47	17, 24	2.07
	2	Genotype × Cu^2+^					
	3	HPO_4_^2−^					
Phenolic acids	1	Organ	72.12	5.52	8.02	10, 31	2.15
	2	**Genotype** × **Cu^2+^**					
Lignans	1	**Genotype** × **SO_4_^2−^** × **Organ**	73.32	5.19	6.64	12, 29	2.10
Stilbenes	1	**Ca^2+^** × **Organ** × **Genotype**	94.94	0.29	7.51	29, 12	2.47
	2	Genotype × HPO_4_^2−^ × Organ					
Flavones	-	-	68.77	2.71	12.84	6, 35	2.37
Flavonols	1	**Organ** × **Genotype**	74.49	0.41	17.03	6, 35	2.37
Anthocyanins	1	**Organ** × **Genotype**	77.15	0.51	19.70	6, 35	2.37
Flavanols	1	Mg^2+^ × Organ	78.04	1.16	12.63	9, 32	2.19
	2	**Organ** × **Genotype**					

**Table 2:** ‘IF–THEN’ rules generated by the neurofuzzy model. Bold rules indicate the inputs with the highest membership degree, showing the highest contribution for each output.

Rules		Gen ^1^	Organ ^2^	Ca^2+ 3^	Mg^2+ 3^	SO_4_^2− 3^	HPO_4_^2− 3^	Cu^2+ 3^		LMW	Phenolic Acids	Lignans	Stilbenes	Flavonols	Anthocyanins	Flavanols	MD ^4^
**1**	IF		**A**			**LOW**			THEN	**LOW**							**1.00**
2			A			MID LOW				LOW							1.00
3			A			MID HIGH				HIGH							1.00
4			A			HIGH				HIGH							1.00
5			R			LOW				LOW							1.00
6			R			MID LOW				LOW							1.00
7			R			MID HIGH				HIGH							1.00
**8**			**R**			**HIGH**				**HIGH**							**1.00**
9		BD						LOW		HIGH							1.00
10		BD						MID		LOW							1.00
11		BD						HIGH		LOW							1.00
12		BH						LOW		HIGH							1.00
13		BH						MID		HIGH							0.67
14		BH						HIGH		LOW							1.00
15		BT						LOW		HIGH							1.00
16		BT						MID		LOW							0.59
17		BT						HIGH		LOW							1.00
18							LOW			HIGH							1.00
19							HIGH			LOW							1.00
20	IF		A						THEN		HIGH						0.99
21			R								LOW						0.65
22		BD						LOW			LOW						0.62
**23**		**BD**						**MID**			**LOW**						**1.00**
**24**		**BD**						**HIGH**			**HIGH**						**0.58**
25		BH						LOW			LOW						0.84
26		BH						MID			LOW						0.52
27		BH						HIGH			LOW						0.75
28		BT						LOW			LOW						1.00
29		BT						MID			LOW						1.00
30		BT						HIGH			LOW						1.00
31	IF	BD	A			LOW			THEN			LOW					0.60
32		BD	R			LOW						LOW					0.80
33		BD	A			HIGH						LOW					0.88
34		BD	R			HIGH						LOW					0.69
35		BH	A			LOW						LOW					0.74
36		BH	R			LOW						LOW					0.84
**37**		**BH**	**A**			**HIGH**						**HIGH**					**0.84**
38		BH	R			HIGH						HIGH					0.51
39		BT	A			LOW						LOW					0.77
**40**		**BT**	**R**			**LOW**						**LOW**					**0.90**
41		BT	A			HIGH						LOW					0.80
42		BT	R			HIGH						LOW					0.73
43	IF	BD	A	LOW					THEN				LOW				1.00
**44**		**BH**	**A**	**LOW**									**HIGH**				**1.00**
45		BT	A	LOW									LOW				1.00
46		BD	R	LOW									LOW				1.00
47		BH	R	LOW									HIGH				1.00
48		BT	R	LOW									LOW				1.00
49		BD	A	HIGH									HIGH				1.00
**50**		**BH**	**A**	**HIGH**									**LOW**				**1.00**
51		BT	A	HIGH									HIGH				1.00
52		BD	R	HIGH									HIGH				0.65
53		BH	R	HIGH									LOW				1.00
54		BT	R	HIGH									HIGH				1.00
55		BD	A				LOW						HIGH				1.00
56		BD	R				LOW						HIGH				0.70
57		BD	A				MID						LOW				1.00
58		BD	R				MID						LOW				0.81
59		BD	A				HIGH						LOW				1.00
60		BD	R				HIGH						LOW				1.00
61		BH	A				LOW						LOW				1.00
62		BH	R				LOW						LOW				1.00
63		BH	A				MID						HIGH				1.00
64		BH	R				MID						LOW				0.52
65		BH	A				HIGH						HIGH				1.00
66		BH	R				HIGH						HIGH				1.00
67		BT	A				LOW						HIGH				1.00
68		BT	R				LOW						HIGH				1.00
69		BT	A				MID						LOW				0.85
70		BT	R				MID						LOW				0.93
71		BT	A				HIGH						LOW				1.00
72		BT	R				HIGH						LOW				1.00
**73**	IF	**BD**	**A**						THEN					**HIGH**			**0.76**
74		BD	R											LOW			0.83
75		BH	A											LOW			0.88
76		BH	R											LOW			0.86
77		BT	A											LOW			0.92
**78**		**BT**	**R**											**LOW**			**0.94**
**79**	IF	**BD**	**A**						THEN						**HIGH**		**0.78**
80		BH	A												HIGH		0.57
81		BT	A												LOW		0.72
82		BD	R												LOW		0.84
83		BH	R												LOW		0.84
**84**		**BT**	**R**												**LOW**		**0.96**
85	IF		A		LOW				THEN							LOW	0.71
86			R		LOW											HIGH	0.74
87			A		HIGH											LOW	0.76
88			R		HIGH											LOW	0.86
89		BD	A													HIGH	0.74
90		BD	R													LOW	0.98
91		BH	A													LOW	1.00
92		BH	R													LOW	0.90
**93**		**BT**	**A**													**LOW**	**1.00**
**94**		**BT**	**R**													**HIGH**	**0.76**

^1^ “Gen” represents genotypes. BD: *B. daigremontianum*, BH: *B. × houghtonii*, BT: *B. tubiflorum*. ^2^ Organ: “A” represents aerial parts and “R” represents roots. ^3^ The values for each input were ranked as “LOW”, “MID LOW”, “MID HIGH” and “HIGH” by the model, and the values are shown in Figure S1. ^4^ “MD” represents the membership degree.

**Table 3:** The salt composition (mg L^−1^) of the seven culture media formulations used in this study based on the Murashige and Skoog medium formulation.

	Salts (mg L^−1^)	MS Control	1/2MSM	1/4MSM	1/8MSM	1/2MSµ	1/4MSµ	1/8MSµ
Macro-nutrients	KNO_3_	1900	950	475	237.5	1900	1900	1900
	NH_4_NO_3_	1650	825	412.5	206.3	1650	1650	1650
	CaCl_2_ 2H_2_O	440	220	110	55	440	440	440
	MgSO_4_ 7H_2_O	370	185	92.5	46.3	370	370	370
	KH_2_PO_4_	170	85	42.5	21.3	170	170	170
Micronutrients	MnSO_4_ 4H_2_O	22.3	22.3	22.3	22.3	11.2	5.6	2.8
	ZnSO_4_ 7H_2_O	8.6	8.6	8.6	8.6	4.3	2.2	1.1
	H_3_BO_3_	6.2	6.2	6.2	6.2	3.1	1.6	0.78
	KI	0.83	0.83	0.83	0.83	0.42	0.21	0.11
	Na_2_MoO_4_ 2H_2_O	0.25	0.25	0.25	0.25	0.13	0.063	0.03
	CuSO_4_ 5H_2_O	0.025	0.025	0.025	0.025	0.013	0.0063	0.0031
	CoCl_2_ 6H_2_O	0.025	0.025	0.025	0.025	0.013	0.0063	0.0031
Iron source	Na_2_EDTA	37.25	37.25	37.25	37.25	37.25	37.25	37.25
	FeSO_4_ 7H_2_O	27.85	27.85	27.85	27.85	27.85	27.85	27.85

## Data Availability

The data presented in this study are available in article and Supplementary Materials.

## Supplementary Materials

The following are available online at https://www.mdpi.com/article/10.3390/plants10112430/s1, Figure S1: Representation of nutrient concentration ranges obtained by FormRules^®^ for each output. (A) SO_4_^2−^ concentration for LMW. (B) Cu^2+^ concentration for LMW and phenolic acids. (C) HPO_4_^2−^ concentration for LMW. (D) SO_4_^2−^ concentration for lignans. (E) Ca^2+^ concentration for stilbenes. (F) HPO_4_^2−^ concentration for stilbenes. (G) Mg^2+^ concentration for flavanols; Figure S2: representative chromatograms of leaf samples under the different test conditions; Figure S3: representative chromatograms of root samples under the different test conditions; Table S1: sheet 1, Dataset of annotated compounds; sheet 2, list of compounds annotated by MS/MS; Table S2: Statistical parameters associated with the factorial ANOVA performed for each subfamily involved in the semiquantification of phenolic compounds. df, degrees of freedom; SS, the sum of squares; MS, mean squares.; Table S3: Metabolites with the highest contribution to discrimination between *Bryophyllum* genotypes, according to the OPLS-DA predictive model, followed by VIP selection method. Metabolites are grouped into their subfamilies and are accompanied by their VIP score and standard error; Table S4: Metabolites with the highest contribution to discrimination between organs: aerial parts and roots, according by the OPLS-DA predictive model, followed by VIP selection method. Metabolites are grouped into their subfamilies and are accompanied by their VIP score and standard error; Table S5: Dataset subjected to NFL modeling.

## References

[1] García-Pérez P., Barreal M., Dios L.R.-D., Cameselle-Teijeiro J., Gallego P., Rahman A. (2019). Bioactive Natural Products from the Genus *Kalanchoe* as Cancer Chemopreventive Agents: A Review. Studies in Natural Products Chemistry.

[2] García-Pérez P., Lozano-Milo E., Landin M., Gallego P.P. (2020). From Ethnomedicine to Plant Biotechnology and Machine Learning: The Valorization of the Medicinal Plant *Bryophyllum* sp. Pharmaceuticals.

[3] Lozano-Milo E., García-Pérez P., Gallego P.P. (2020). Narrative review of production of antioxidants and anticancer compounds from *Bryophyllum* spp. (*Kalanchoe*) using plant cell tissue culture. Longhua Chin. Med..

[4] Katrucha E.M., Lopes J., Paim M., dos Santos J.C., Siebert D.A., Micke G.A., Vitali L., Alberton M.D., Tenfen A. (2020). Phenolic profile by HPLC-ESI-MS/MS and enzymatic inhibitory effect of *Bryophyllum delagoense*. Nat. Prod. Res..

[5] Stefanowicz-Hajduk J., Asztemborska M., Krauze-Baranowska M., Godlewska S., Gucwa M., Moniuszko-Szajwaj B., Stochmal A., Ochocka J.R. (2020). Identification of Flavonoids and Bufadienolides and Cytotoxic Effects of *Kalanchoe* daigremontiana Extracts on Human Cancer Cell Lines. Planta Medica.

[6] García-Pérez P., Ayuso M., Lozano-Milo E., Pereira C., Dias M.I., Ivanov M., Calhelha R.C., Soković M., Ferreira I.C.F.R., Barros L. (2021). Phenolic profiling and in vitro bioactivities of three medicinal *Bryophyllum* plants. Ind. Crop. Prod..

[7] García-Pérez P., Losada-Barreiro S., Bravo-Díaz C., Gallego P.P. (2020). Exploring the use of *Bryophyllum* as natural source of bioactive compounds with antioxidant activity to prevent lipid oxidation of fish oil-in-water emulsions. Plants.

[8] Tohge T., Watanabe M., Hoefgen R., Fernie A.R. (2013). Shikimate and Phenylalanine Biosynthesis in the Green Lineage. Front. Plant Sci..

[9] Saito K., Yonekura-Sakakibara K., Nakabayashi R., Higashi Y., Yamazaki M., Tohge T., Fernie A.R. (2013). The flavonoid biosynthetic pathway in Arabidopsis: Structural and genetic diversity. Plant Physiol. Biochem..

[10] Bogucka-Kocka A., Zidorn C., Kasprzycka M., Szymczak G., Szewczyk K. (2018). Phenolic acid content, antioxidant and cytotoxic activities of four Kalanchoë species. Saudi J. Biol. Sci..

[11] Hasanpour M., Iranshahy M., Iranshahi M. (2020). The application of metabolomics in investigating anti-diabetic activity of medicinal plants. Biomed. Pharmacother..

[12] Wolfender J., Queiroz E.F., Allard P. (2020). Massive metabolite profiling of natural extracts for a rational prioritization of bioactive natural products: A paradigm shift in pharmacognosy. Food Front..

[13] Marchev A.S., Georgiev M.I. (2020). Plant In Vitro Systems as a Sustainable Source of Active Ingredients for Cosmeceutical Application. Molecules.

[14] Dias M.I., Sousa M.J., Alves R.C., Ferreira I.C. (2016). Exploring plant tissue culture to improve the production of phenolic compounds: A review. Ind. Crop. Prod..

[15] Espinosa-Leal C.A., Puente-Garza C.A., García-Lara S. (2018). In vitro plant tissue culture: Means for production of biological active compounds. Planta.

[16] Phillips G.C., Garda M. (2019). Plant tissue culture media and practices: An overview. Vitr. Cell. Dev. Biol. Anim..

[17] Da Silva J.A.T., Nezami-Alanagh E., Barreal M.E., Kher M.M., Wicaksono A., Gulyás A., Hidvégi N., Magyar-Tábori K., Mendler-Drienyovszki N., Márton L. (2020). Shoot tip necrosis of in vitro plant cultures: A reappraisal of possible causes and solutions. Planta.

[18] García-Pérez P., Lozano-Milo E., Landín M., Gallego P.P. (2020). Combining Medicinal Plant In Vitro Culture with Machine Learning Technologies for Maximizing the Production of Phenolic Compounds. Antioxidants.

[19] Gallego P.P., Gago J., Landín M., Suzuki K. (2011). Artificial neural networks technology to model and predict plant biology process. Meth-odological Advances and Biomedical Applications.

[20] Nezami-Alanagh E., Garoosi G.-A., Landin M., Gallego P.P. (2018). Combining DOE with Neurofuzzy Logic for Healthy Mineral Nutrition of Pistachio Rootstocks in vitro Culture. Front. Plant Sci..

[21] Hesami M., Jones A.M.P. (2020). Application of artificial intelligence models and optimization algorithms in plant cell and tissue culture. Appl. Microbiol. Biotechnol..

[22] Landín M., Rowe R.C., York P. (2009). Advantages of neurofuzzy logic against conventional experimental design and statistical analysis in studying and developing direct compression formulations. Eur. J. Pharm. Sci..

[23] Gago J., Martínez-Núñez L., Landín M., Gallego P.P. (2010). Artificial neural networks as an alternative to the traditional statistical methodology in plant research. J. Plant Physiol..

[24] Niazian M., Nalousi A.M. (2020). Artificial polyploidy induction for improvement of ornamental and medicinal plants. Plant Cell Tissue Organ Cult..

[25] Ayuso M., Ramil-Rego P., Landin M., Gallego P.P., Barreal M.E. (2017). Computer-Assisted Recovery of Threatened Plants: Keys for Breaking Seed Dormancy of Eryngium viviparum. Front. Plant Sci..

[26] Hameg R., Arteta T.A., Landin M., Gallego P.P., Barreal M.E. (2020). Modeling and Optimizing Culture Medium Mineral Composition for in vitro Propagation of Actinidia arguta. Front. Plant Sci..

[27] Nezami-Alanagh E., Garoosi G.-A., Landin M., Gallego P.P. (2019). Computer-based tools provide new insight into the key factors that cause physiological disorders of pistachio rootstocks cultured in vitro. Sci. Rep..

[28] Landin M., Rowe R.C. (2013). Artificial neural networks technology to model, understand, and optimize drug formulations. Formulation Tools for Pharmaceutical Development.

[29] Gago J., Pérez-Tornero O., Landin M., Burgos L., Gallego P.P. (2011). Improving knowledge of plant tissue culture and formulation bv neurofuzzy logic: A practical case of data mining using apricot databases. J. Plant Physiol..

[30] García-Pérez P., Losada-Barreiro S., Gallego P.P., Bravo-Díaz C. (2019). Cyclodextrin-elicited *Bryophyllum* suspension cultured cells: Enhancement of the production of bioactive compounds. Int. J. Mol. Sci..

[31] Di Lorenzo C., Colombo F., Biella S., Stockley C., Restani P. (2021). Polyphenols and Human Health: The Role of Bioavailability. Nutrients.

[32] Efenberger-Szmechtyk M., Nowak A., Czyżowska A. (2021). Plant extracts rich in polyphenols: Antibacterial agents and natural preservatives for meat and meat products. Crit. Rev. Food Sci. Nutr..

[33] Chandran H., Meena M., Barupal T., Sharma K. (2020). Plant tissue culture as a perpetual source for production of industrially important bioactive compounds. Biotechnol. Rep..

[34] Marchev A.S., Yordanova Z.P., Georgiev M.I. (2020). Green (cell) factories for advanced production of plant secondary metabolites. Crit. Rev. Biotechnol..

[35] García-Pérez P., Lozano-Milo E., Landin M., Gallego P.P. (2020). Machine Learning Unmasked Nutritional Imbalances on the Medicinal Plant *Bryophyllum* sp. Cultured in vitro. Front. Plant Sci..

[36] Casanova J.M., Nascimento L.B.D.S., Casanova L.M., Leal-Costa M.V., Costa S.S., Tavares E.S. (2020). Differential Distribution of Flavonoids and Phenolic Acids in Leaves of *Kalanchoe* delagoensis Ecklon & Zeyher (Crassulaceae). Microsc. Microanal..

[37] Stefanowicz-Hajduk J., Hering A., Gucwa M., Hałasa R., Soluch A., Kowalczyk M., Stochmal A., Ochocka R. (2020). Biological activities of leaf extracts from selected *Kalanchoe* species and their relationship with bufadienolides content. Pharm. Biol..

[38] Gajula S.N.R., Nanjappan S. (2020). Metabolomics: A Recent Advanced Omics Technology in Herbal Medicine Research.

[39] García-Pérez P., Miras-Moreno B., Lucini L., Gallego P.P. (2021). The metabolomics reveals intraspecies variability of bioactive compounds in elicited suspension cell cultures of three *Bryophyllum* species. Ind. Crop. Prod..

[40] Herrando-Moraira S., Vitales D., Nualart N., Gómez-Bellver C., Ibáñez N., Massó S., Cachón-Ferrero P., González-Gutiérrez P.A., Guillot D., Herrera I. (2020). Global distribution patterns and niche modelling of the invasive *Kalanchoe* × *houghtonii* (Crassulaceae). Sci. Rep..

[41] García-Pérez P., Lozano-Milo E., Landín M., Gallego P.P. (2020). Machine Learning Technology Reveals the Concealed Interactions of Phytohormones on Medicinal Plant In Vitro Organogenesis. Biomolecules.

[42] Li D.-D., Ni R., Wang P.-P., Zhang X.-S., Wang P.-Y., Zhu T.-T., Sun C.-J., Liu C.-J., Lou H.-X., Cheng A.-X. (2020). Molecular Basis for Chemical Evolution of Flavones to Flavonols and Anthocyanins in Land Plants. Plant Physiol..

[43] Agati G., Brunetti C., Fini A., Gori A., Guidi L., Landi M., Sebastiani F., Tattini M. (2020). Are Flavonoids Effective Antioxidants in Plants? Twenty Years of Our Investigation. Antioxidants.

[44] Shah A., Smith D.L. (2020). Flavonoids in Agriculture: Chemistry and Roles in, Biotic and Abiotic Stress Responses, and Microbial Associations. Agronomy.

[45] García-Pérez P., Lozano-Milo E., Gallego P.P., Tojo C., Losada-Barreiro S., Bravo-Díaz C., Milani J. (2019). Plant antioxidants in food emul-sions. Some New Aspects of Colloidal Systems in Foods.

[46] Chernetskyy M., Woźniak A., Skalska-Kamińska A., Żuraw B., Blicharska E., Rejdak R., Donica H., Weryszko-Chmielewska E. (2018). Structure of leaves and phenolic acids in Kalanchoë daigremontiana Raym.-Hamet & H. Perrier. Acta Sci. Pol. Hortorum Cultus.

[47] Shao Q., Rowe R.C., York P. (2006). Comparison of neurofuzzy logic and neural networks in modelling experimental data of an immediate release tablet formulation. Eur. J. Pharm. Sci..

[48] Murashige T., Skoog F. (1962). A Revised Medium for Rapid Growth and Bio Assays with Tobacco Tissue Cultures. Physiol. Plant..

[49] Hesami M., Naderi R., Tohidfar M., Yoosefzadeh-Najafabadi M. (2020). Development of support vector machine-based model and comparative analysis with artificial neural network for modeling the plant tissue culture procedures: Effect of plant growth regulators on somatic embryogenesis of chrysanthemum, as a case study. Plant Methods.

[50] Nezami-Alanagh E., Garoosi G.-A., Maleki S., Landin M., Gallego P.P. (2017). Predicting optimal in vitro culture medium for *Pistacia vera* micropropagation using neural networks models. Plant Cell Tissue Organ Cult..

[51] Delgado-Baquerizo M., Maestre F.T., Gallardo A., Bowker M.A., Wallenstein M., Quero J.L., Ochoa V., Gozalo B., García-Gómez M., Soliveres S. (2013). Decoupling of soil nutrient cycles as a function of aridity in global drylands. Nat. Cell Biol..

[52] Pereira P.N., Cushman J.C. (2019). Exploring the Relationship between Crassulacean Acid Metabolism (CAM) and Mineral Nutrition with a Special Focus on Nitrogen. Int. J. Mol. Sci..

[53] Bertelli M., Kiani A.K., Paolacci S., Manara E., Kurti D., Dhuli K., Bushati V., Miertus J., Pangallo D., Baglivo M. (2020). Hydroxytyrosol: A natural compound with promising pharmacological activities. J. Biotechnol..

[54] Siatka T., Chlebekb J., Hostalkova A. (2017). Copper(II) Sulfate Stimulates Scopoletin Production in Cell Suspension Cultures of Angelica archangelica. Nat. Prod. Commun..

[55] Mosher S., Seybold H., Rodriguez P., Stahl M., Davies K.A., Dayaratne S., Morillo S.A., Wierzba M., Favery B., Keller H. (2013). The tyrosine-sulfated peptide receptors PSKR1 and PSY1R modify the immunity of Arabidopsis to biotrophic and necrotrophic pathogens in an antagonistic manner. Plant J..

[56] Zhou Y., Zhu J., Shao L., Guo M. (2020). Current advances in acteoside biosynthesis pathway elucidation and biosynthesis. Fitoterapia.

[57] Chutia R., Abel S., Ziegler J. (2019). Iron and Phosphate Deficiency Regulators Concertedly Control Coumarin Profiles in Arabidopsis thaliana Roots During Iron, Phosphate, and Combined Deficiencies. Front. Plant Sci..

[58] Liu S., Yang R., Pan Y., Ren B., Chen Q., Li X., Xiong X., Tao J., Cheng Q., Ma M. (2016). Beneficial behavior of nitric oxide in copper-treated medicinal plants. J. Hazard. Mater..

[59] Sgherri C., Cosi E., Navari-Izzo F. (2003). Phenols and antioxidative status of *Raphanus sativus* grown in copper excess. Physiol. Plant..

[60] Kováčik J., Grúz J., Klejdus B., Štork F., Marchiosi R., Ferrarese-Filho O. (2010). Lignification and related parameters in copper-exposed *Matricaria chamomilla* roots: Role of H_2_O_2_ and NO in this process. Plant Sci..

[61] Fakhari S., Sharifi M., de Michele R., Ghanati F., Safaie N., Sadeghnezhad E. (2019). Hydrogen sulfide directs metabolic flux towards the lignan biosynthesis in Linum album hairy roots. Plant Physiol. Biochem..

[62] Causero A., Elsen H., Ballmann G., Escalona A., Harder S. (2017). Calcium stilbene complexes: Structures and dual reactivity. Chem. Commun..

[63] Aleynova O.A., Dubrovina A.S., Kiselev K.V. (2017). Activation of stilbene synthesis in cell cultures of *Vitis amurensis* by calcium-dependent protein kinases VaCPK1 and VaCPK26. Plant Cell Tissue Organ Cult..

[64] Martins V., Garcia A., Costa C., Sottomayor M., Gerós H. (2018). Calcium- and hormone-driven regulation of secondary metabolism and cell wall enzymes in grape berry cells. J. Plant Physiol..

[65] Cheng H., Wei K., Wang L., Hagiwara K.A., Wright A.D. (2015). Tea Leaf Age, Shade and Characteristic Levels of l-Theanine, Caffeine, (-)-Epigallocatechin Gallate (EGCG), (-)-Epigallocatechin (EGC), (-)-Epicatechin (EC), and (-)-Epicatechin Gallate (ECG). Processing and Impact on Active Components in Food.

[66] Jayaganesh S., Venkatesan S. (2009). Impact of Magnesium Sulphate on Biochemical and Quality Constituents of Black Tea. Am. J. Food Technol..

[67] Chen Z., Yu T., Zhou B., Wei J., Fang Y., Lu J., Guo L., Chen W., Liu Z.-P., Luo J. (2016). Mg(II)-Catechin nanoparticles delivering siRNA targeting EIF5A2 inhibit bladder cancer cell growth in vitro and in vivo. Biomaterials.

[68] Walker T.S., Bais H.P., Grotewold E., Vivanco J.M. (2003). Root Exudation and Rhizosphere Biology. Plant Physiol..

[69] Guerra-García A., Golubov J., Mandujano M.C. (2014). Invasion of *Kalanchoe* by clonal spread. Biol. Invasions.

[70] García-Pérez P., Losada-Barreiro S., Gallego P.P., Bravo-Díaz C. (2019). Adsorption of gallic acid, propyl gallate and polyphenols from *Bryophyllum* extracts on activated carbon. Sci. Rep..

[71] Arriola N.D.A., Chater P.I., Wilcox M., Lucini L., Rocchetti G., Dalmina M., Pearson J.P., Amboni R.D.D.M.C. (2019). Encapsulation of stevia rebaudiana Bertoni aqueous crude extracts by ionic gelation—Effects of alginate blends and gelling solutions on the polyphenolic profile. Food Chem..

[72] Rocchetti G., Pagnossa J.P., Blasi F., Cossignani L., Piccoli R.H., Zengin G., Montesano D., Cocconcelli P.S., Lucini L. (2020). Phenolic profiling and in vitro bioactivity of Moringa oleifera leaves as affected by different extraction solvents. Food Res. Int..

[73] Rocchetti G., Michelini S., Pizzamiglio V., Masoero F., Lucini L. (2021). A combined metabolomics and peptidomics approach to discriminate anomalous rind inclusion levels in Parmigiano Reggiano PDO grated hard cheese from different ripening stages. Food Res. Int..

[74] Rocchetti G., Lucini L., Rodriguez J.M.L., Barba F.J., Giuberti G. (2019). Gluten-free flours from cereals, pseudocereals and legumes: Phenolic fingerprints and in vitro antioxidant properties. Food Chem..

[75] Rothwell J.A., Pérez-Jiménez J., Neveu V., Medina-Remón A., M’Hiri N., García-Lobato P., Manach C., Knox C., Eisner R., Wishart D.S. (2013). Phenol-Explorer 3.0: A major update of the Phenol-Explorer database to incorporate data on the effects of food processing on polyphenol content. Database.

[76] Lucini L., Rocchetti G., Kane D., Trevisan M. (2017). Phenolic fingerprint allows discriminating processed tomato products and tracing different processing sites. Food Control..

[77] Salek R.M., Steinbeck C., Viant M.R., Goodacre R., Dunn W.B. (2013). The role of reporting standards for metabolite annotation and identification in metabolomic studies. GigaScience.

[78] Zhang L., Saber F.R., Rocchetti G., Zengin G., Hashem M.M., Lucini L. (2021). UHPLC-QTOF-MS based metabolomics and biological activities of different parts of *Eriobotrya japonica*. Food Res. Int..

[79] Niedz R.P., Evens T.J. (2006). A solution to the problem of ion confounding in experimental biology. Nat. Methods.

[80] Kavli T., Weyer E. ASMOD (Adaptive Spline Modelling of Observation Data): Some theoretical and experimental results. Proceedings of the IEE Colloquium on Advances in Neural Networks for Control and Systems.

[81] Arteta T., Hameg R., Landin M., Gallego P.P., Barreal M.E. (2018). Neural networks models as decision-making tool for in vitro proliferation of hardy kiwi. Eur. J. Hortic. Sci..

[82] Colbourn E., Rowe R. (2005). Encyclopaedia of Pharmaceutical Technology.

[83] Vapnik V. (1992). Principles of Risk Minimization for Learning Theory. Advances in Neural Information Processing Systems.

[84] Colbourn E.A., Rowe R.C. (2009). Novel approaches to neural and evolutionary computing in pharmaceutical formulation: Challenges and new possibilities. Future Med. Chem..

