# Supplementary material for: The Combination of Untargeted Metabolomics and Machine Learning Predicts the Biosynthesis of Phenolic Compounds in Bryophyllum Medicinal Plants (Genus Kalanchoe)
Source: Plants (Basel). 2021 Nov 10;10(11):2430. doi: 10.3390/plants10112430 (PMC8620224; doi:10.3390/plants10112430)
Supplement: Supplementary file 1 [file plants-10-02430-s001.zip › Supplementary material_Figure S1_tableS2-4.pdf]

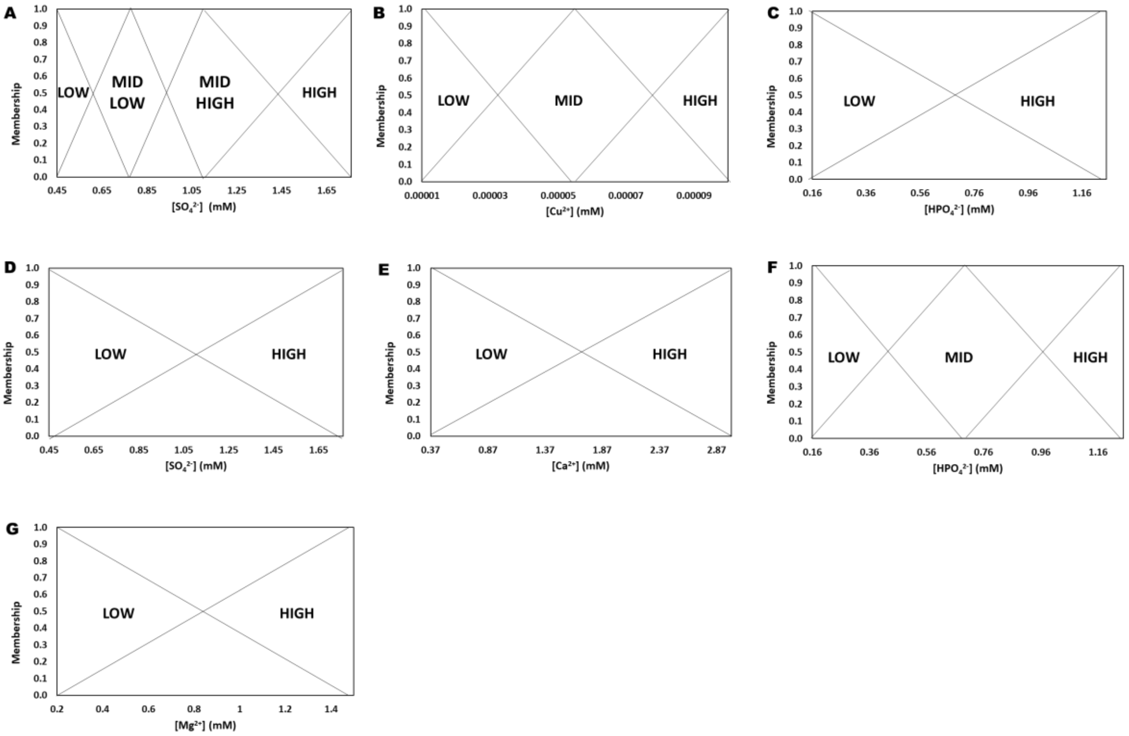

Figure S1. Representation of nutrient concentration ranges obtained by FormRules® for each output. **A.** SO_4^{2-} concentration for LMW. **B.** Cu^{2+} concentration for LMW and phenolic acids. **C.** HPO_4^{2-} concentration for LMW. **D.** SO_4^{2-} concentration for lignans. **E.** Ca^{2+} concentration for stilbenes. **F.** HPO_4^{2-} concentration for stilbenes. **G.** Mg^{2+} concentration for flavanols.

Table S2. Statistical parameters associated with the factorial ANOVA performed for each subfamily involved in the semiquantification of phenolic compounds. df, degrees of freedom; SS, sum of squares; MS, mean squares.

Anthocyanins	df	SS	MS	F	p
Intercept	1	342.3603	342.3603	3270.867	<0.001
Species	2	43.8140	21.9070	209.297	<0.001
Organs	1	119.5854	119.5854	1142.503	<0.001
Media	6	11.6997	1.9500	18.630	<0.001
Species*Organs	2	15.6469	7.8234	74.744	<0.001
Species*Media	12	16.4770	1.3731	13.118	<0.001
Organs*Media	6	8.6905	1.4484	13.838	<0.001
Species*Organs*Media	12	16.2269	1.3522	12.919	<0.001
Error	84	8.7922	0.1047		

Flavones	df	SS	MS	F	p
Intercept	1	2660.476	2660.476	6657.866	<0.001
Species	2	124.617	62.308	155.927	<0.001
Organs	1	375.836	375.836	940.534	<0.001
Media	6	45.558	7.593	19.002	<0.001
Species*Organs	2	129.247	64.624	161.721	<0.001
Species*Media	12	95.798	7.983	19.978	<0.001
Organs*Media	6	49.000	8.167	20.437	<0.001
Species*Organs*Media	12	90.135	7.511	18.797	<0.001
Error	84	33.566	0.400		

Flavanols	df	SS	MS	F	p
Intercept	1	897.4981	897.4981	5879.131	<0.001
Species	2	83.8663	41.9331	274.686	<0.001
Organs	1	13.6812	13.6812	89.620	<0.001
Media	6	76.8163	12.8027	83.865	<0.001
Species*Organs	2	295.2142	147.6071	966.912	<0.001
Species*Media	12	92.6264	7.7189	50.563	<0.001
Organs*Media	6	41.7133	6.9522	45.541	<0.001
Species*Organs*Media	12	58.0269	4.8356	31.676	<0.001
Error	84	12.8233	0.1527		

Flavonols	df	SS	MS	F	p
Intercept	1	263.9778	263.9778	4673.578	<0.001
Species	2	66.2751	33.1376	586.682	<0.001
Organs	1	19.3939	19.3939	343.358	<0.001
Media	6	4.0282	0.6714	11.886	<0.001
Species*Organs	2	40.2435	20.1217	356.244	<0.001
Species*Media	12	12.1194	1.0100	17.881	<0.001
Organs*Media	6	12.5361	2.0894	36.991	<0.001
Species*Organs*Media	12	15.8799	1.3233	23.429	<0.001
Error	84	4.7446	0.0565		

Lignans	df	SS	MS	F	p
Intercept	1	7983.641	7983.641	11820.59	<0.001
Species	2	565.681	282.841	418.77	<0.001
Organs	1	39.381	39.381	58.31	<0.001
Media	6	156.091	26.015	38.52	<0.001
Species*Organs	2	128.296	64.148	94.98	<0.001
Species*Media	12	384.444	32.037	47.43	<0.001

Organs*Media	6	57.073	9.512	14.08	<0.001
Species*Organs*Media	12	359.270	29.939	44.33	<0.001
Error	84	56.734	0.675		

LMW	df	SS	MS	F	p
Intercept	1	52319.80	52319.80	14024.02	<0.001
Species	2	280.29	140.14	37.57	<0.001
Organs	1	765.02	765.02	205.06	<0.001
Media	6	385.53	64.25	17.22	<0.001
Species*Organs	2	94.04	47.02	12.60	<0.001
Species*Media	12	483.97	40.33	10.81	<0.001
Organs*Media	6	330.79	55.13	14.78	<0.001
Species*Organs*Media	12	418.85	34.90	9.36	<0.001
Error	84	313.38	3.73		

Phenolic acids	df	SS	MS	F	p
Intercept	1	14926.44	14926.44	13754.96	<0.001
Species	2	213.26	106.63	98.26	<0.001
Organs	1	775.53	775.53	714.66	<0.001
Media	6	61.72	10.29	9.48	<0.001
Species*Organs	2	49.40	24.70	22.76	<0.001
Species*Media	12	426.94	35.58	32.79	<0.001
Organs*Media	6	34.68	5.78	5.33	<0.001
Species*Organs*Media	12	268.01	22.33	20.58	<0.001
Error	84	91.15	1.09		

Stilbenes	df	SS	MS	F	p
Intercept	1	345.8778	345.8778	2407.653	<0.001
Species	2	19.3720	9.6860	67.424	<0.001
Organs	1	7.6822	7.6822	53.475	<0.001
Media	6	46.2632	7.7105	53.673	<0.001
Species*Organs	2	5.5940	2.7970	19.470	<0.001
Species*Media	12	36.3055	3.0255	21.060	<0.001
Organs*Media	6	25.6249	4.2708	29.729	<0.001
Species*Organs*Media	12	67.8721	5.6560	39.371	<0.001
Error	84	12.0672	0.1437		

Table S3. Metabolites with the highest contribution to discrimination between bryophyllum genotypes, according by the OPLS-DA predictive model, followed by VIP selection method. Metabolites are grouped into their subfamilies and accompanied by their VIP score and standard error.

**Compounds**	**VIP score**
**LMW**	
Ligstroside	1.77 ± 0.30
Umbelliferone	1.55 ± 0.35
p-HPEA-EDA	1.41 ± 0.38
Bisdemethoxycurcumin	1.35 ± 0.42
Oleuropein	1.28 ± 0.53
Thymol	1.24 ± 0.29
Juglone	1.19 ± 0.19
Carvacrol	1.15 ± 0.41
2,3-Dihydroxy-1-guaiacylpropanone	1.06 ± 0.21
Carnosic acid	1.04 ± 0.16
5-pentadecylresorcinol	1.03 ± 0.61
Coumarin	1.02 ± 0.32
Scopoletin	1.01 ± 0.30
Anethole	1.00 ± 0.36
**Phenolic acids**	
Caffeic acid	1.55 ± 0.34
Ferulic acid 4-O-glucoside	1.49 ± 0.49
Sinapine	1.44 ± 0.32
4-Caffeoylquinic acid	1.42 ± 0.24
4,5-dicaffeoylquinic acid	1.40 ± 0.38
3-sinapoylquinic acid	1.38 ± 0.37
4-Hydroxybenzoic acid 4-O-glucoside	1.36 ± 0.44
Chicoric acid	1.24 ± 0.31
Gallic acid	1.24 ± 0.41
2-S-Glutathionyl caftaric acid	1.21 ± 0.40
Schottenol ferulate	1.19 ± 0.43
Sitosterol ferulate	1.18 ± 0.43
Feruloyl glucose	1.18 ± 0.46
Galloyl glucose	1.18 ± 0.20
Gallic acid 4-O-glucoside	1.16 ± 0.20
Avenanthramide K	1.15 ± 0.23
m-Coumaric acid	1.13 ± 0.29
Avenanthramide 2p	1.10 ± 0.27
3-Feruloylquinic acid	1.07 ± 0.54
4-Feruloylquinic acid	1.06 ± 0.55
Sinapic acid	1.04 ± 0.28
Gentisic acid	1.03 ± 0.48
**Lignans**	
Episesaminol	1.66 ± 0.36
Sesaminol	1.66 ± 0.37
Sesamolin	1.62 ± 0.36
Syringaresinol	1.46 ± 0.26
Isohydroxymatairesinol	1.44 ± 0.61
1-acetoxypinoresinol	1.26 ± 0.29
Todolactol A	1.13 ± 0.35
**Flavones**	
Pebrellin	1.62 ± 0.29
Cirsilineol	1.62 ± 0.29
Geraldone	1.49 ± 0.78
Apigenin 6,8-C-arabinoside-C-glucoside	1.39 ± 0.21
Apigenin 6,8-C-galactoside-C-arabinoside	1.39 ± 0.20

Compounds	VIP score
Apigenin 7-O-apiosyl-glucoside	1.39 ± 0.20
Chrysoeriol 7-O-(6''-malonyl-apiosyl-glucoside)	1.36 ± 0.31
Luteolin 7-O-diglucuronide	1.33 ± 0.27
Luteolin 7-O-(2-apiosyl-glucoside)	1.32 ± 0.22
Chrysoeriol 7-O-(6''-malonyl-glucoside)	1.30 ± 0.26
6-Hydroxyluteolin 7-O-rhamnoside	1.29 ± 0.40
Luteolin 6-C-glucoside	1.28 ± 0.39
Luteolin 7-O-malonyl-glucoside	1.27 ± 0.32
Jaceosidin	1.22 ± 0.27
Luteolin	1.22 ± 0.34
Apigenin	1.12 ± 0.62
Apigenin 7-O-diglucuronide	1.09 ± 0.18
Hispidulin	1.09 ± 0.22
Chrysoeriol 7-O-glucoside	1.07 ± 0.21
Apigenin 7-O-glucoside	1.06 ± 0.42
Luteolin 7-O-(2-apiosyl-6-malonyl)-glucoside	1.04 ± 0.40
**Flavanones**	
Eriodictyol 7-O-glucoside	1.52 ± 0.29
Naringenin 7-O-glucoside	1.35 ± 0.53
Naringin 4'-O-glucoside	1.07 ± 0.34
Hesperetin	1.06 ± 0.38
Naringin	1.01 ± 0.77
**Isoflavonoids**	
Glycitein	1.49 ± 0.78
6''-O-Malonylgenistin	1.16 ± 0.29
6''-O-Malonylglycitin	1.09 ± 0.17
**Flavonols**	
Quercetin 3-O-acetyl-rhamnoside	1.57 ± 0.37
Methylgalangin	1.49 ± 0.78
Myricetin 3-O-galactoside	1.49 ± 0.27
Myricetin 3-O-glucoside	1.46 ± 0.25
5,3',4'-Trihydroxy-3-methoxy-6:7-methylenedioxyflavone 4'-O-glucuronide	1.45 ± 0.24
Quercetin 4'-O-glucoside	1.40 ± 0.21
Myricetin 3-O-rhamnoside	1.40 ± 0.21
Quercetin 3-O-galactoside	1.38 ± 0.25
Quercetin 3-O-glucoside	1.36 ± 0.22
Quercetin 3-O-glucosyl-xyloside	1.32 ± 0.22
Kaempferol 3-O-xylosyl-glucoside	1.29 ± 0.40
Quercetin 3-O-rhamnoside	1.29 ± 0.40
Kaempferol 3-O-galactoside	1.27 ± 0.32
Kaempferol	1.27 ± 0.32
Kaempferol 7-O-glucoside	1.26 ± 0.48
Quercetin 3-O-xylosyl-glucuronide	1.26 ± 0.35
Isorhamnetin 7-O-rhamnoside	1.25 ± 0.35
Isorhamnetin 3-O-glucoside	1.24 ± 0.34
Myricetin	1.23 ± 0.34
3,7-Dimethylquercetin	1.22 ± 0.28
Kaempferol	1.22 ± 0.34
Isorhamnetin 3-O-glucuronide	1.21 ± 0.28
Kaempferol 3-O-glucosyl-rhamnosyl-glucoside	1.13 ± 0.38
Quercetin 3-O-xylosyl-rutinoside	1.12 ± 0.15
Myricetin 3-O-arabinoside	1.10 ± 0.62
Kaempferol 3-O-glucoside	1.08 ± 0.40
Isorhamnetin 3-O-rutinoside	1.07 ± 0.21
Isorhamnetin 3-O-glucoside 7-O-rhamnoside	1.05 ± 0.23
Quercetin 3,4'-O-diglucoside	1.04 ± 0.20

Compounds	VIP score
Quercetin 7,4'-O-diglucoside	1.03 ± 0.20
**Anthocyanins**	
Peonidin 3-O-(6''-acetyl-galactoside)	1.63 ± 0.17
Peonidin 3-O-(6''-acetyl-glucoside)	1.55 ± 0.20
Malvidin 3-O-(6''-acetyl-glucoside)	1.40 ± 0.38
Pelargonidin 3-O-sambubioside	1.39 ± 0.19
Pelargonidin 3,5-O-diglucoside	1.39 ± 0.19
Delphinidin 3-O-(6''-acetyl-galactoside)	1.33 ± 0.50
Cyanidin 3-O-(6''-acetyl-glucoside)	1.33 ± 0.36
Malvidin 3,5-O-diglucoside	1.32 ± 0.18
Pelargonidin 3-O-(6''-malonyl-glucoside)	1.31 ± 0.22
Petunidin 3-O-(6''-p-coumaroyl-glucoside)	1.31 ± 0.16
Cyanidin	1.30 ± 0.43
Petunidin 3-O-glucoside	1.28 ± 0.30
Cyanidin 3-O-sambubioside	1.28 ± 0.33
Cyanidin 3-O-xylosyl-rutinoside	1.26 ± 0.20
Cyanidin 3-O-(6''-succinyl-glucoside)	1.26 ± 0.45
Petunidin 3-O-galactoside	1.25 ± 0.28
Malvidin 3-O-(6''-acetyl-galactoside)	1.24 ± 0.49
Malvidin 3-O-glucoside	1.22 ± 0.11
Delphinidin 3-O-sambubioside	1.21 ± 0.42
Cyanidin 3-O-(6''-acetyl-galactoside)	1.20 ± 0.28
Cyanidin 3-O-(6''-malonyl-glucoside)	1.20 ± 0.50
Malvidin 3-O-arabinoside	1.19 ± 0.14
Peonidin 3-O-glucoside	1.18 ± 0.14
Petunidin 3-O-rhamnoside	1.17 ± 0.42
Delphinidin 3-O-glucoside	1.16 ± 0.41
Vitisin A	1.08 ± 0.50
Pelargonidin 3-O-glucoside	1.08 ± 0.20
Malvidin 3-O-galactoside	1.07 ± 0.33
Cyanidin 3-O-xyloside	1.05 ± 0.35
Pelargonidin 3-O-rutinoside	1.05 ± 0.18
Pelargonidin 3-O-(6''-succinyl-glucoside)	1.02 ± 0.11
Petunidin 3,5-O-diglucoside	1.01 ± 0.17
**Flavanols**	
Theaflavin	1.40 ± 0.21
Procyanidin dimer B2	1.39 ± 0.22
(-)-Epigallocatechin	1.35 ± 0.40
Prodelphinidin dimer B3	1.17 ± 0.41
(+)-Catechin 3-O-glucose	1.12 ± 0.39
(+)-Gallocatechin 3-O-gallate	1.10 ± 0.32
Procyanidin dimer B7	1.08 ± 0.33

Table S4. Metabolites with the highest contribution to discrimination between organs: aerial parts and roots, according by the OPLS-DA predictive model, followed by VIP selection method. Metabolites are grouped into their subfamilies and accompanied by their VIP score and standard error.

Compounds	VIP score
**LMW**	
Pyrogallol	1.78 ± 0.38
2,3-Dihydroxy-1-guaiacylpropanone	1.76 ± 0.37
4-Methylcatechol	1.70 ± 0.48
Phloretin	1.58 ± 0.64
Eugenol	1.49 ± 0.53
Thymol	1.43 ± 0.41
4-Vinylguaiacol	1.42 ± 0.31
Juglone	1.41 ± 0.23
4-Vinylphenol	1.38 ± 0.46
Umbelliferone	1.36 ± 0.15
Esculetin	1.30 ± 0.45
Anethole	1.29 ± 0.48
24-Methylcholesterol ferulate	1.25 ± 0.52
p-Coumaroyl tyrosine	1.23 ± 0.28
Carvacrol	1.20 ± 0.32
Ligstroside	1.18 ± 0.15
Esculin	1.11 ± 0.68
Carnosic acid	1.09 ± 0.22
4-Ethylphenol	1.07 ± 0.67
Oleuropein	1.07 ± 0.65
Bisdemethoxycurcumin	1.02 ± 0.59
Oleoside 11-methylester	1.00 ± 0.48
**Phenolic acids**	
Hydroxycaffeic acid	1.64 ± 0.45
Methoxyphenylacetic acid	1.56 ± 0.39
Ellagic acid acetyl-arabinoside	1.53 ± 0.15
Cinnamoyl glucose	1.46 ± 0.38
24-Methylcholestanol ferulate	1.46 ± 0.61
Verbascoside	1.45 ± 0.24
24-Methyllathosterol ferulate	1.45 ± 0.36
1,2,2'-Trisinapoylgentiobiose	1.44 ± 0.35
Valoneic acid dilactone	1.43 ± 0.38
Dihydro-p-coumaric acid	1.42 ± 0.38
2-S-Glutathionyl caftaric acid	1.41 ± 0.46
Gentisic acid	1.36 ± 0.63
Ellagic acid acetyl-xyloside	1.35 ± 0.19
Cinnamic acid	1.34 ± 0.44
Avenanthramide K	1.32 ± 0.25
Caffeic acid	1.26 ± 0.24
1-Sinapoyl-2,2'-diferuloylgentiobiose	1.21 ± 0.42
Protocatechuic acid 4-O-glucoside	1.20 ± 0.58
p-Coumaric acid ethyl ester	1.17 ± 0.56
Protocatechuic aldehyde	1.16 ± 0.30
2,5-di-S-Glutathionyl caftaric acid	1.15 ± 0.38
8-O-4'-Dehydrodiferulic acid	1.13 ± 0.58
Chicoric acid	1.09 ± 0.49
**Lignans**	
Medioresinol	1.64 ± 0.47
1-Acetoxypinoresinol	1.47 ± 0.44
Syringaresinol	1.29 ± 0.55
Pinoresinol	1.25 ± 0.45

Compounds	VIP score
Sesaminol	1.20 ± 0.39
Sesamolin	1.18 ± 0.37
5-Pentadecylresorcinol	1.06 ± 0.46
Isohydroxymatairesinol	1.02 ± 0.75
**Stilbenes**	
Piceatannol	1.50 ± 0.69
Pinosylvin	1.31 ± 0.59
Vitisin A	1.13 ± 0.53
Piceatannol 3-O-glucoside	1.05 ± 0.46
**Flavones**	
Luteolin 7-O-diglucuronide	1.65 ± 0.26
Apigenin 7-O-diglucuronide	1.63 ± 0.31
Apigenin 6,8-C-arabinoside-C-glucoside	1.49 ± 0.28
Apigenin 6,8-C-galactoside-C-arabinoside	1.49 ± 0.27
Jaceosidin	1.45 ± 0.55
Chrysoeriol 7-O-glucoside	1.38 ± 0.31
Chrysoeriol 7-O-(6''-malonyl-glucoside)	1.35 ± 0.52
Daidzein/Chrysin	1.32 ± 0.42
Xanthohumol	1.29 ± 0.33
Chrysoeriol 7-O-(6''-malonyl-apiosyl-glucoside)	1.20 ± 0.63
Phloridzin	1.17 ± 0.68
6-Hydroxyluteolin 7-O-rhamnoside	1.13 ± 0.35
Luteolin 7-O-glucoside	1.11 ± 0.36
Hispidulin	1.08 ± 0.36
**Flavanones**	
6-Prenylnaringenin	1.65 ± 0.37
Neoeriocitrin	1.50 ± 0.37
Eriocitrin	1.50 ± 0.37
Naringenin 7-O-glucoside	1.17 ± 0.64
Naringin 4'-O-glucoside	1.09 ± 0.49
**Isoflavonoids**	
6''-O-Malonyldaidzin	1.56 ± 0.36
6''-O-Malonylglycitin	1.53 ± 0.38
Daidzin	1.24 ± 0.66
**Flavonols**	
Isorhamnetin 3-O-glucoside 7-O-rhamnoside	1.95 ± 0.20
Quercetin 3-O-xylosyl-glucuronide	1.84 ± 0.26
Quercetin 7,4'-O-diglucoside	1.56 ± 0.27
Quercetin 3,4'-O-diglucoside	1.56 ± 0.27
Quercetin 3-O-(6''-acetyl-galactoside) 7-O-rhamnoside	1.55 ± 0.22
Quercetin 3-O-glucuronide	1.53 ± 0.40
5,3',4'-Trihydroxy-3-methoxy-6:7-methylenedioxyflavone 4'-O-glucuronide	1.52 ± 0.26
Kaempferol 3-O-sophoroside 7-O-glucoside	1.44 ± 0.09
Quercetin 3-O-glucosyl-rhamnosyl-glucoside	1.44 ± 0.09
Kaempferol 3-O-rhamnoside	1.44 ± 0.49
Kaempferol	1.42 ± 0.43
Isorhamnetin 3-O-galactoside	1.37 ± 0.56
Quercetin 3-O-glucoside	1.35 ± 0.38
Dihydroquercetin	1.35 ± 0.43
Quercetin 3-O-galactoside	1.31 ± 0.37
6,8-Dihydroxykaempferol	1.29 ± 0.45
Isorhamnetin 7-O-rhamnoside	1.28 ± 0.44
Spinacetin 3-O-glucosyl-(1-6)-glucoside	1.25 ± 0.22
Quercetin 3-O-xylosyl-rutinoside	1.20 ± 0.44
Quercetin 3-O-rhamnoside	1.13 ± 0.35
Kaempferol 3-O-galactoside	1.11 ± 0.36

Compounds	VIP score
Quercetin 3-O-glucosyl-xyloside	1.11 ± 0.68
Myricetin 3-O-galactoside	1.09 ± 0.31
Morin	1.07 ± 0.71
Myricetin 3-O-glucoside	1.06 ± 0.30
Dihydromyricetin 3-O-rhamnoside	1.04 ± 0.43
Kaempferol 3-O-glucoside	1.04 ± 0.30
Quercetin 3-O-xyloside	1.04 ± 0.55
**Anthocyanins**	
Petunidin 3,5-O-diglucoside	1.87 ± 0.36
Cyanidin 3-O-galactoside	1.66 ± 0.33
Peonidin 3-O-(6''-acetyl-galactoside)	1.66 ± 0.16
Malvidin 3-O-glucoside	1.62 ± 0.34
Malvidin 3-O-arabinoside	1.59 ± 0.42
Cyanidin 3-O-sambubioside	1.49 ± 0.33
Cyanidin 3-O-(6''-malonyl-3''-glucosyl-glucoside)	1.49 ± 0.21
Cyanidin 3-O-xylosyl-rutinoside	1.48 ± 0.31
Pinotin A	1.47 ± 0.27
Delphinidin 3-O-(6''-acetyl-galactoside)	1.44 ± 0.53
Pelargonidin 3,5-O-diglucoside	1.43 ± 0.25
Malvidin 3,5-O-diglucoside	1.41 ± 0.24
Peonidin 3-O-(6''-acetyl-glucoside)	1.37 ± 0.24
Pelargonidin 3-O-sambubioside	1.34 ± 0.42
Petunidin 3-O-galactoside	1.32 ± 0.32
Cyanidin 3-O-xyloside	1.29 ± 0.74
Pelargonidin 3-O-(6''-malonyl-glucoside)	1.24 ± 0.26
Delphinidin 3-O-xyloside	1.24 ± 0.43
Delphinidin 3-O-arabinoside	1.24 ± 0.43
Cyanidin	1.21 ± 0.92
Malvidin 3-O-galactoside	1.19 ± 0.49
Pelargonidin 3-O-rutinoside	1.18 ± 0.26
Cyanidin 3-O-(6''-dioxalyl-glucoside)	1.17 ± 0.59
Peonidin 3-O-galactoside	1.12 ± 0.66
Cyanidin 3-O-sambubioside 5-O-glucoside	1.11 ± 0.24
Petunidin 3-O-rhamnoside	1.08 ± 0.57
Malvidin 3-O-(6''-p-coumaroyl-glucoside)	1.05 ± 0.53
Delphinidin 3-O-glucoside	1.03 ± 0.93
Cyanidin 3-O-(6''-succinyl-glucoside)	1.01 ± 0.51
Delphinidin 3-O-feruloyl-glucoside	1.00 ± 0.39
**Flavanols**	
Procyanidin dimer B2	1.58 ± 0.23
Procyanidin trimer C1	1.52 ± 0.29
Theaflavin 3-O-gallate	1.52 ± 0.22
Prodelphinidin trimer C-GC-C	1.52 ± 0.28
Prodelphinidin trimer GC-GC-C	1.46 ± 0.36
Theaflavin	1.45 ± 0.46
(+)-Catechin 3-O-gallate	1.24 ± 0.77
Procyanidin dimer B7	1.12 ± 0.39
(+)-Catechin 3-O-glucose	1.07 ± 0.55
