# Supplementary material for: The Combination of Untargeted Metabolomics and Machine Learning Predicts the Biosynthesis of Phenolic Compounds in Bryophyllum Medicinal Plants (Genus Kalanchoe)
Source: Plants (Basel). 2021 Nov 10;10(11):2430. doi: 10.3390/plants10112430 (PMC8620224; doi:10.3390/plants10112430)
Supplement: Supplementary file 1 [file plants-10-02430-s001.zip › Supplementary material_Figure S2-3.pdf]

Figure S2: representative chromatograms of leaf samples under the different test conditions;
Figure S3: representative chromatograms of root samples under the different test conditions;

Genotypes. BD: *B. daigremontianum*, BH: *B.* × *houghtonii*, BT: *B. tubiflorum*.

MS: Murashige and Skoog (1962) medium formulation; M: Macronutrients; m: micronutrients

BH Control

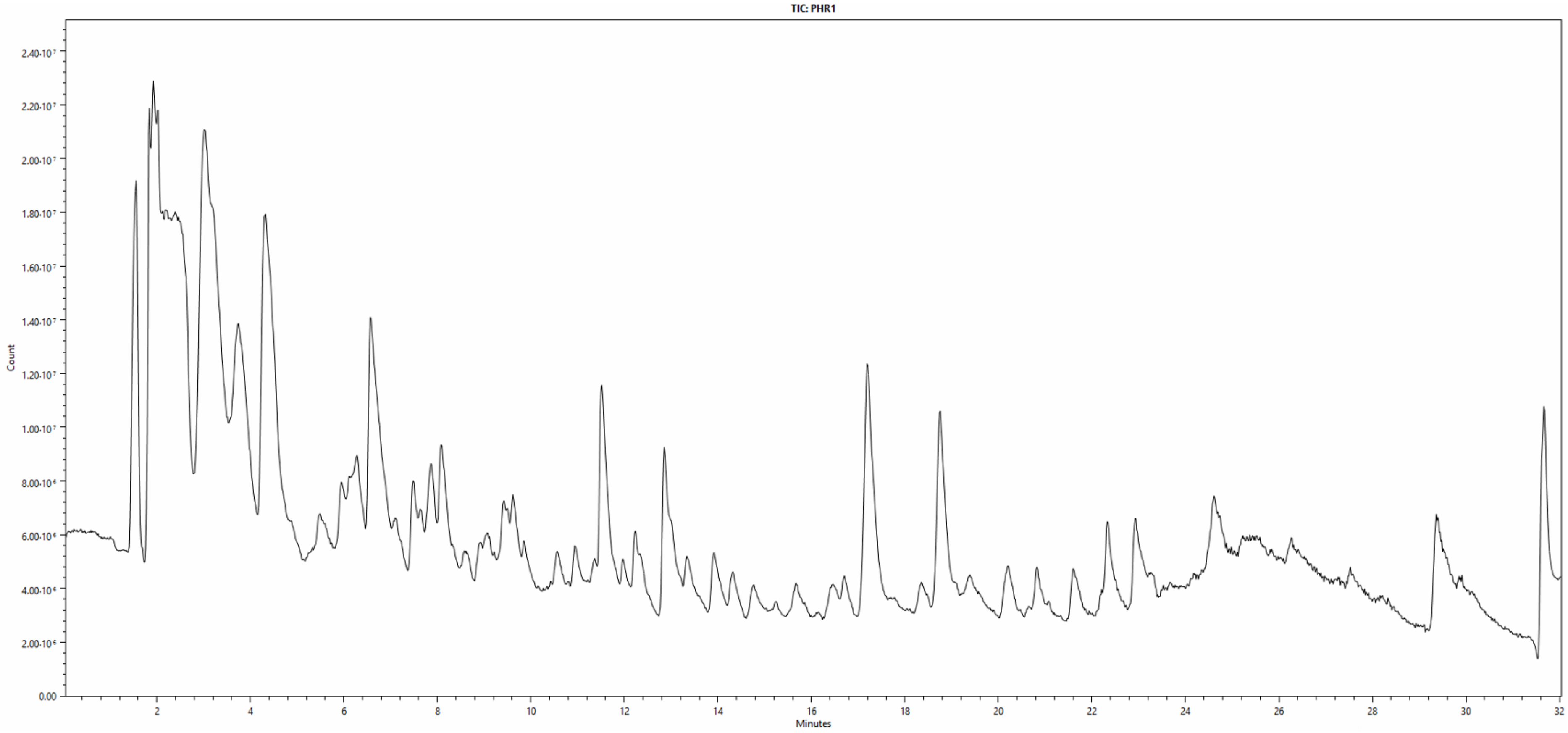

BH ½ MS M

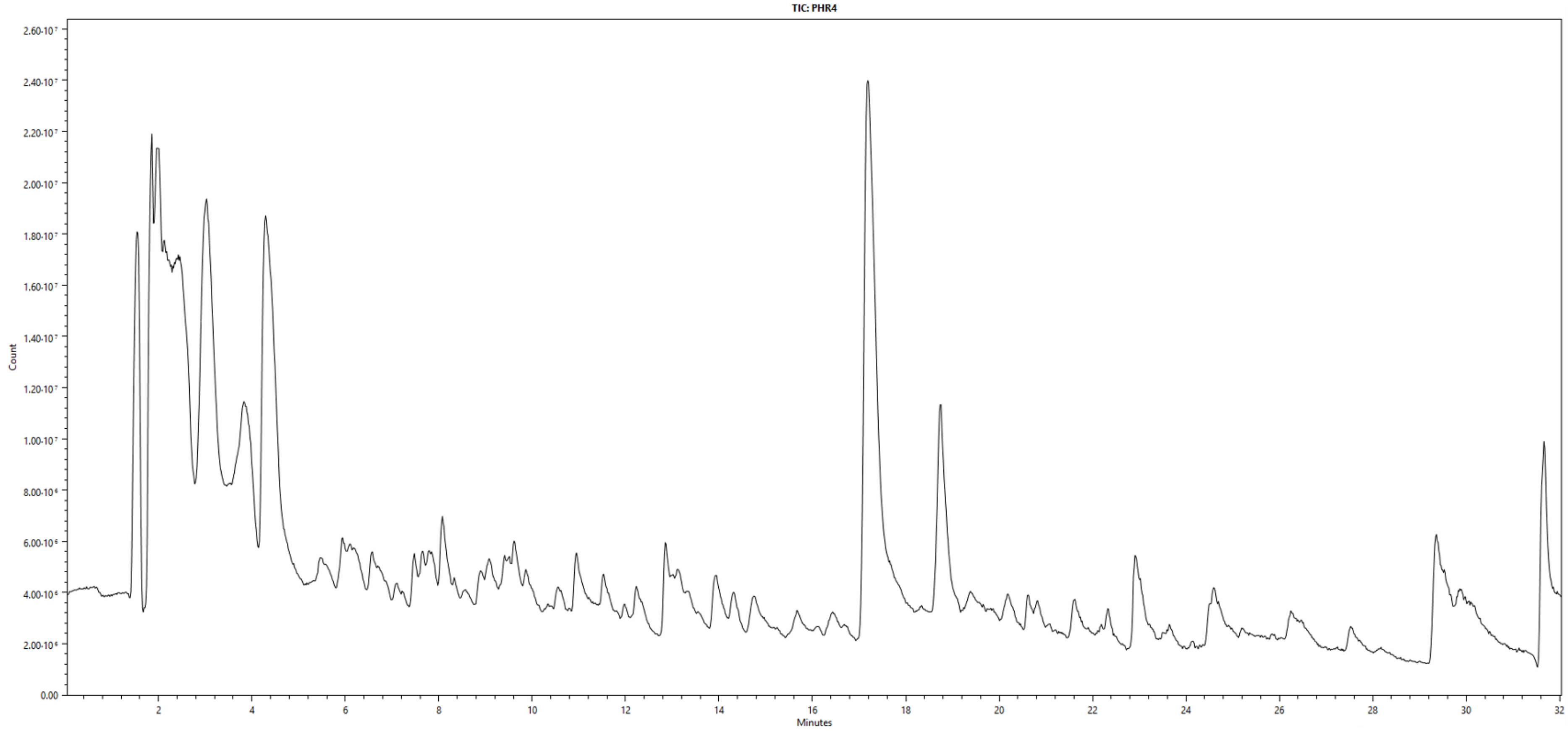

TIC: PHR4
Count
2.60·10⁷
2.40·10⁷
2.20·10⁷
2.00·10⁷
1.80·10⁷
1.60·10⁷
1.40·10⁷
1.20·10⁷
1.00·10⁷
8.00·10⁶
6.00·10⁶
4.00·10⁶
2.00·10⁶
0.00
2 4 6 8 10 12 14 16 18 20 22 24 26 28 30 32
Minutes

BH 1/4 MS M

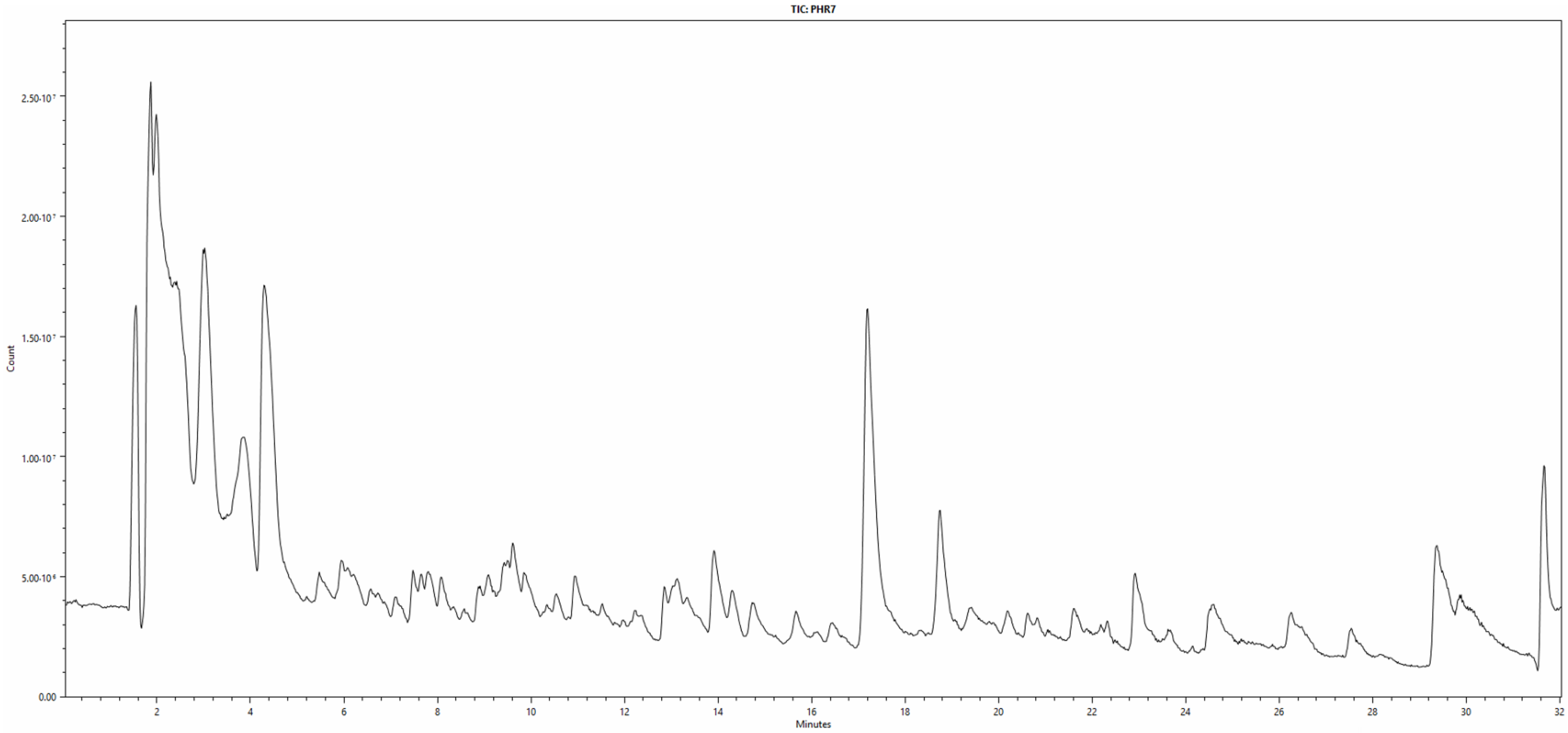

TIC: PHR7
Count
2.50·10⁷
2.00·10⁷
1.50·10⁷
1.00·10⁷
5.00·10⁶
0.00
2
4
6
8
10
12
14
16
18
20
22
24
26
28
30
32
Minutes

BH 1/8 MS M

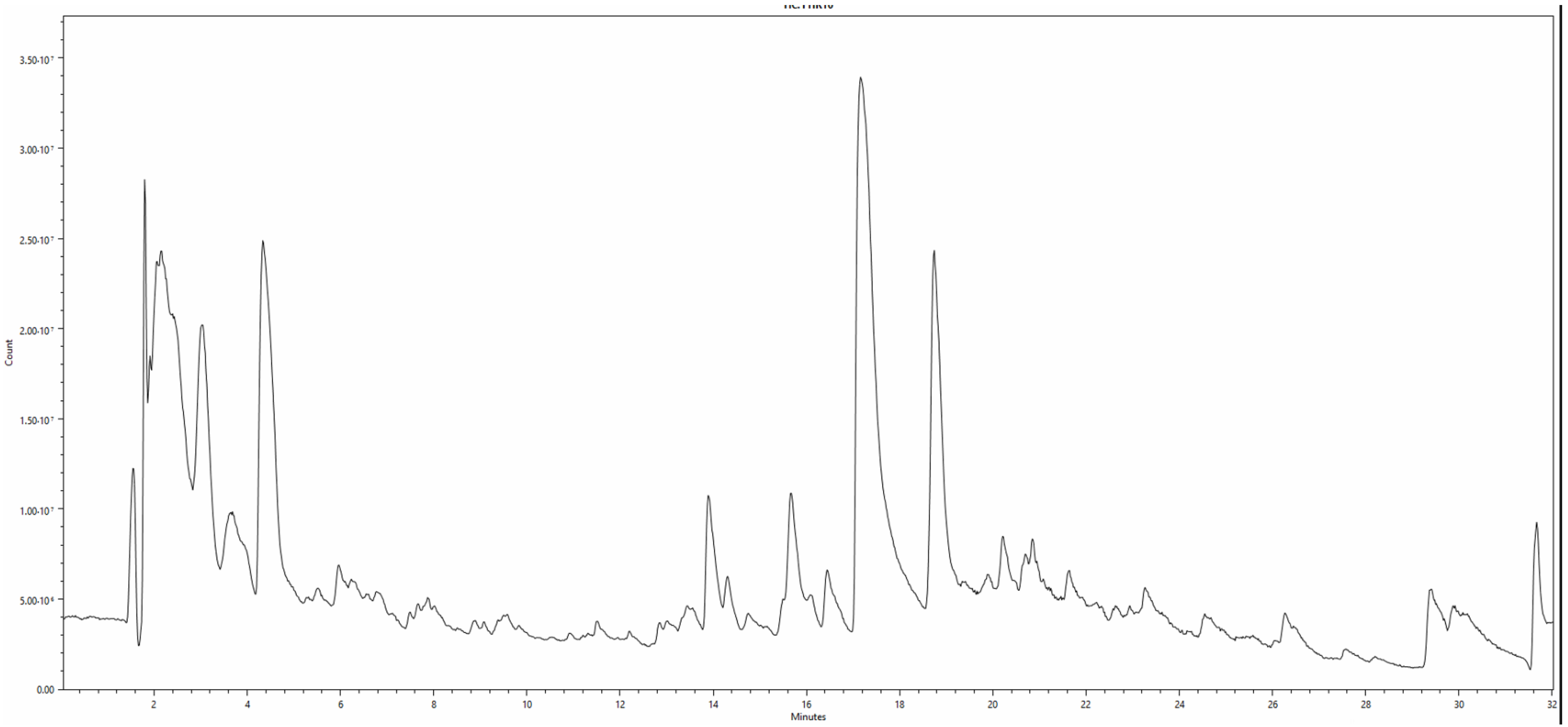

3.50·10⁷
3.00·10⁷
2.50·10⁷
2.00·10⁷
1.50·10⁷
1.00·10⁷
5.00·10⁶
0.00
Count
2
4
6
8
10
12
14
16
18
20
22
24
26
28
30
32
Minutes

BH ½ MS m

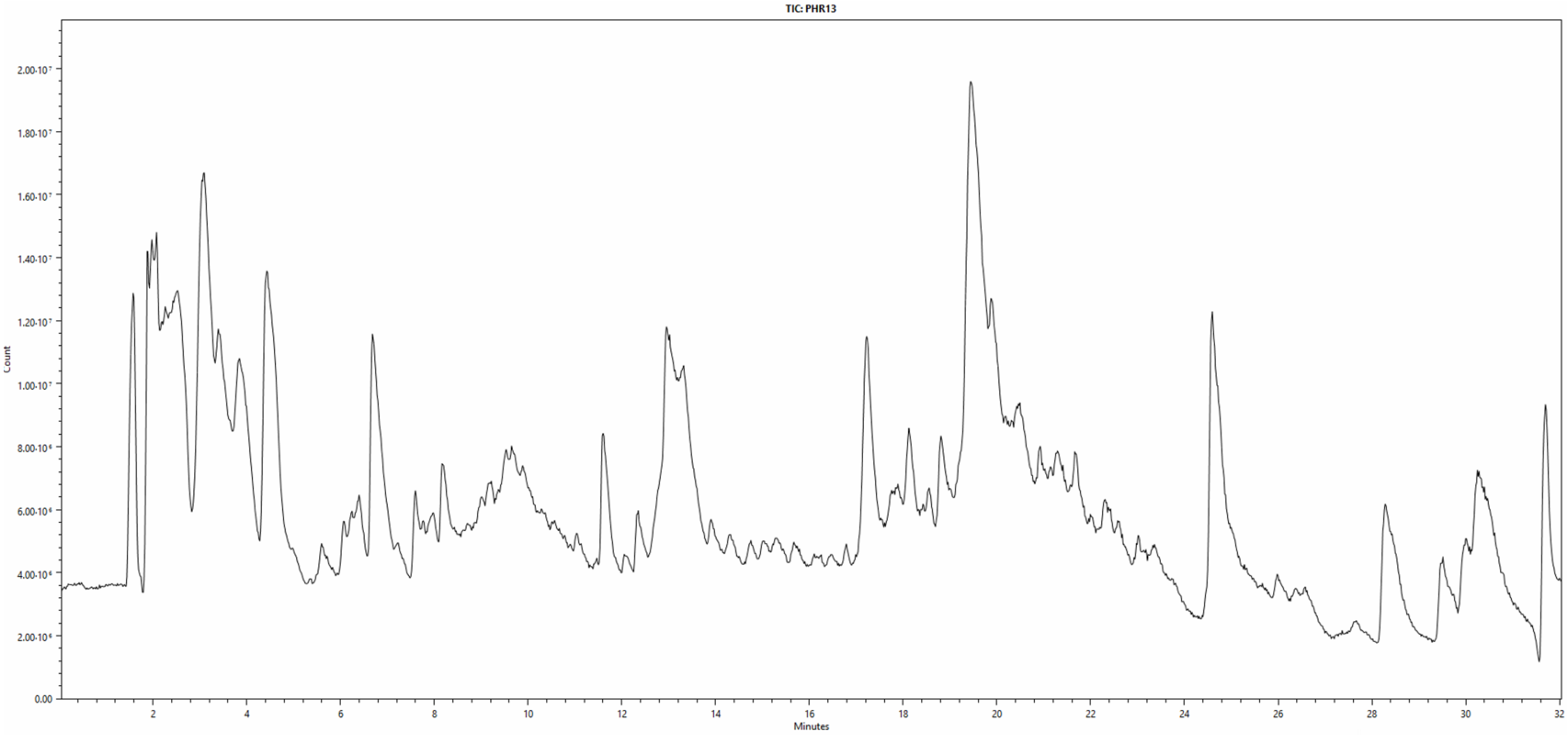

TIC: PHR13
Count
2.00·10⁷
1.80·10⁷
1.60·10⁷
1.40·10⁷
1.20·10⁷
1.00·10⁷
8.00·10⁶
6.00·10⁶
4.00·10⁶
2.00·10⁶
0.00
2
4
6
8
10
12
14
16
18
20
22
24
26
28
30
32
Minutes

BH 1/4 MS m

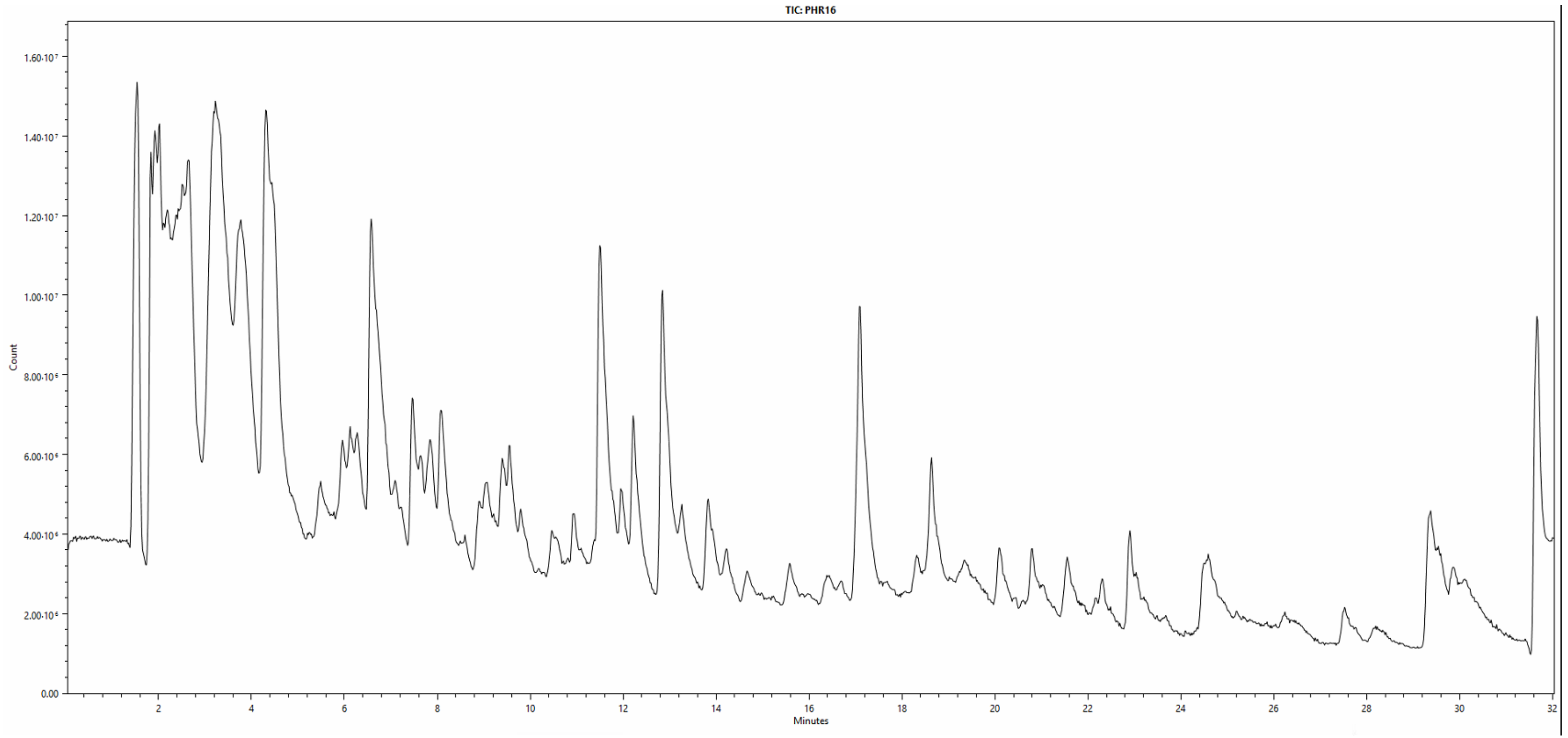
TIC: PHR16
Count
1.60·10⁷
1.40·10⁷
1.20·10⁷
1.00·10⁷
8.00·10⁶
6.00·10⁶
4.00·10⁶
2.00·10⁶
0.00
2
4
6
8
10
12
14
16
18
20
22
24
26
28
30
32
Minutes

BH 1/8 MS m

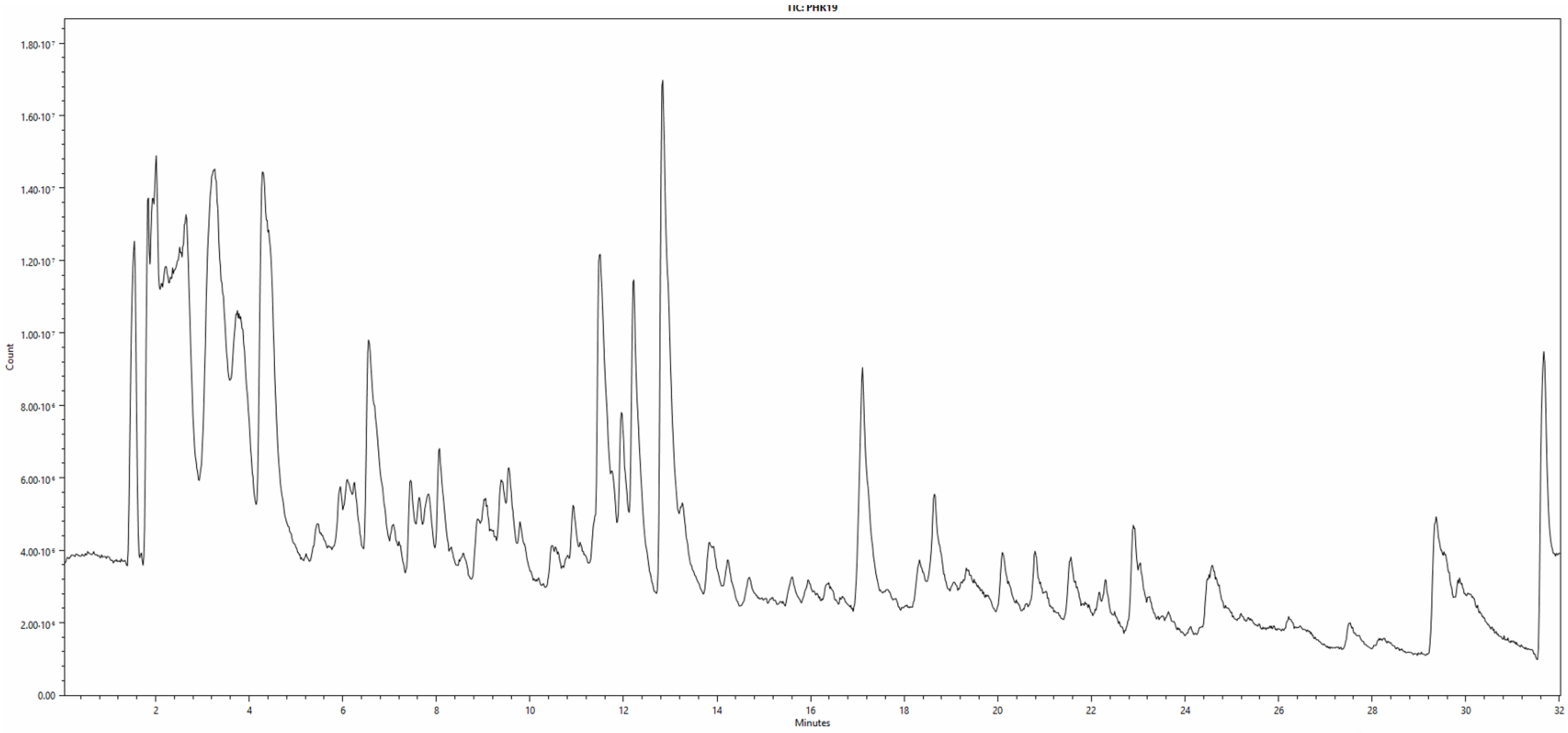

TIC: PHR19
Count
1.80·10⁷
1.60·10⁷
1.40·10⁷
1.20·10⁷
1.00·10⁷
8.00·10⁶
6.00·10⁶
4.00·10⁶
2.00·10⁶
0.00
2
4
6
8
10
12
14
16
18
20
22
24
26
28
30
32
Minutes

BD Control

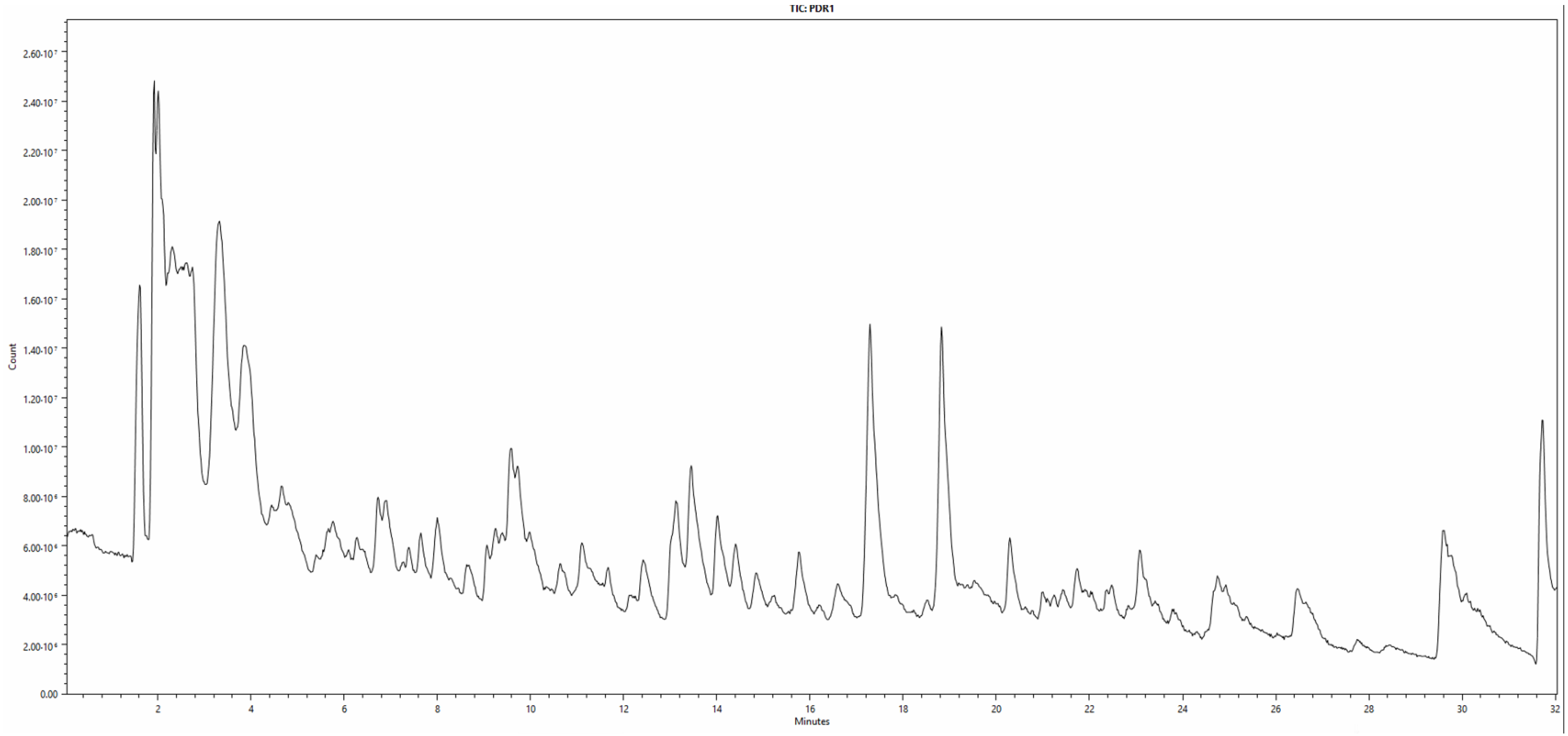

BD ½ MS M

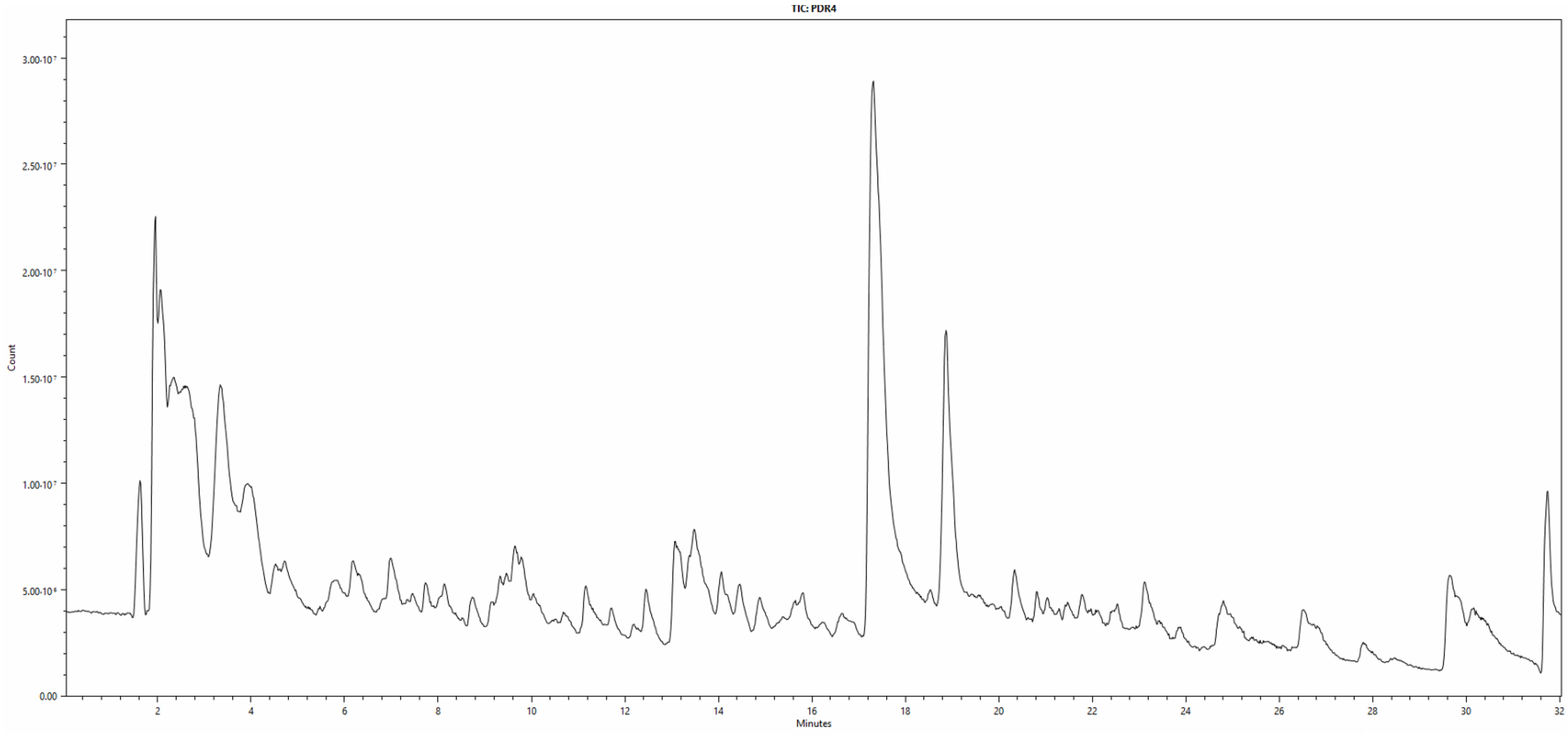
TIC: PDR4
Count
3.00·10⁷
2.50·10⁷
2.00·10⁷
1.50·10⁷
1.00·10⁷
5.00·10⁶
0.00
2
4
6
8
10
12
14
16
18
20
22
24
26
28
30
32
Minutes

BD 1/4 MS M

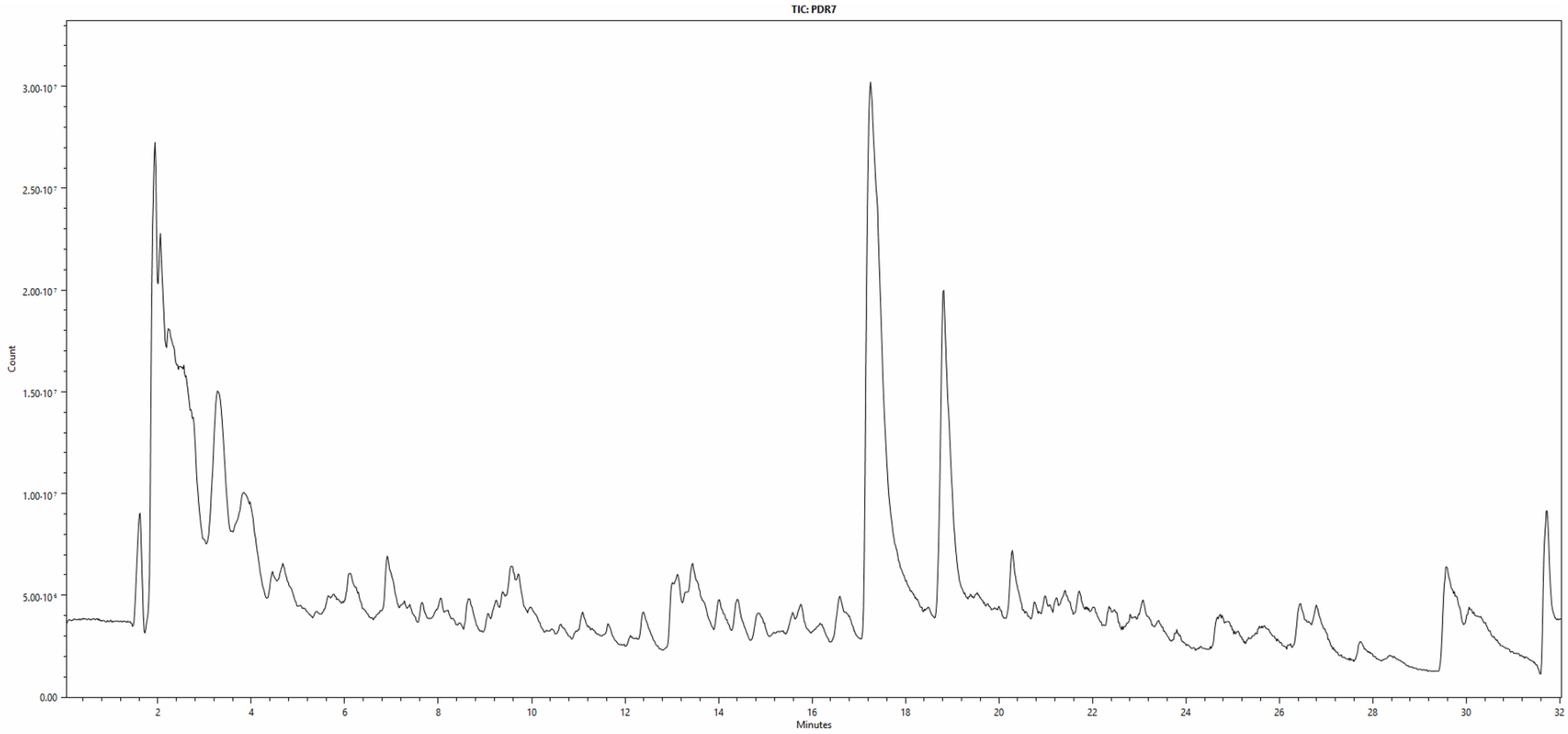

TIC: PDR7
Count
3.00·10⁷
2.50·10⁷
2.00·10⁷
1.50·10⁷
1.00·10⁷
5.00·10⁶
0.00
2
4
6
8
10
12
14
16
18
20
22
24
26
28
30
32
Minutes

BD 1/8 MS M

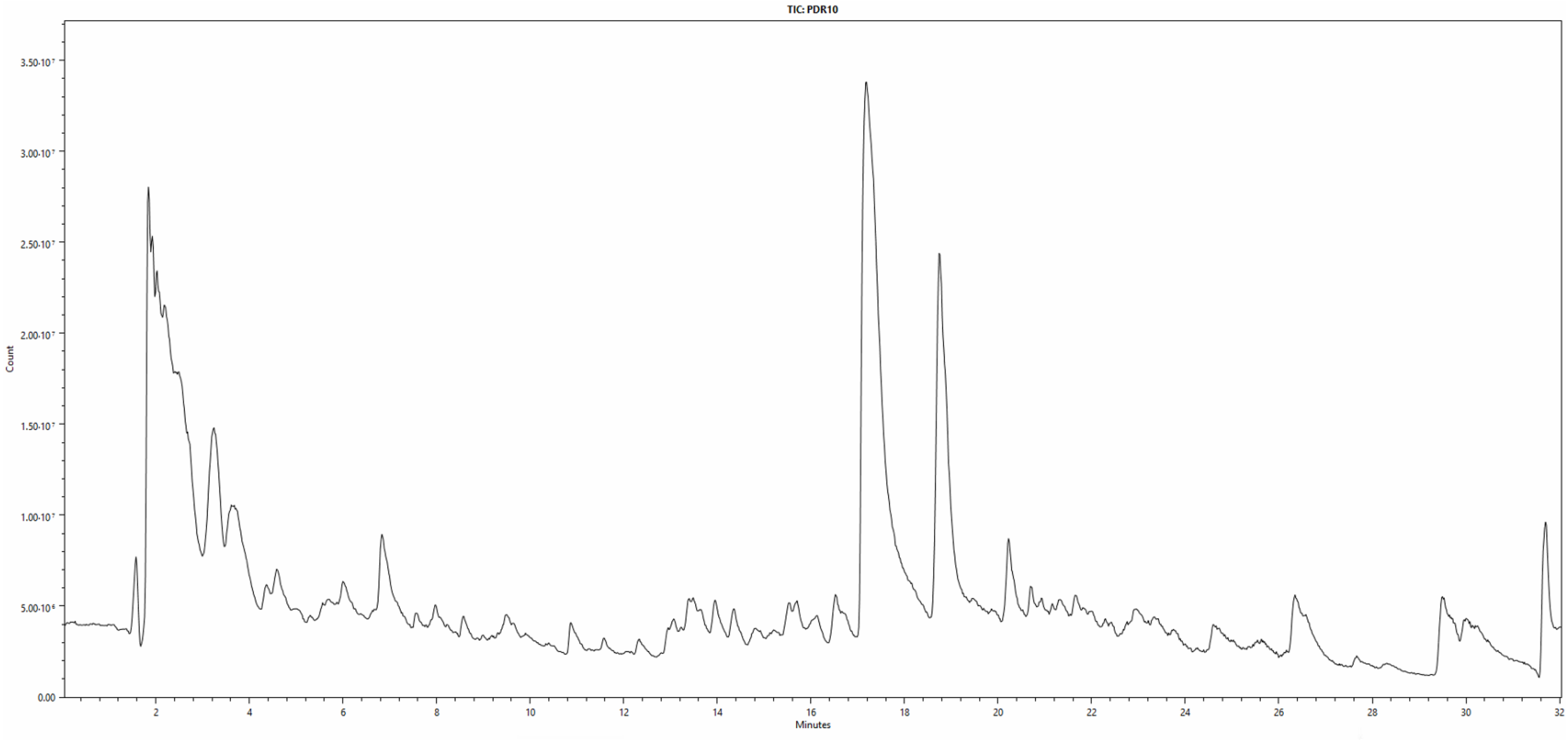

TIC: PDR10
3.50·10⁷
3.00·10⁷
2.50·10⁷
2.00·10⁷
1.50·10⁷
1.00·10⁷
5.00·10⁶
0.00
Count
2
4
6
8
10
12
14
16
18
20
22
24
26
28
30
32
Minutes

BD 1/2 MS m

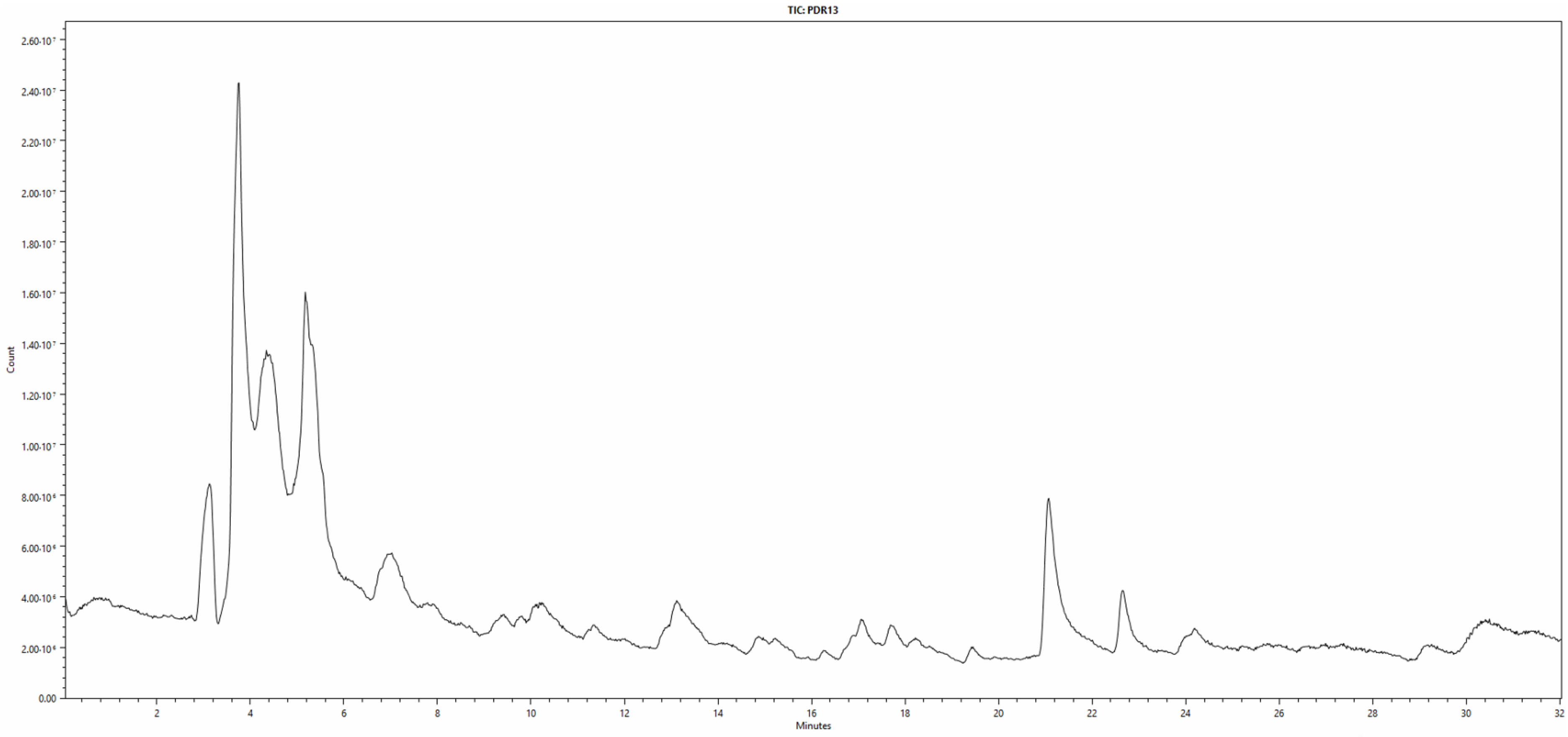
TIC: PDR13
Count
Minutes
2.60·10⁷
2.40·10⁷
2.20·10⁷
2.00·10⁷
1.80·10⁷
1.60·10⁷
1.40·10⁷
1.20·10⁷
1.00·10⁷
8.00·10⁶
6.00·10⁶
4.00·10⁶
2.00·10⁶
0.00
2
4
6
8
10
12
14
16
18
20
22
24
26
28
30
32

BD 1/4 MS m

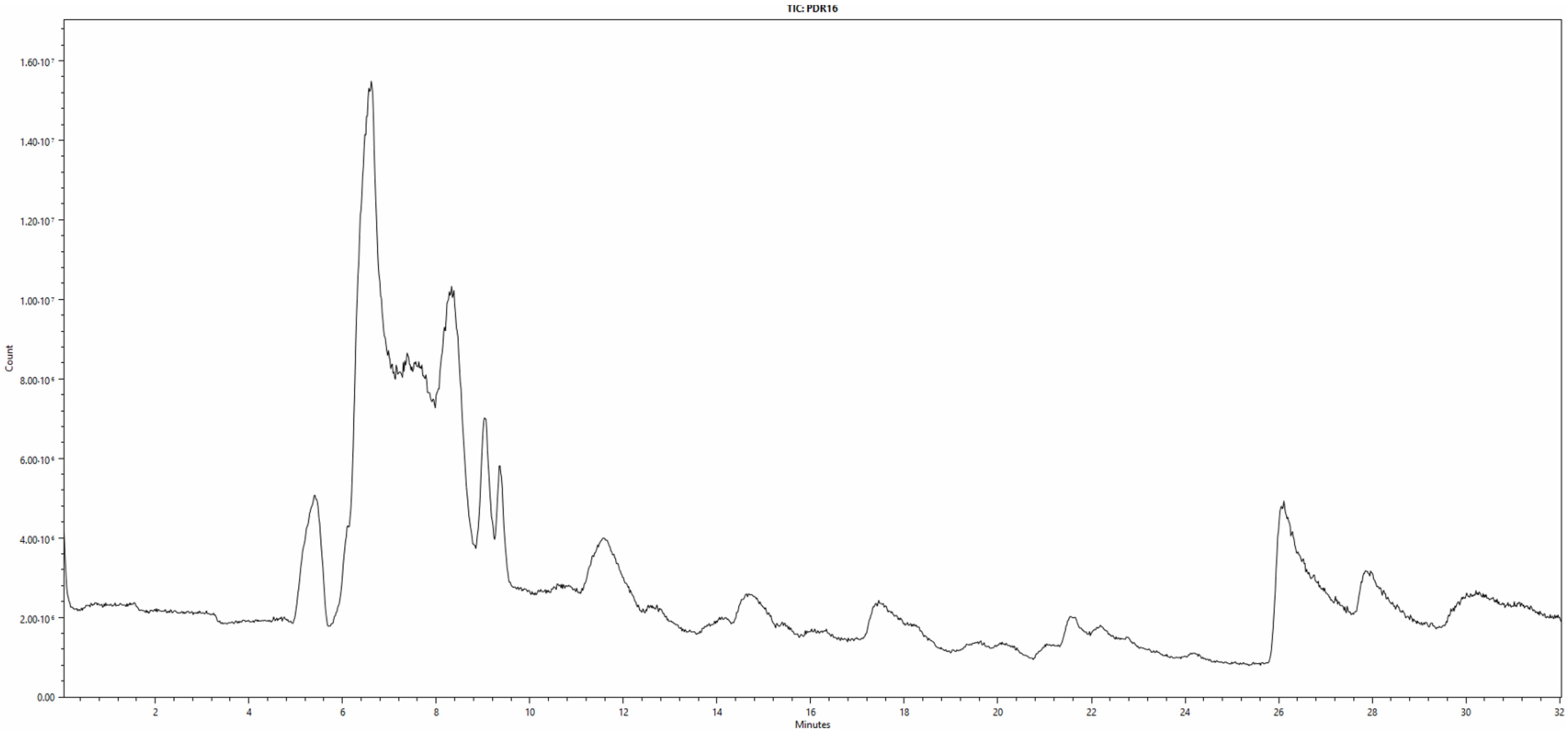
TIC: PDR16
Count
Minutes
1.60·10⁷
1.40·10⁷
1.20·10⁷
1.00·10⁷
8.00·10⁶
6.00·10⁶
4.00·10⁶
2.00·10⁶
0.00
2
4
6
8
10
12
14
16
18
20
22
24
26
28
30
32

BD 1/8 MS m

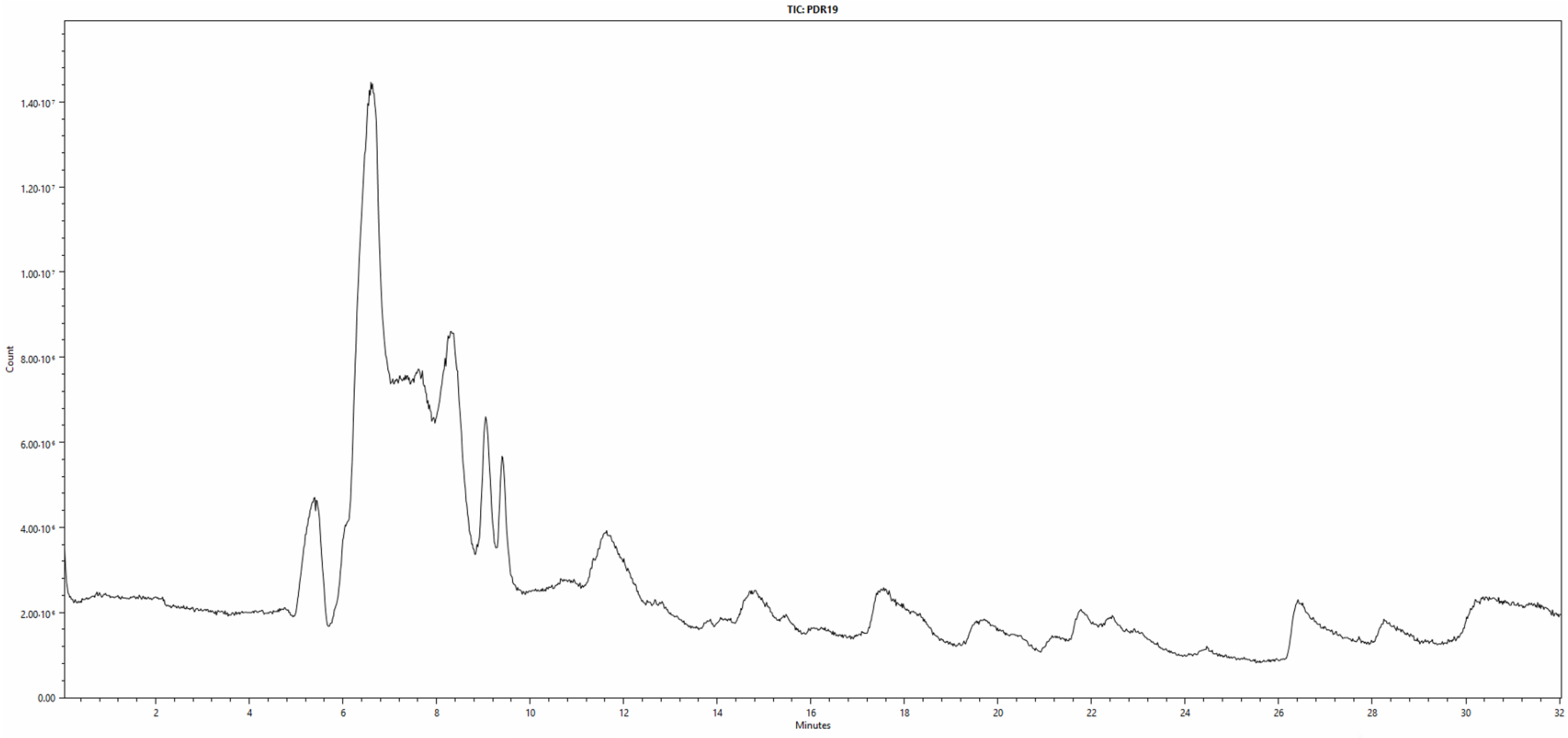

TIC: PDR19
Count
1.40·10⁷
1.20·10⁷
1.00·10⁷
8.00·10⁶
6.00·10⁶
4.00·10⁶
2.00·10⁶
0.00
2
4
6
8
10
12
14
16
18
20
22
24
26
28
30
32
Minutes

Xx

BD 1/2 MS m

BT Control

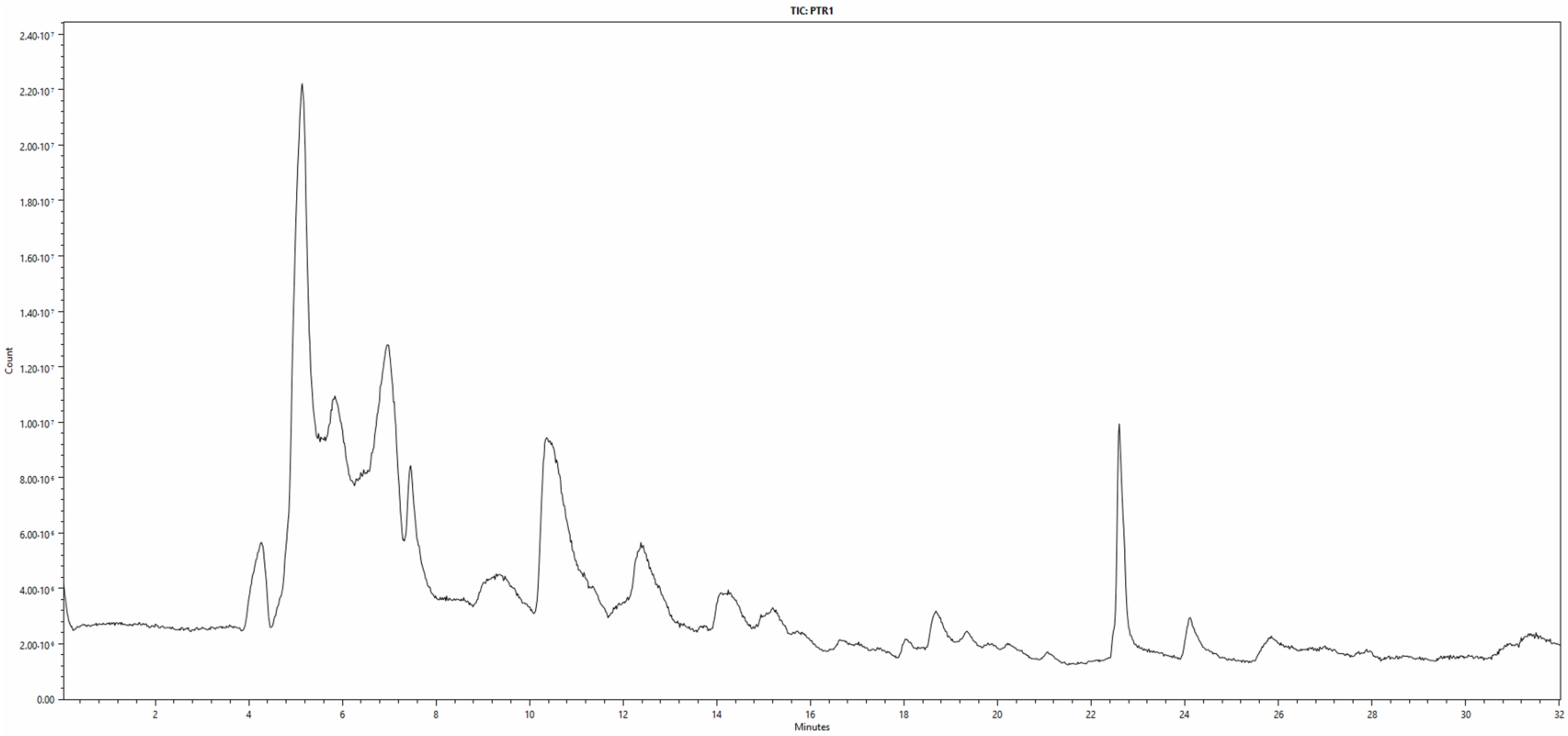

BT 1/2 MS M

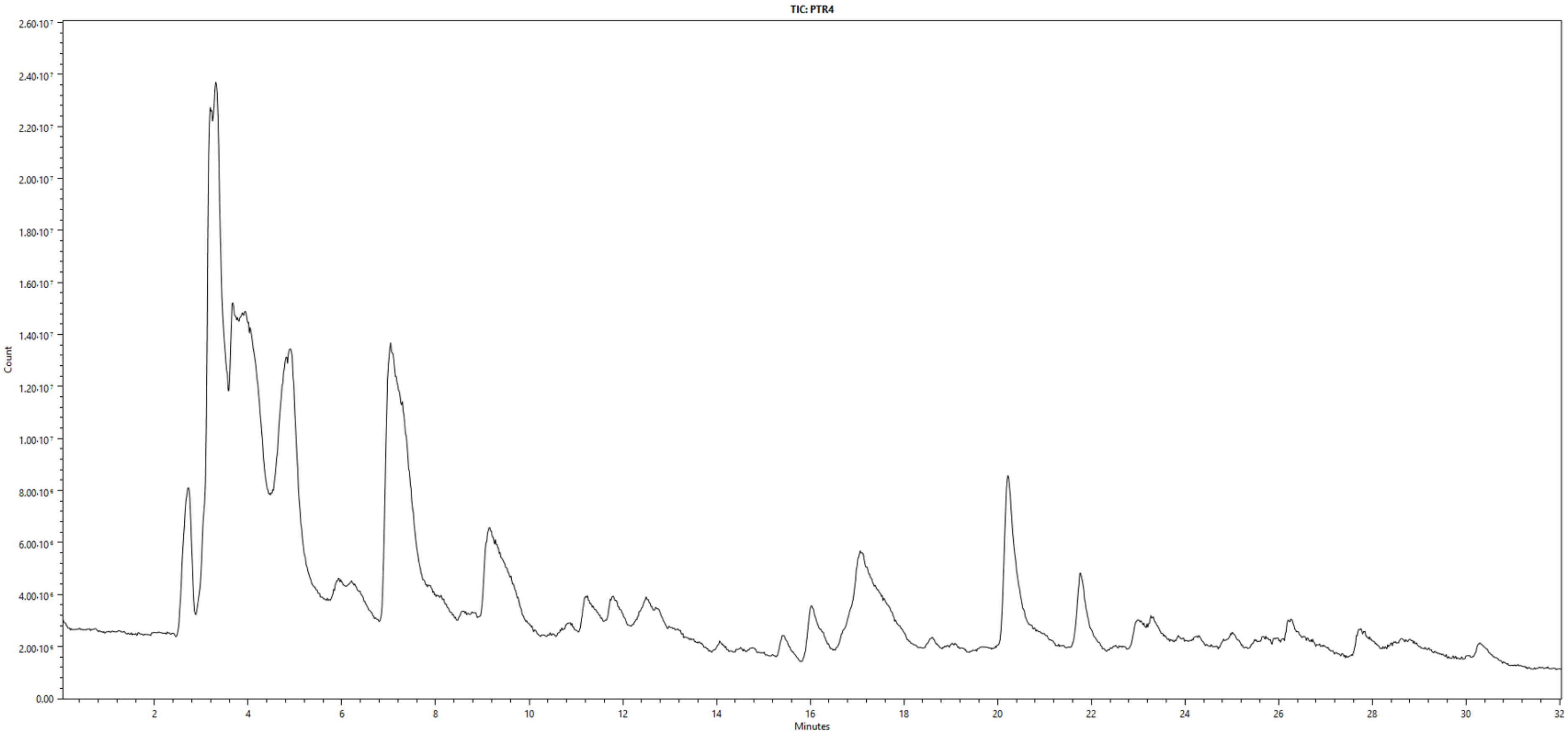
TIC: PTR4
Count
2.60·10⁷
2.40·10⁷
2.20·10⁷
2.00·10⁷
1.80·10⁷
1.60·10⁷
1.40·10⁷
1.20·10⁷
1.00·10⁷
8.00·10⁶
6.00·10⁶
4.00·10⁶
2.00·10⁶
0.00
2 4 6 8 10 12 14 16 18 20 22 24 26 28 30 32
Minutes

BT 1/4 MS M

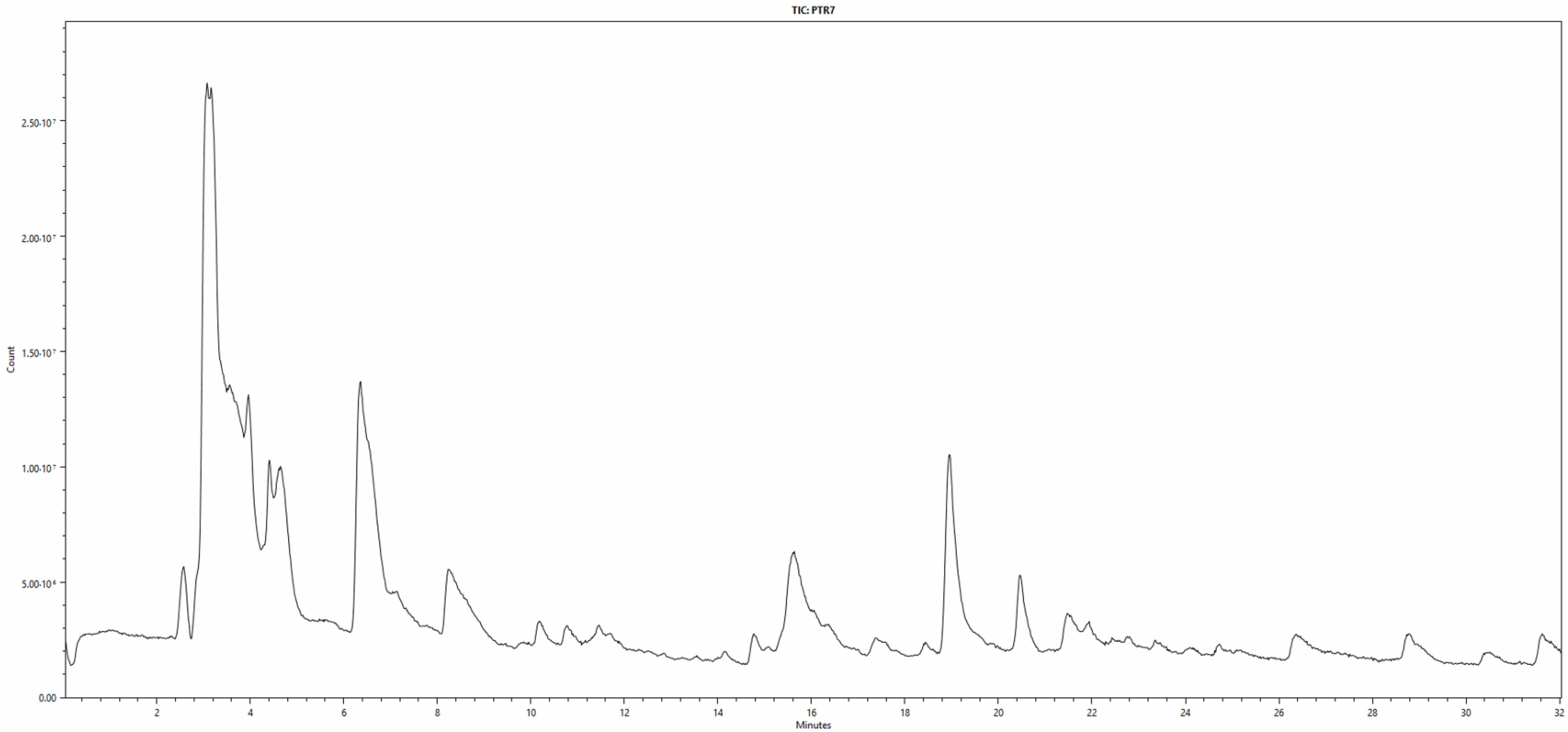
TIC: PTR7
Count
2.50·10⁷
2.00·10⁷
1.50·10⁷
1.00·10⁷
5.00·10⁶
0.00
2
4
6
8
10
12
14
16
18
20
22
24
26
28
30
32
Minutes

BT 1/8 MS M

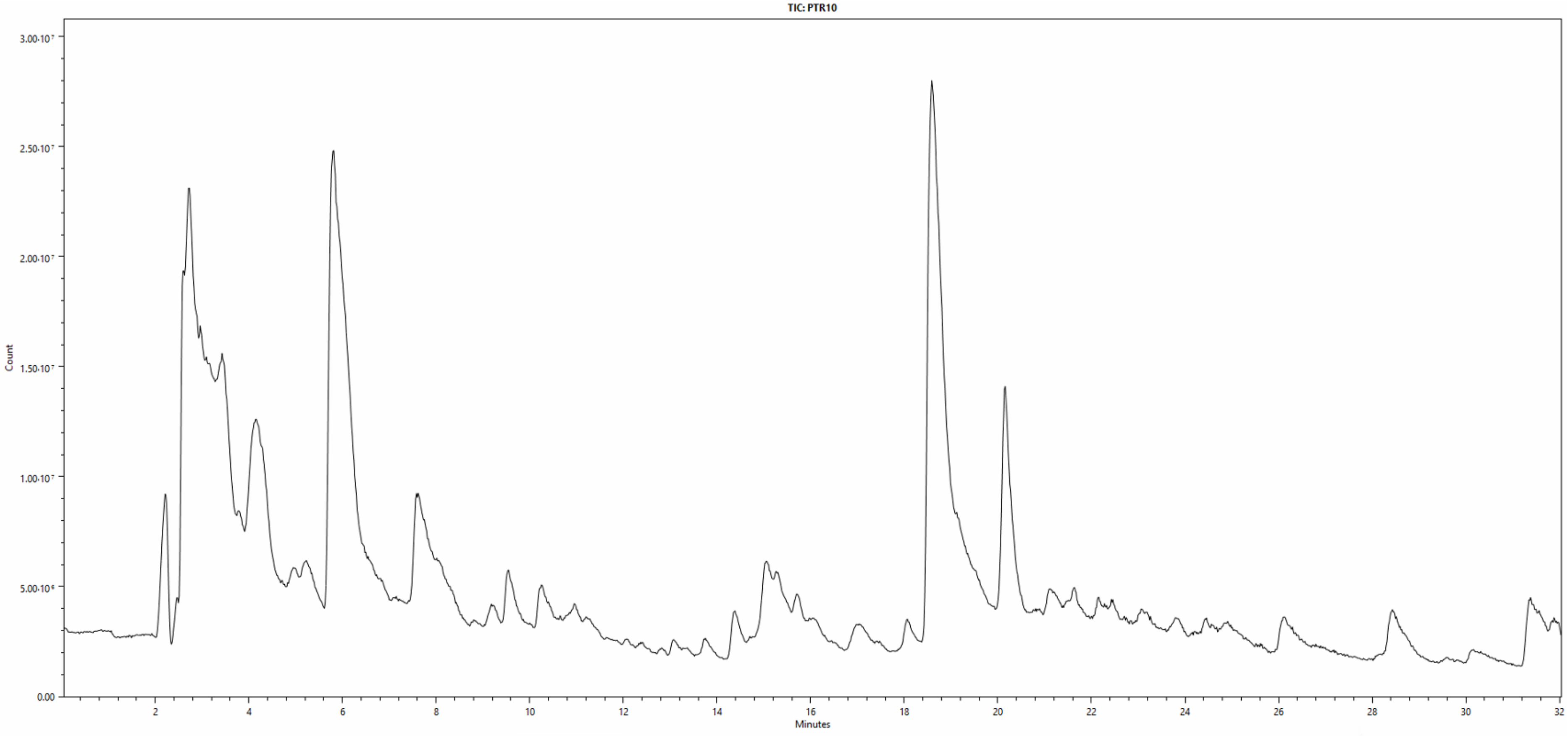

TIC: PTR10
Count
Minutes
3.00·10⁷
2.50·10⁷
2.00·10⁷
1.50·10⁷
1.00·10⁷
5.00·10⁶
0.00
2
4
6
8
10
12
14
16
18
20
22
24
26
28
30
32

BT 1/2 MS m

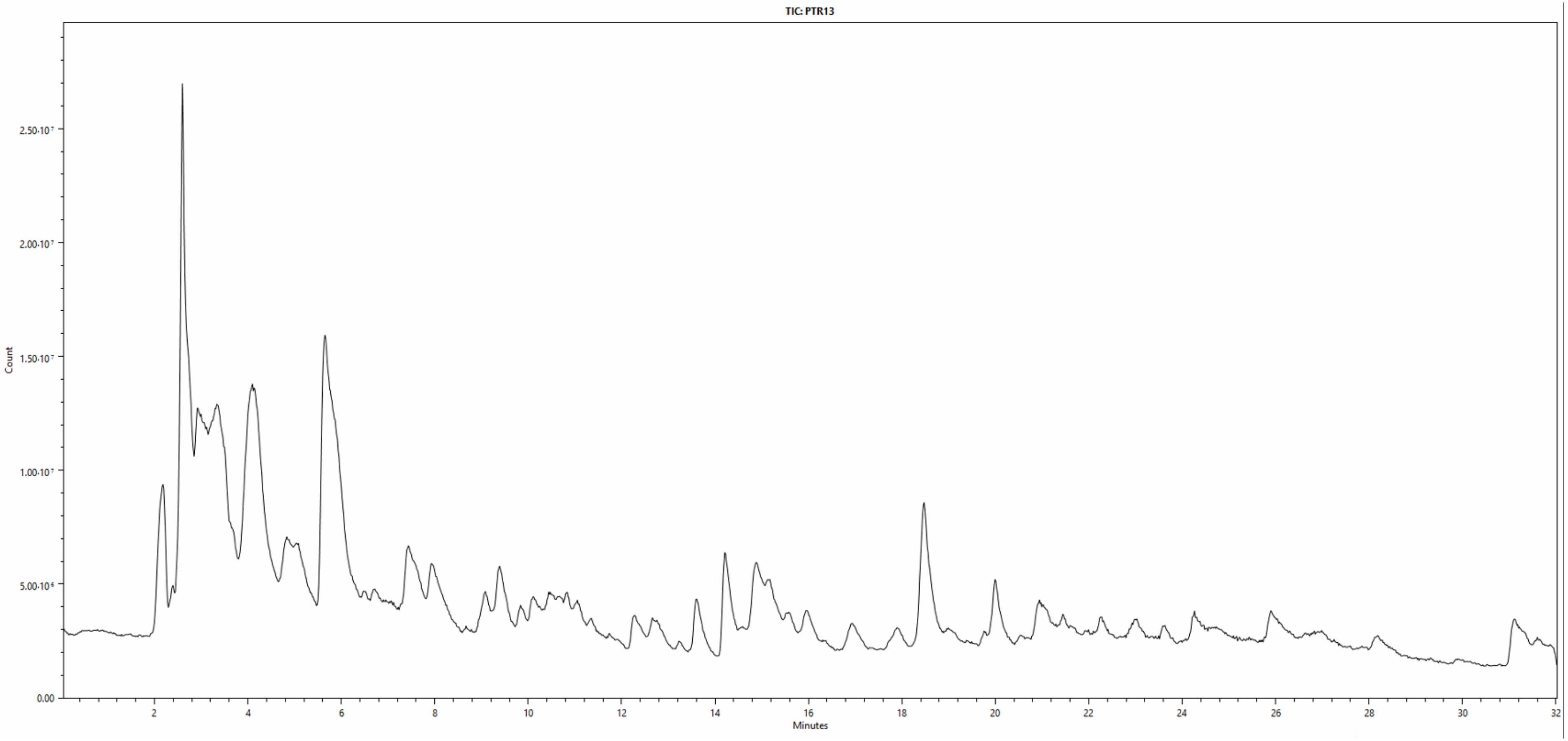
TIC: PTR13
Count
2.50·10⁷
2.00·10⁷
1.50·10⁷
1.00·10⁷
5.00·10⁶
0.00
2
4
6
8
10
12
14
16
18
20
22
24
26
28
30
32
Minutes

BT 1/4 MS m

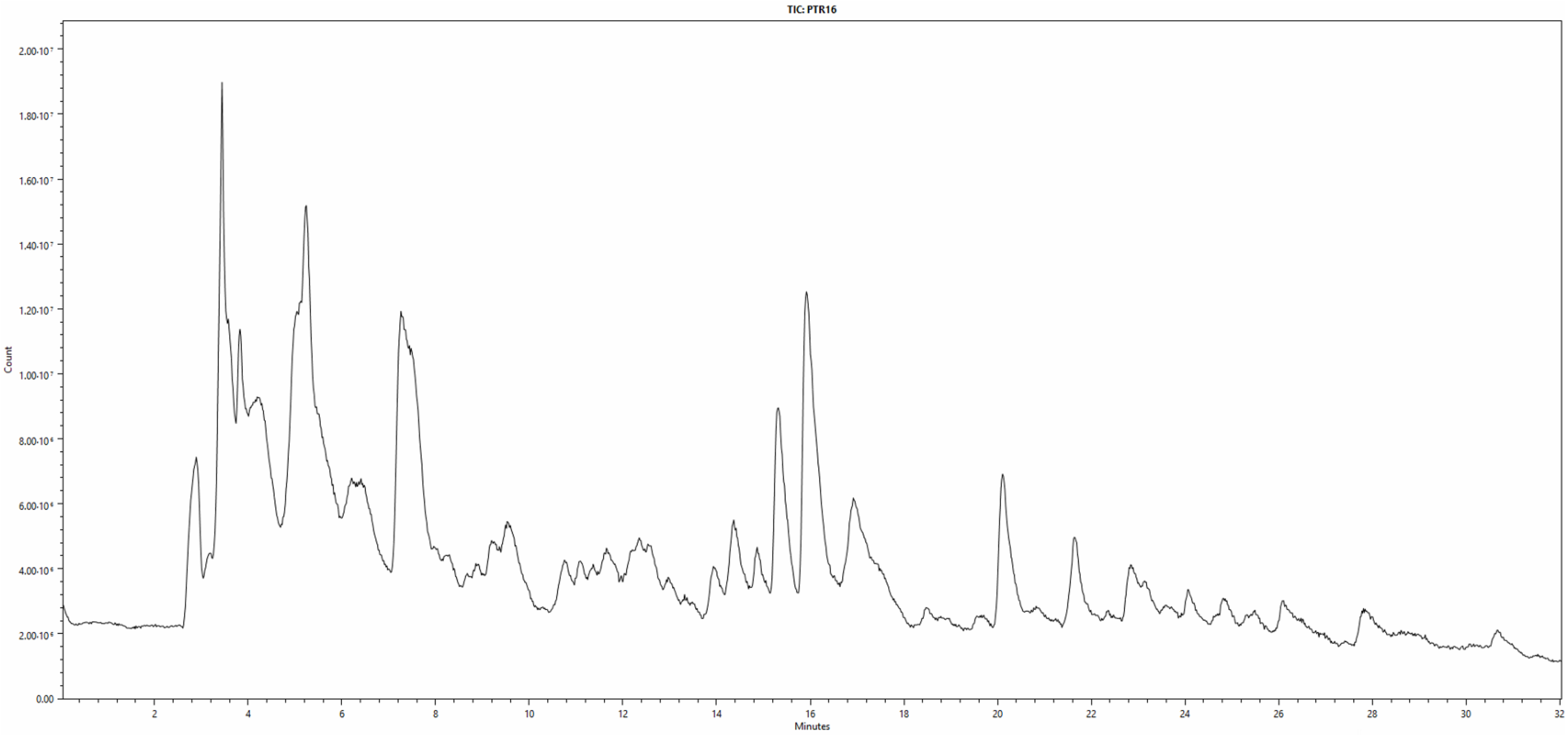

TIC: PTR16
Count
2.00·10⁷
1.80·10⁷
1.60·10⁷
1.40·10⁷
1.20·10⁷
1.00·10⁷
8.00·10⁶
6.00·10⁶
4.00·10⁶
2.00·10⁶
0.00
2 4 6 8 10 12 14 16 18 20 22 24 26 28 30 32
Minutes

BT 1/8 MS m

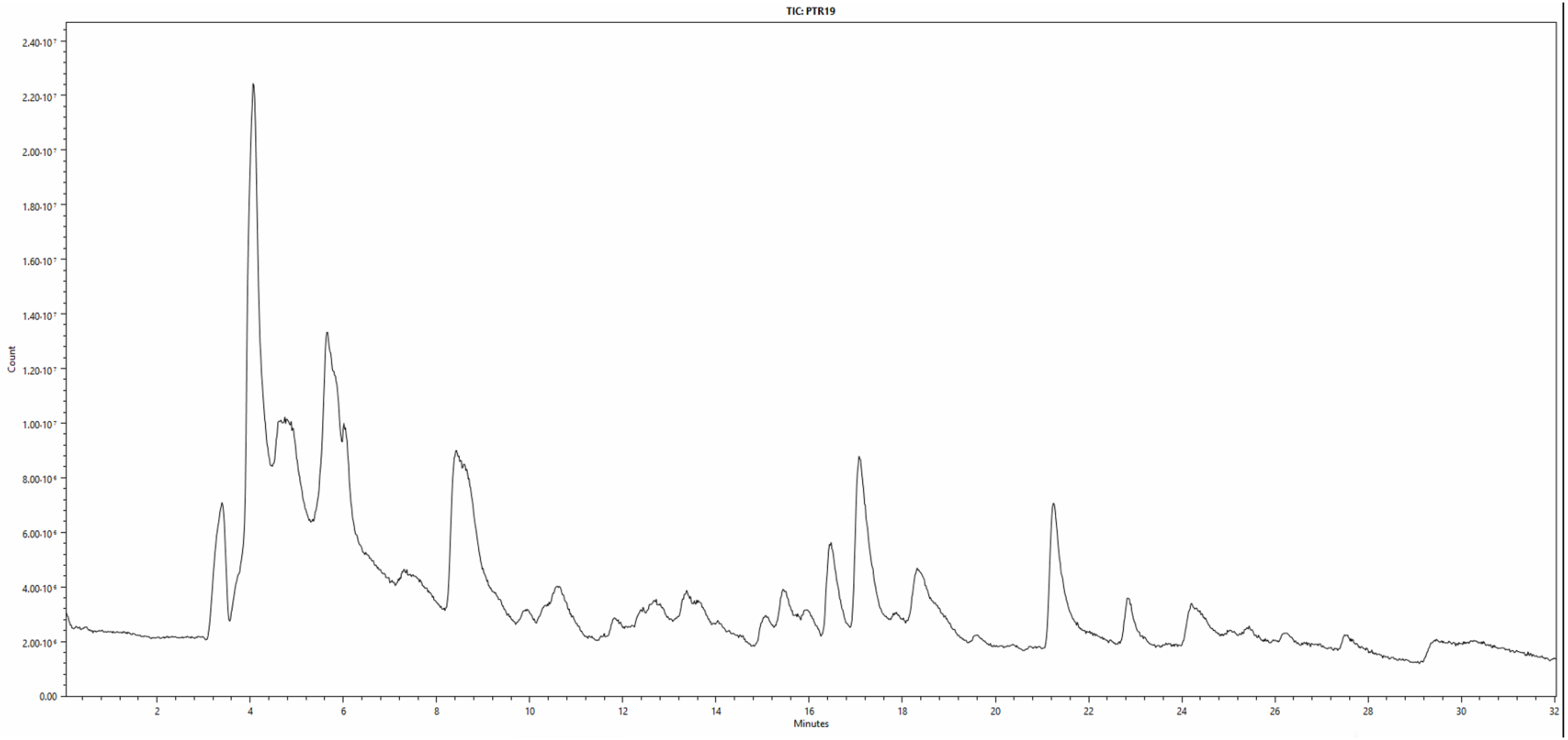

TIC: PTR19
Count
2.40·10⁷
2.20·10⁷
2.00·10⁷
1.80·10⁷
1.60·10⁷
1.40·10⁷
1.20·10⁷
1.00·10⁷
8.00·10⁶
6.00·10⁶
4.00·10⁶
2.00·10⁶
0.00
2
4
6
8
10
12
14
16
18
20
22
24
26
28
30
32
Minutes
