# Supplementary material for: The Combination of Untargeted Metabolomics and Machine Learning Predicts the Biosynthesis of Phenolic Compounds in Bryophyllum Medicinal Plants (Genus Kalanchoe)
Source: Plants (Basel). 2021 Nov 10;10(11):2430. doi: 10.3390/plants10112430 (PMC8620224; doi:10.3390/plants10112430)
Supplement: Supplementary file 1 [file plants-10-02430-s001.zip › total ion chromatograms.pdf]

Genotypes. BD: *B. daigremontianum*, BH: *B.* × *houghtonii*, BT: *B. tubiflorum*.

MS: Murashige and Skoog (1962) medium formulation; M: Macronutrients; m: micronutrients

BH Control

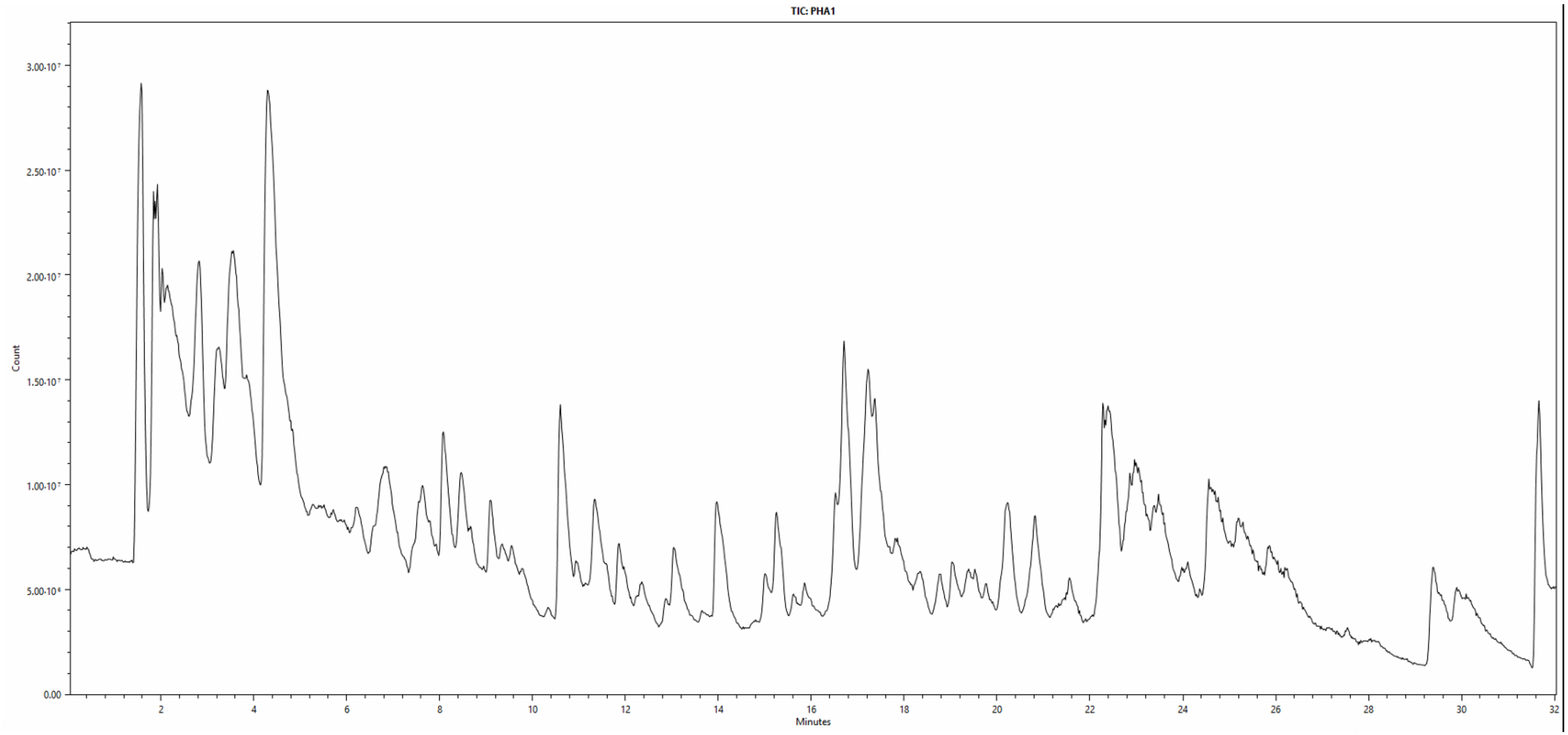

BH 1/2 MS M

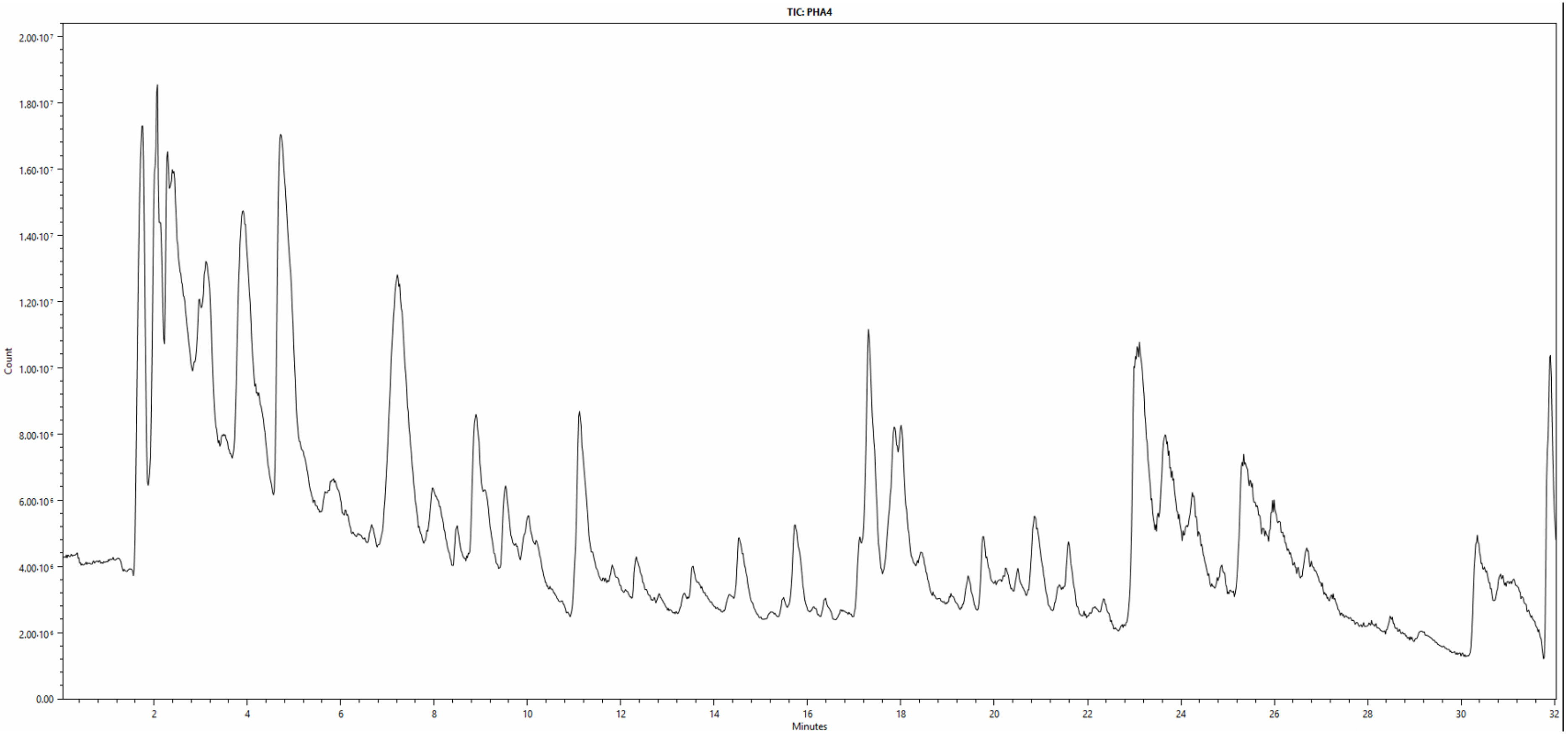
TIC: PHA4
Count
2.00·10⁷
1.80·10⁷
1.60·10⁷
1.40·10⁷
1.20·10⁷
1.00·10⁷
8.00·10⁶
6.00·10⁶
4.00·10⁶
2.00·10⁶
0.00
2
4
6
8
10
12
14
16
18
20
22
24
26
28
30
32
Minutes

BH 1/4 MS M

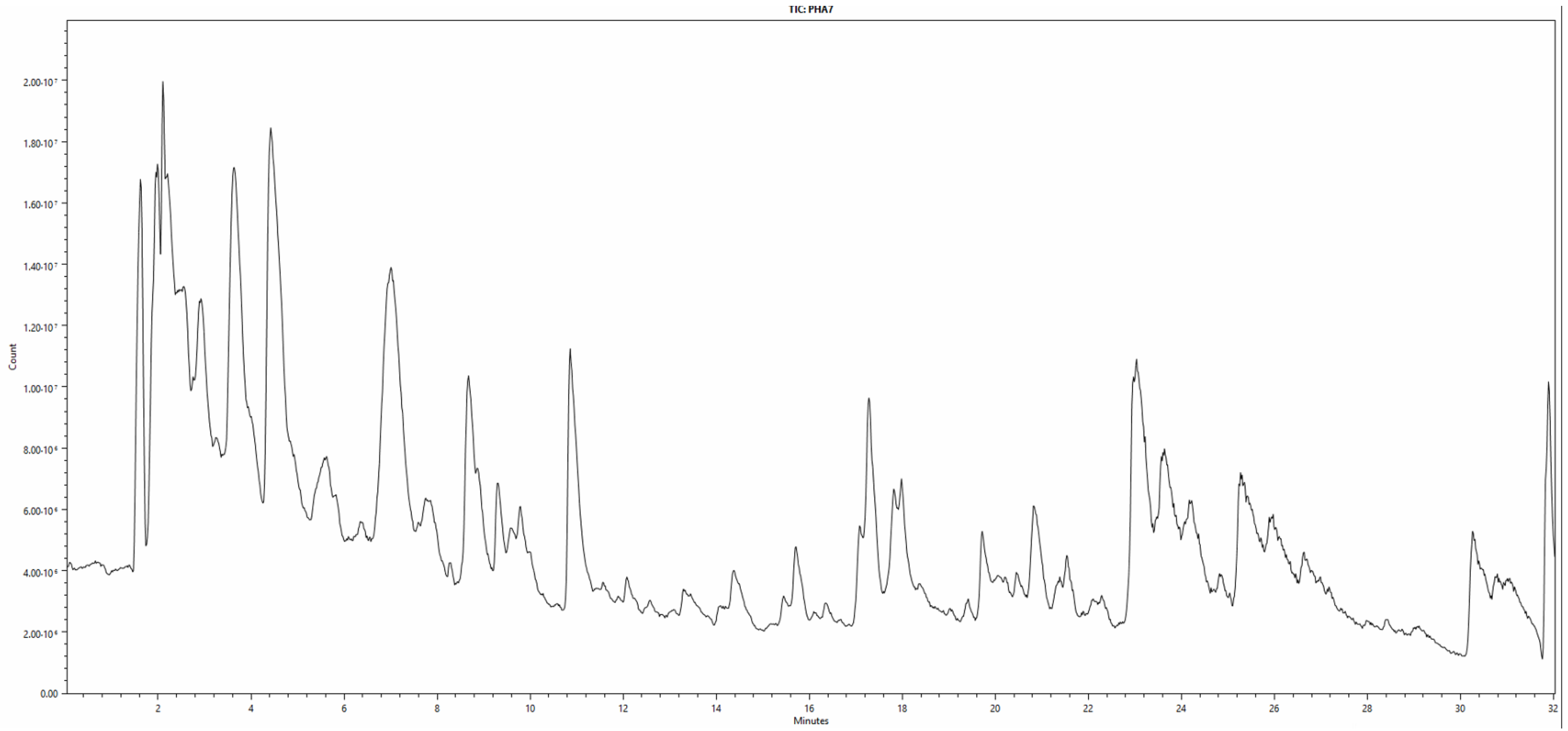
TIC: PHA7
Count
2.00·10⁷
1.80·10⁷
1.60·10⁷
1.40·10⁷
1.20·10⁷
1.00·10⁷
8.00·10⁶
6.00·10⁶
4.00·10⁶
2.00·10⁶
0.00
2
4
6
8
10
12
14
16
18
20
22
24
26
28
30
32
Minutes

BH 1/8 MS M

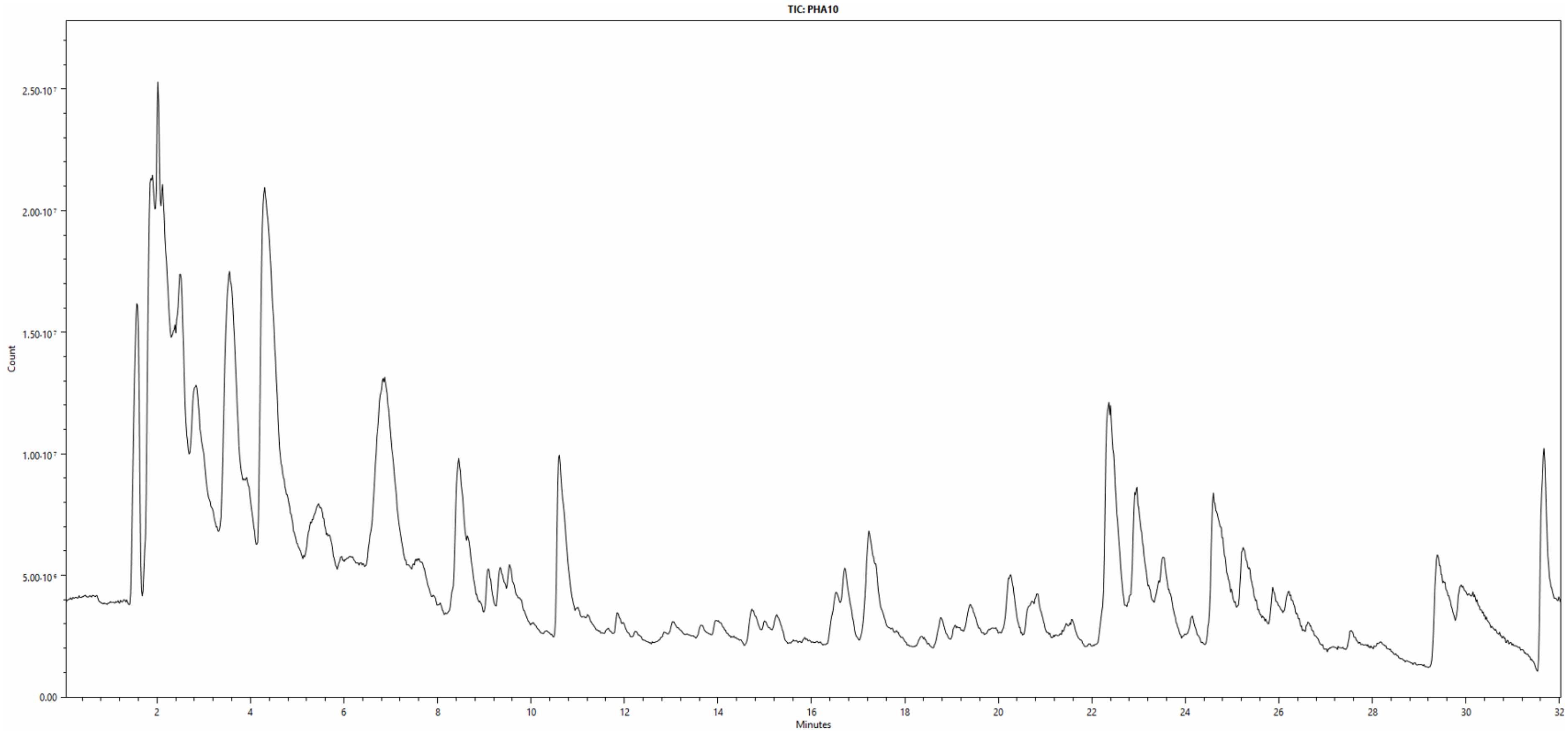
TIC: PHA10
Count
2,50·10⁷
2,00·10⁷
1,50·10⁷
1,00·10⁷
5,00·10⁶
0,00
2
4
6
8
10
12
14
16
18
20
22
24
26
28
30
32
Minutes

BH 1/2 MS M

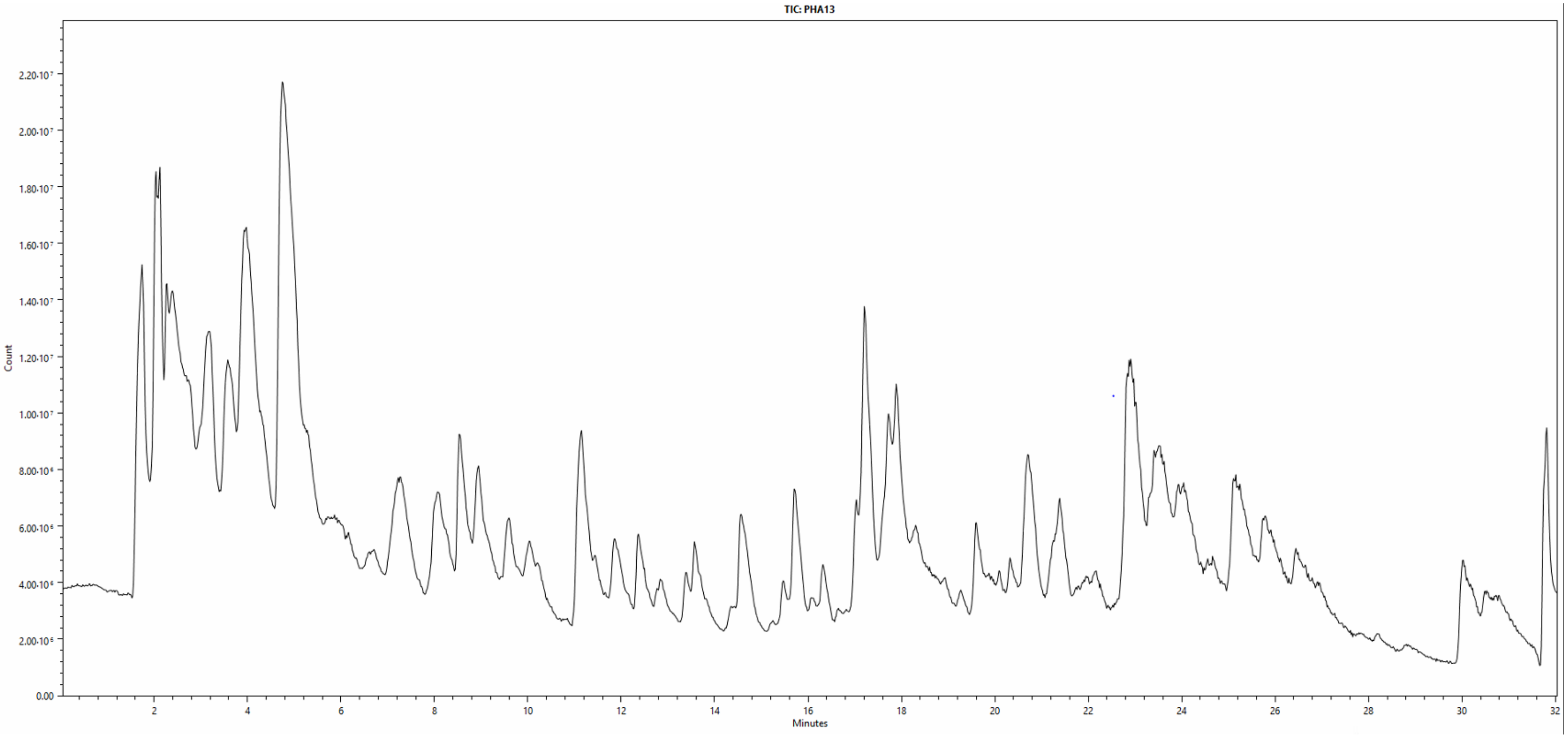
TIC: PHA13
Count
2.20·10⁷
2.00·10⁷
1.80·10⁷
1.60·10⁷
1.40·10⁷
1.20·10⁷
1.00·10⁷
8.00·10⁶
6.00·10⁶
4.00·10⁶
2.00·10⁶
0.00
2 4 6 8 10 12 14 16 18 20 22 24 26 28 30 32
Minutes

BH 1/4 MS m

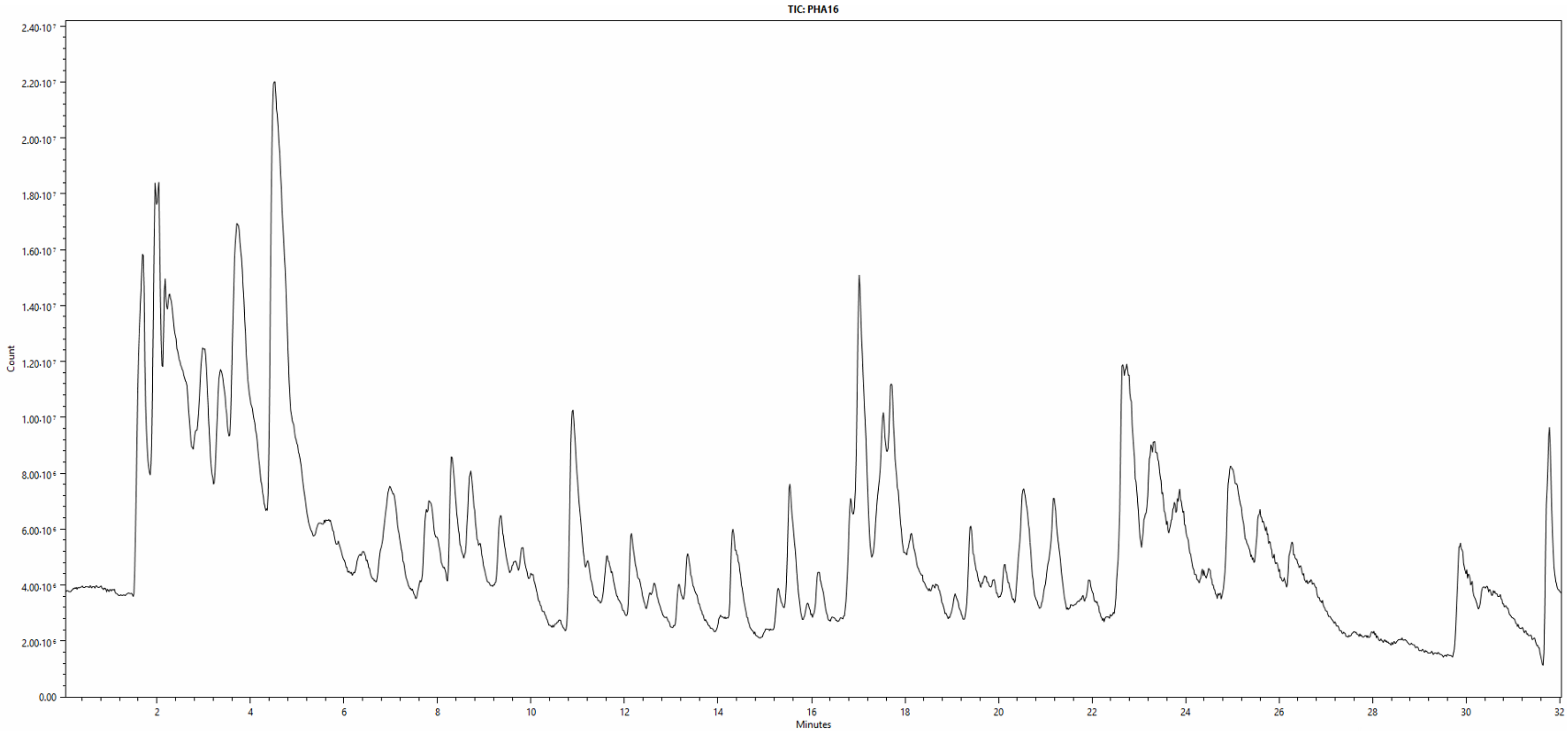

TIC: PHA16
Count
2.40·10⁷
2.20·10⁷
2.00·10⁷
1.80·10⁷
1.60·10⁷
1.40·10⁷
1.20·10⁷
1.00·10⁷
8.00·10⁶
6.00·10⁶
4.00·10⁶
2.00·10⁶
0.00
2 4 6 8 10 12 14 16 18 20 22 24 26 28 30 32
Minutes

BH 1/8 MS m

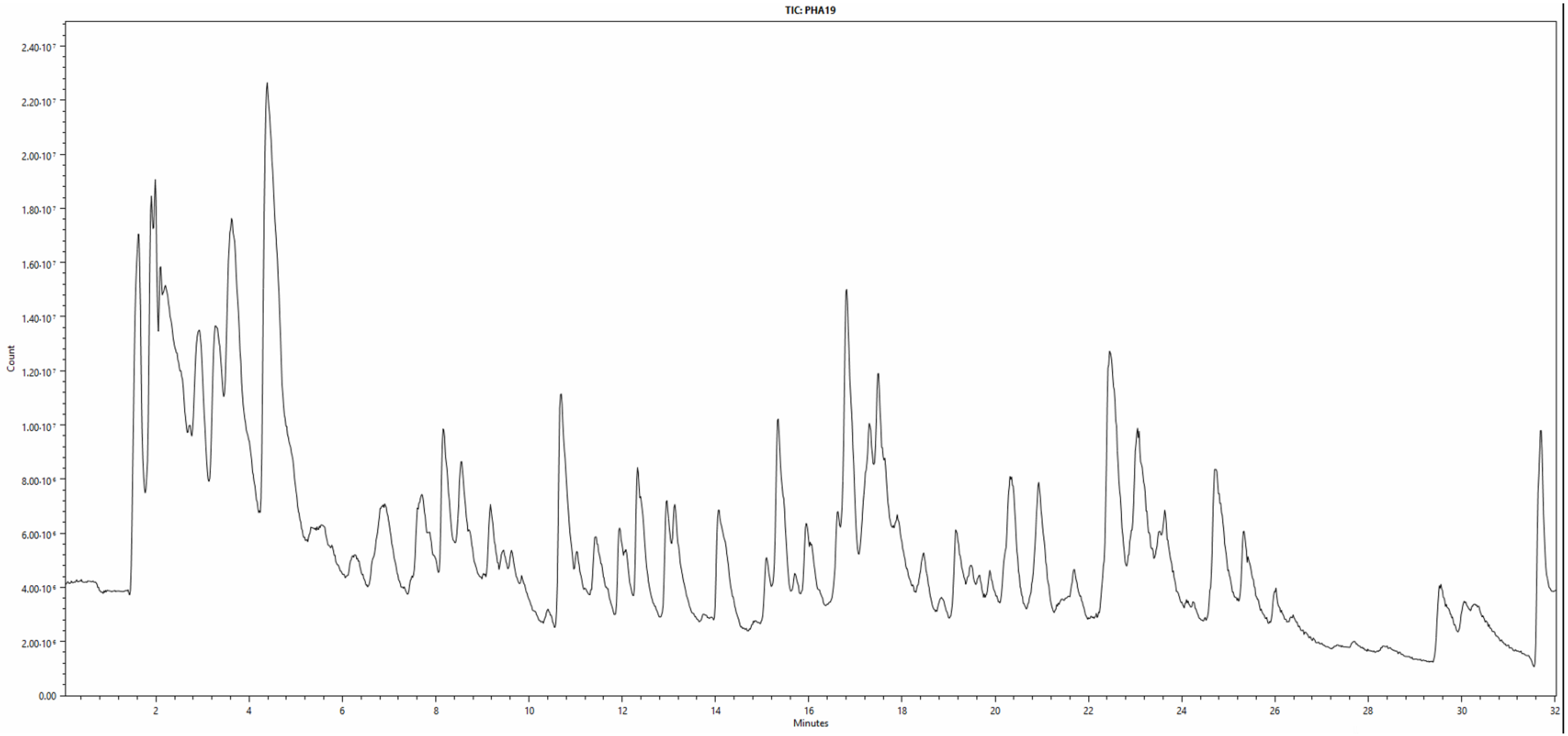

TIC: PHA19
Count
2.40·10⁷
2.20·10⁷
2.00·10⁷
1.80·10⁷
1.60·10⁷
1.40·10⁷
1.20·10⁷
1.00·10⁷
8.00·10⁶
6.00·10⁶
4.00·10⁶
2.00·10⁶
0.00
2
4
6
8
10
12
14
16
18
20
22
24
26
28
30
32
Minutes

BD Control

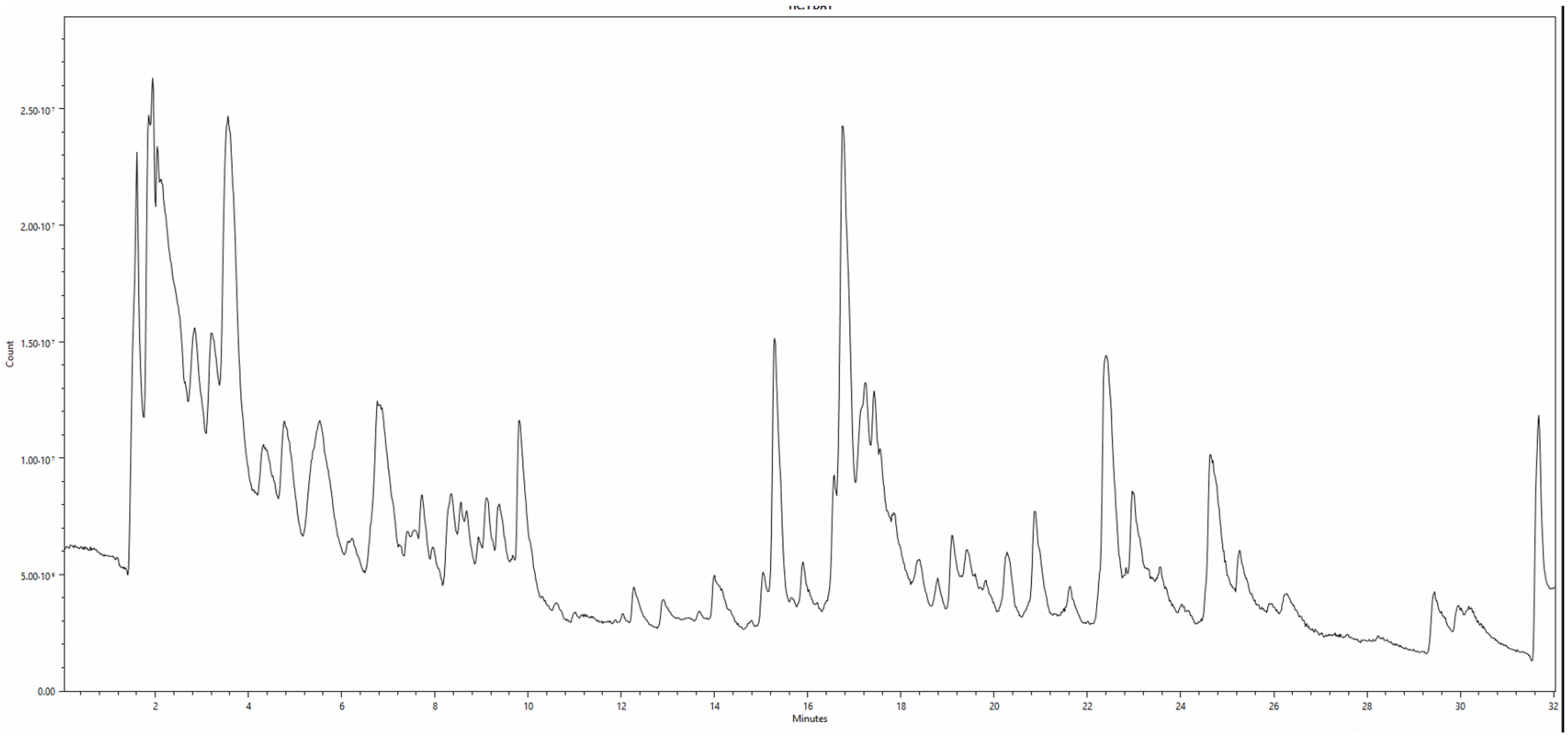

Count
2.50·10⁷
2.00·10⁷
1.50·10⁷
1.00·10⁷
5.00·10⁶
0.00
2
4
6
8
10
12
14
16
18
20
22
24
26
28
30
32
Minutes

BD 1/2 MS M

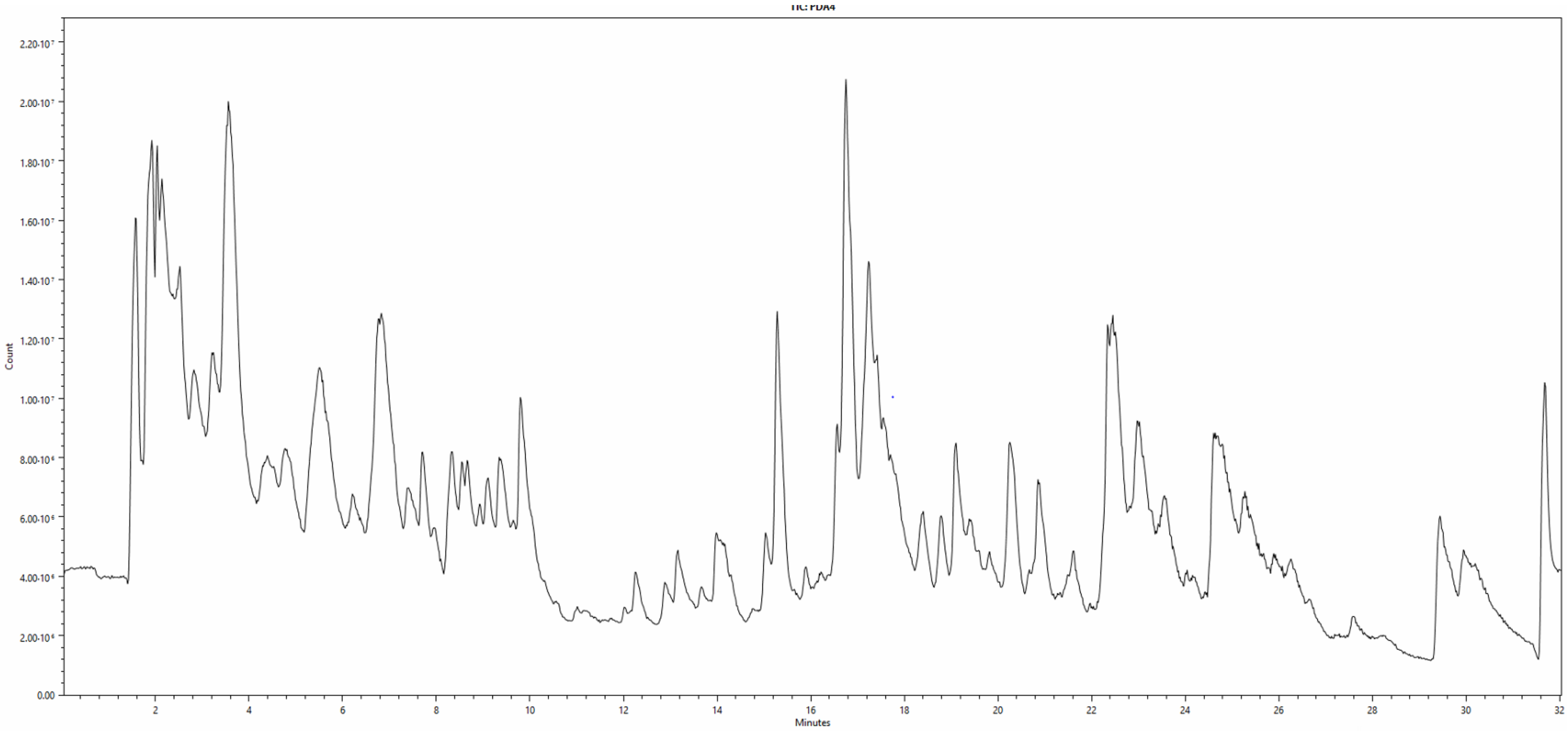

Count
2.20·10⁷
2.00·10⁷
1.80·10⁷
1.60·10⁷
1.40·10⁷
1.20·10⁷
1.00·10⁷
8.00·10⁶
6.00·10⁶
4.00·10⁶
2.00·10⁶
0.00
2
4
6
8
10
12
14
16
18
20
22
24
26
28
30
32
Minutes

BD 1/4 MS M

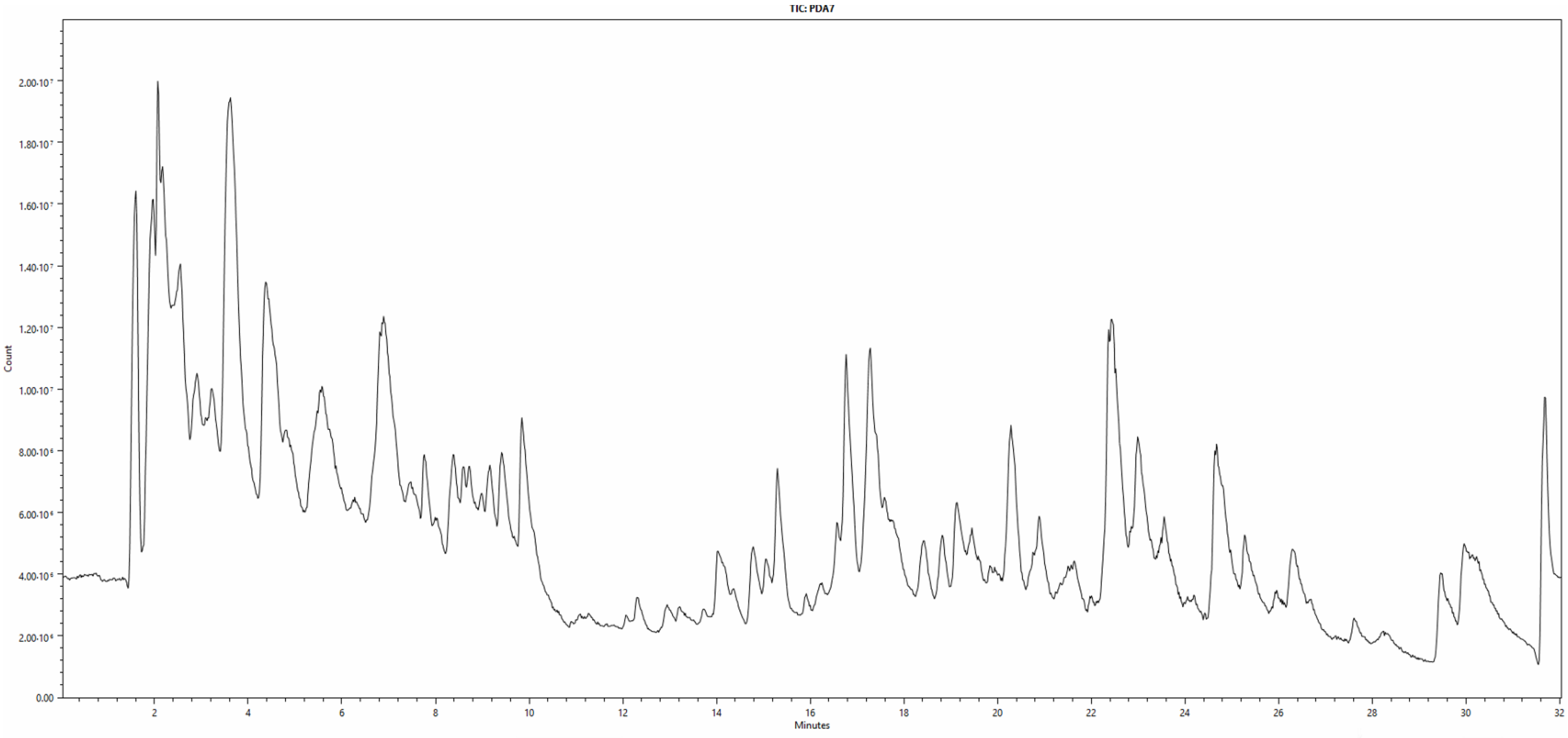
TIC: PDA7
Count
2.00·10⁷
1.80·10⁷
1.60·10⁷
1.40·10⁷
1.20·10⁷
1.00·10⁷
8.00·10⁶
6.00·10⁶
4.00·10⁶
2.00·10⁶
0.00
2
4
6
8
10
12
14
16
18
20
22
24
26
28
30
32
Minutes

BD 1/8 MS M

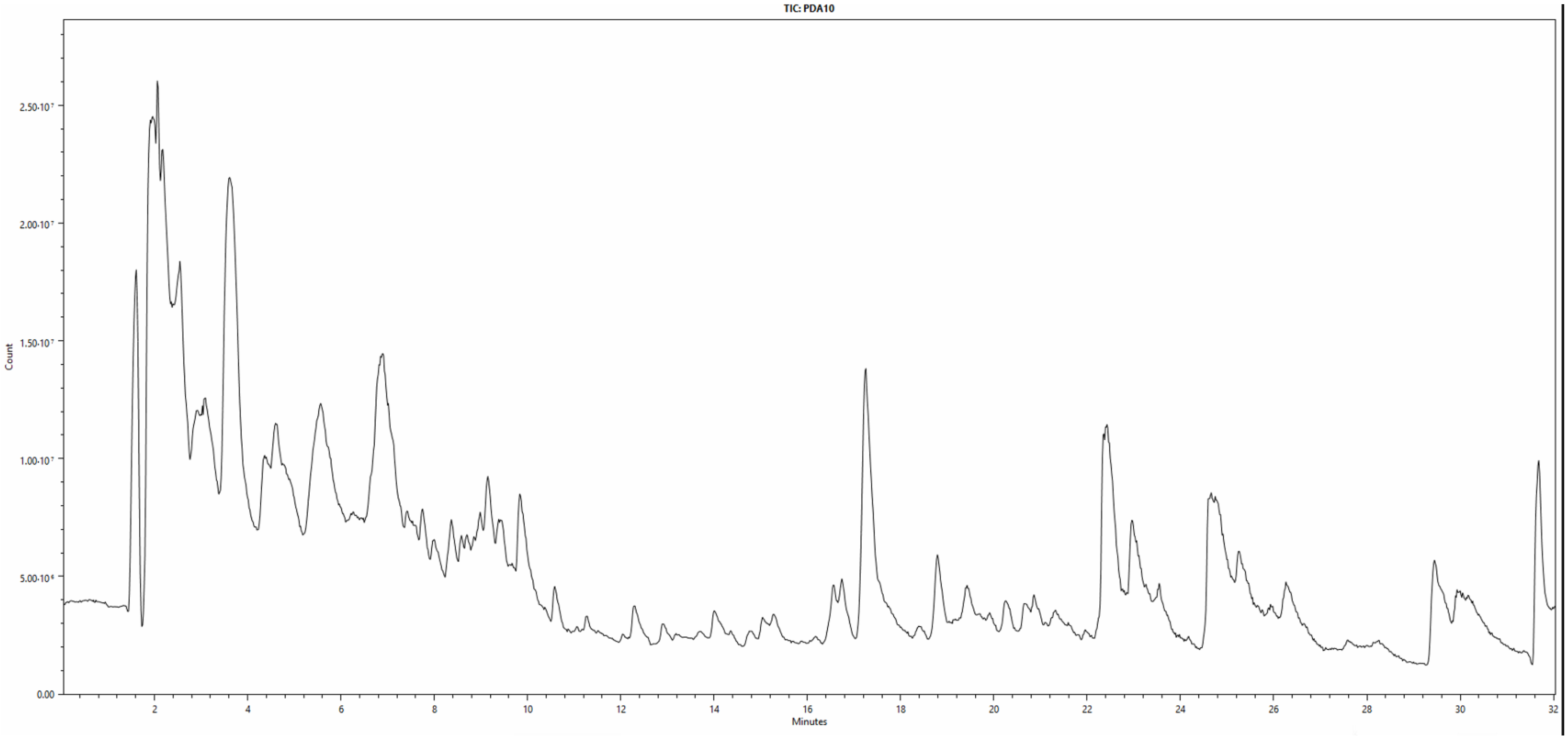

TIC: PDA10
Count
2.50·10⁷
2.00·10⁷
1.50·10⁷
1.00·10⁷
5.00·10⁶
0.00
2
4
6
8
10
12
14
16
18
20
22
24
26
28
30
32
Minutes

BD 1/2 MS m

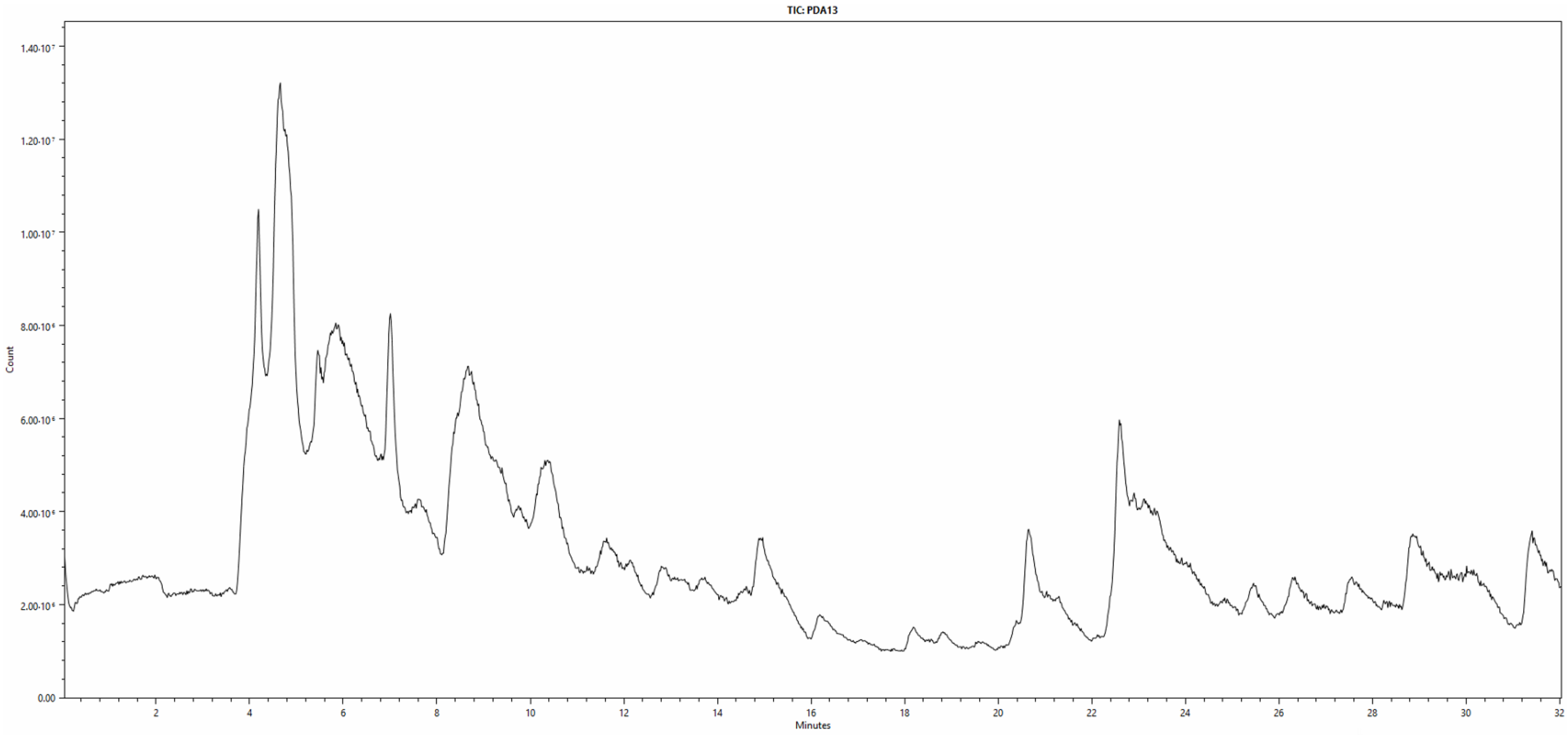

TIC: PDA13
Count
Minutes
1.40·10⁷
1.20·10⁷
1.00·10⁷
8.00·10⁶
6.00·10⁶
4.00·10⁶
2.00·10⁶
0.00
2
4
6
8
10
12
14
16
18
20
22
24
26
28
30
32

BD 1/4 MS m

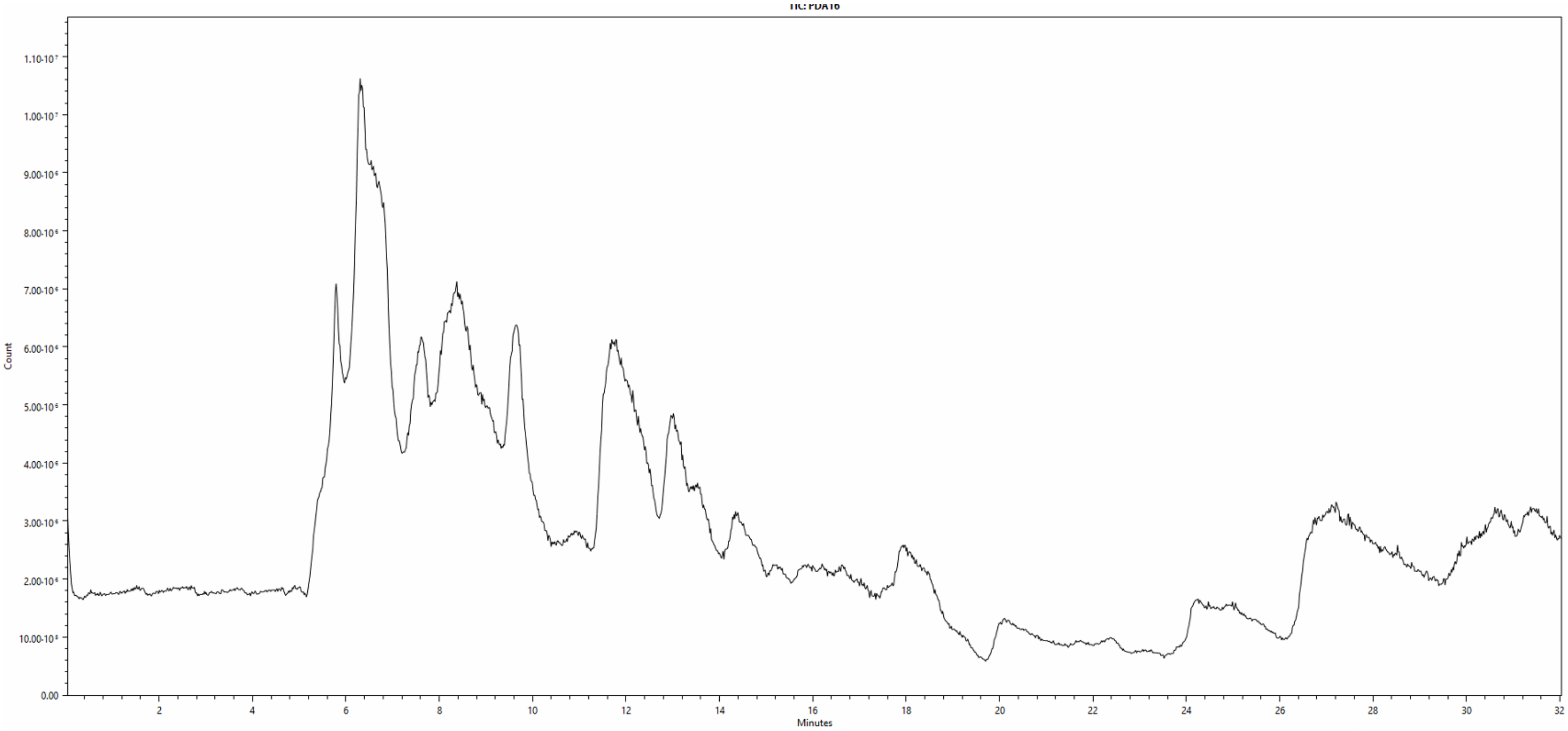

TIC: PDA16
1.10·10⁷
1.00·10⁷
9.00·10⁶
8.00·10⁶
7.00·10⁶
6.00·10⁶
5.00·10⁶
4.00·10⁶
3.00·10⁶
2.00·10⁶
10.00·10⁵
0.00
Count
2
4
6
8
10
12
14
16
18
20
22
24
26
28
30
32
Minutes

BD 1/8 MS m

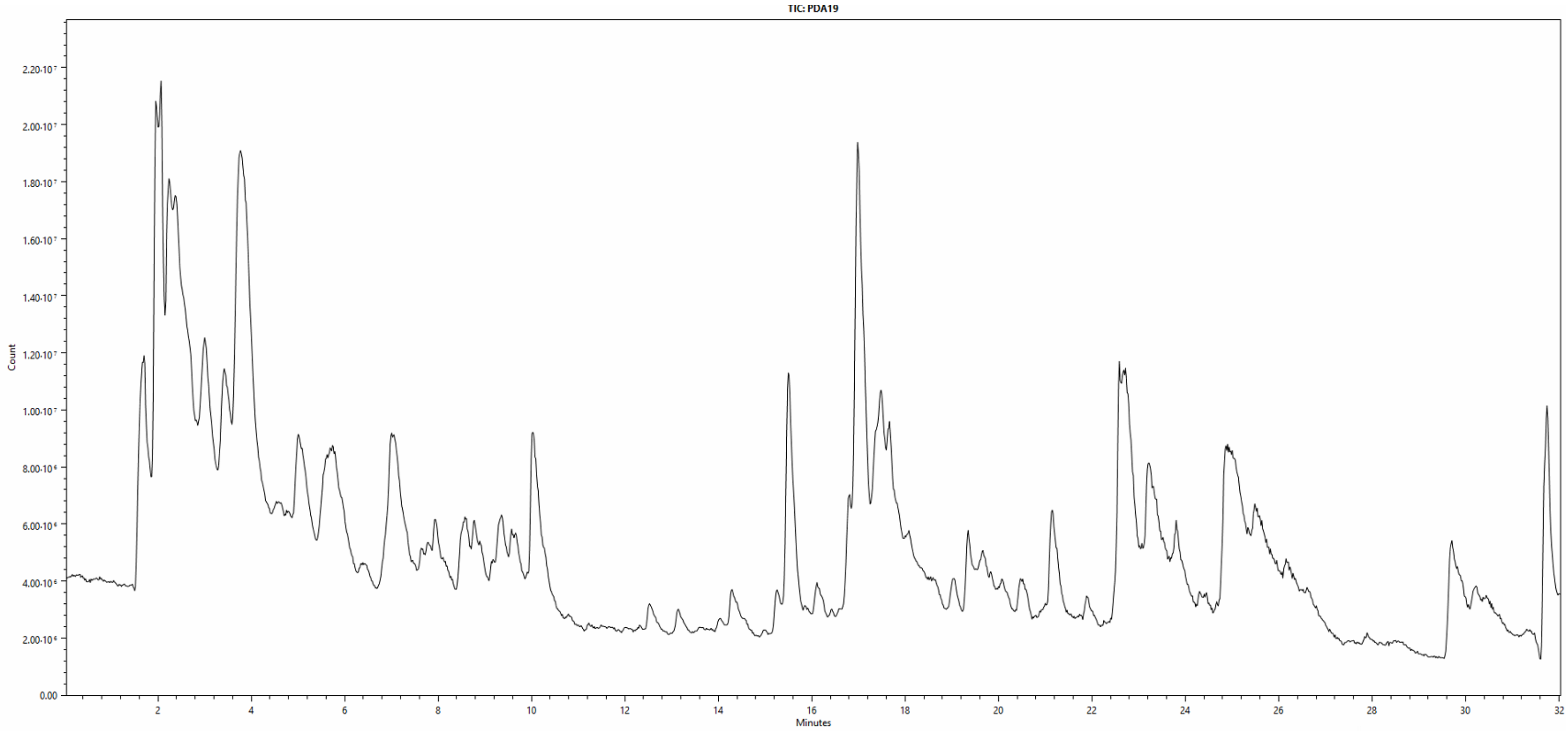

TIC: PDA19
Count
2.20·10⁷
2.00·10⁷
1.80·10⁷
1.60·10⁷
1.40·10⁷
1.20·10⁷
1.00·10⁷
8.00·10⁶
6.00·10⁶
4.00·10⁶
2.00·10⁶
0.00
2 4 6 8 10 12 14 16 18 20 22 24 26 28 30 32
Minutes

BT Control

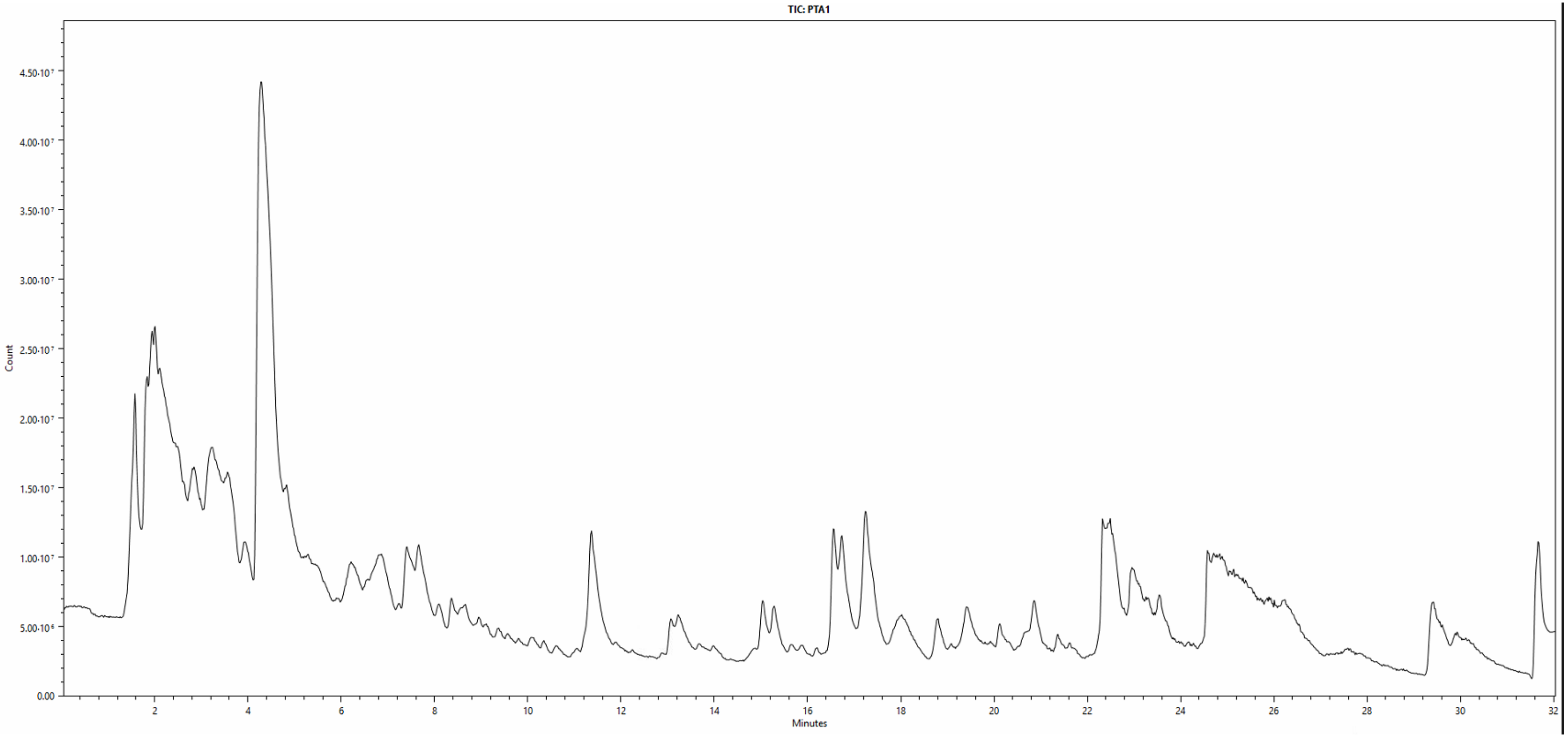

TIC: PTA1
Count
4.50·10⁷
4.00·10⁷
3.50·10⁷
3.00·10⁷
2.50·10⁷
2.00·10⁷
1.50·10⁷
1.00·10⁷
5.00·10⁶
0.00
2
4
6
8
10
12
14
16
18
20
22
24
26
28
30
32
Minutes

BT 1/2 MS M

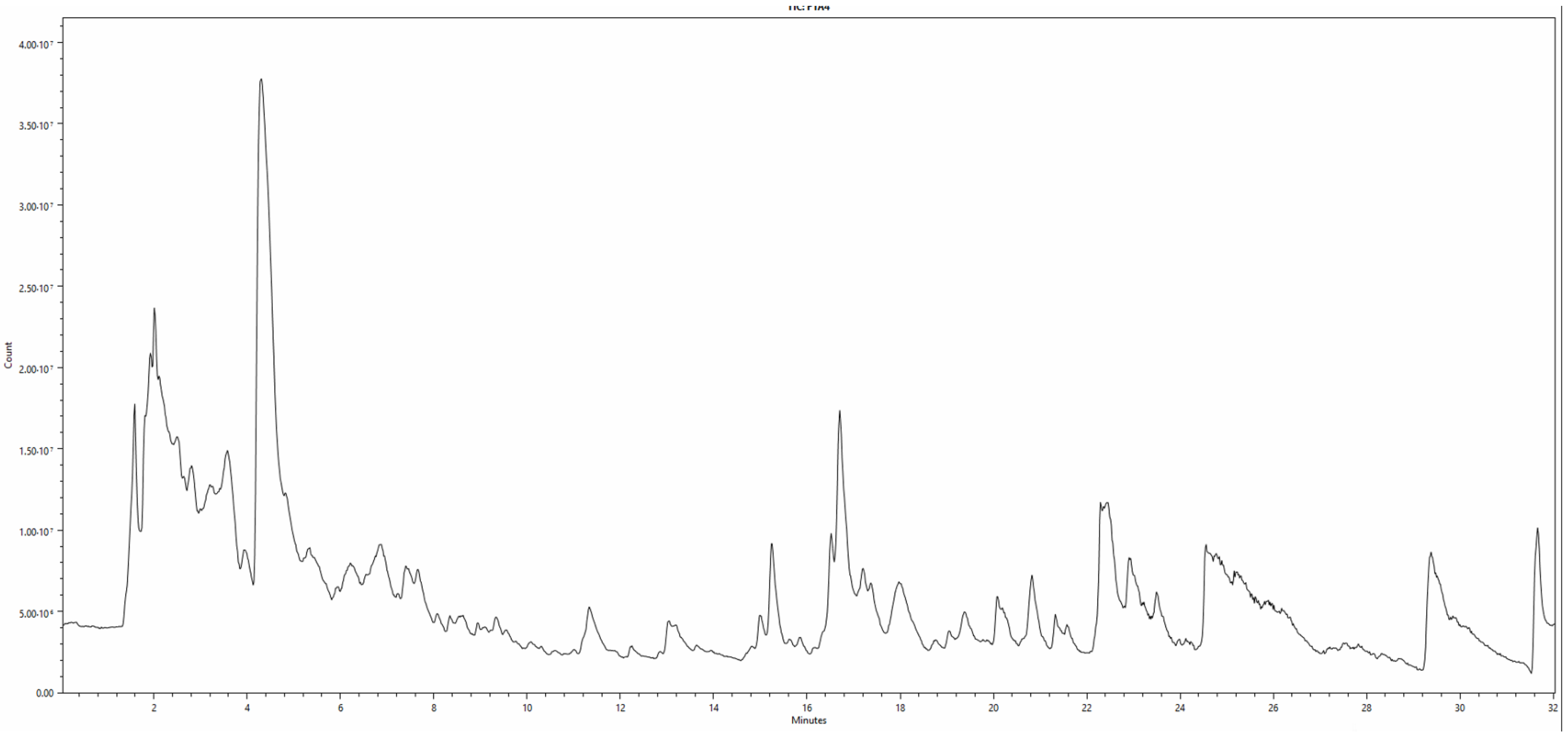

Count
4.00·10⁷
3.50·10⁷
3.00·10⁷
2.50·10⁷
2.00·10⁷
1.50·10⁷
1.00·10⁷
5.00·10⁶
0.00
2
4
6
8
10
12
14
16
18
20
22
24
26
28
30
32
Minutes

BT 1/4 MS M

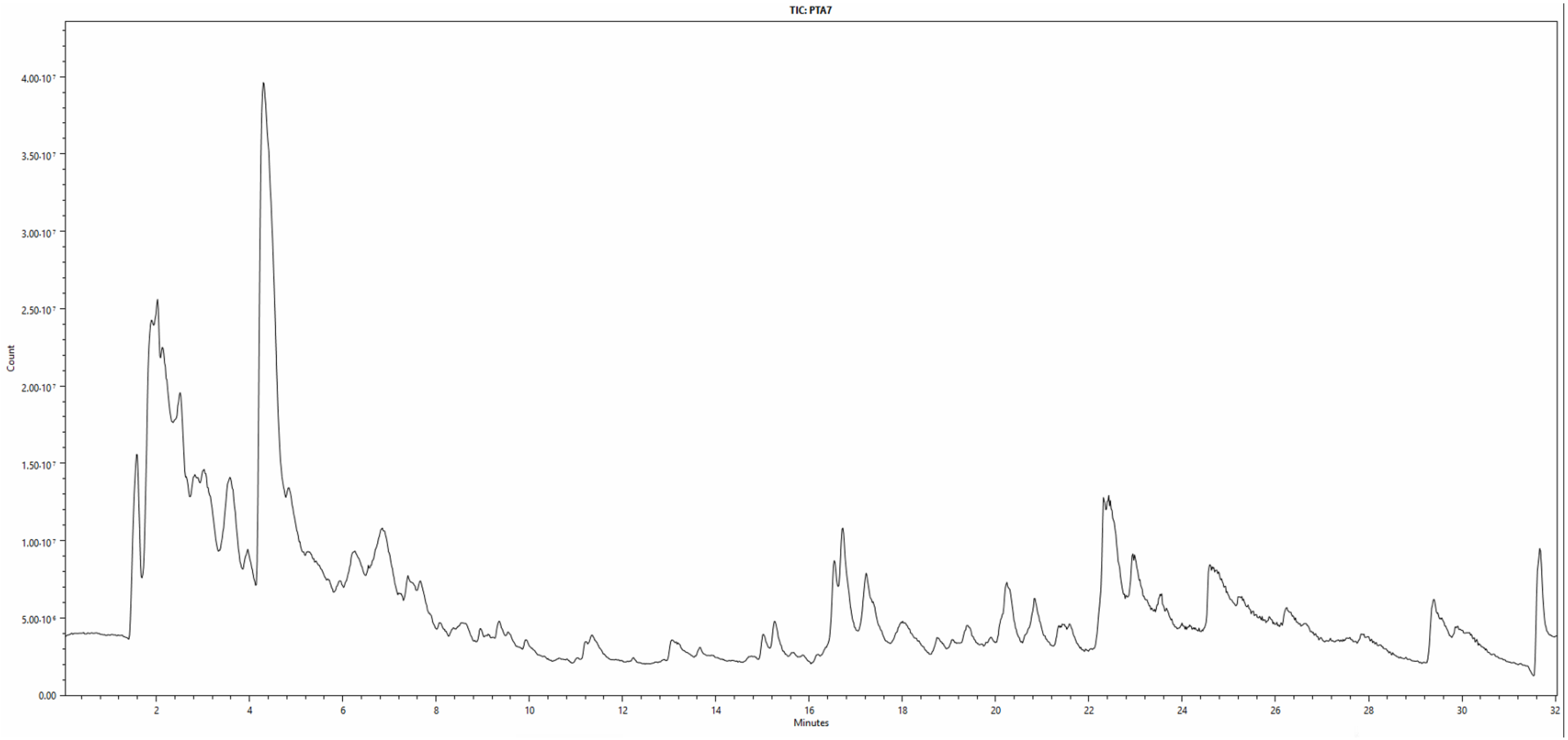

TIC: PTA7
Count
4.00·10⁷
3.50·10⁷
3.00·10⁷
2.50·10⁷
2.00·10⁷
1.50·10⁷
1.00·10⁷
5.00·10⁶
0.00
2
4
6
8
10
12
14
16
18
20
22
24
26
28
30
32
Minutes

BT 1/8 MS M

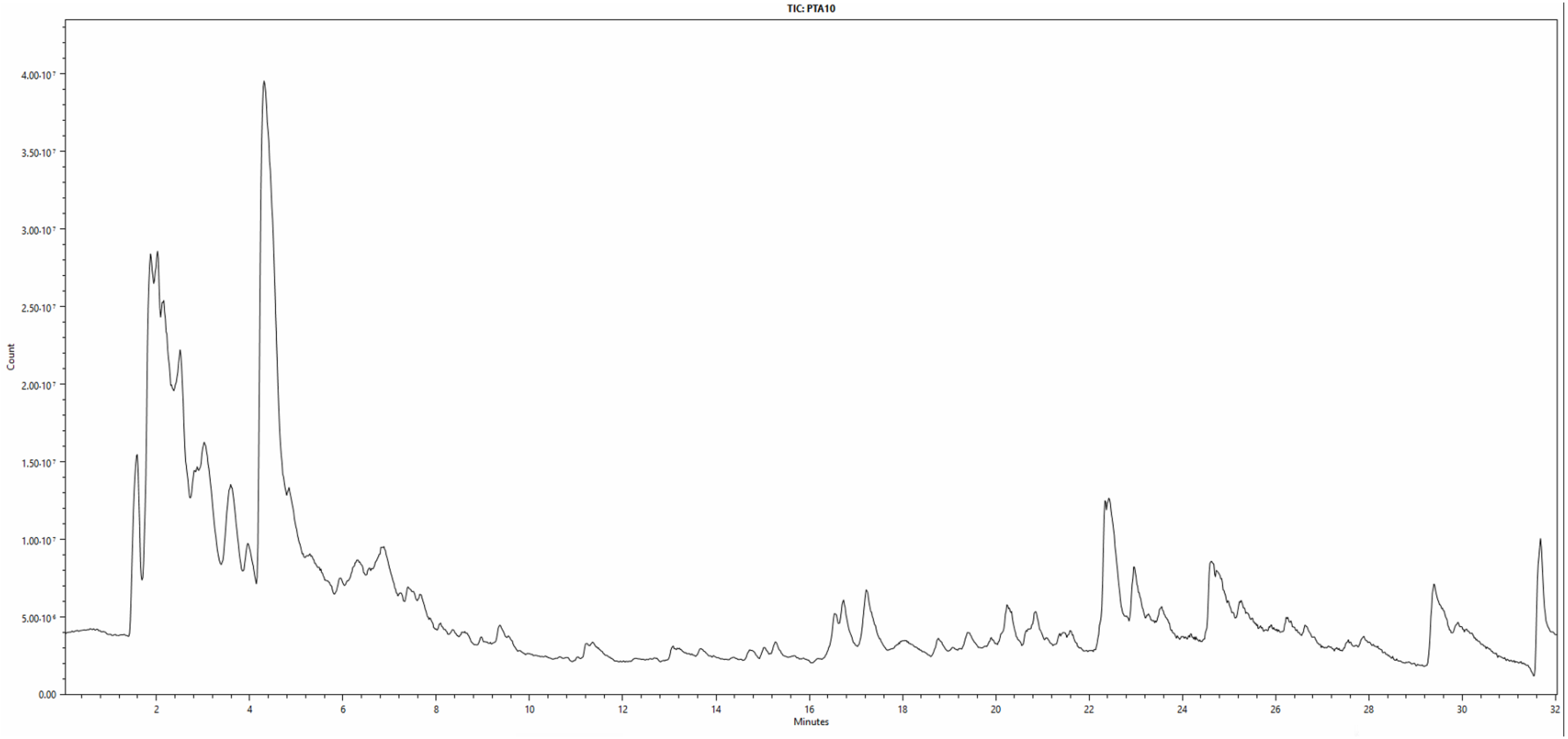
TIC: PTA10
Count
4.00·10⁷
3.50·10⁷
3.00·10⁷
2.50·10⁷
2.00·10⁷
1.50·10⁷
1.00·10⁷
5.00·10⁶
0.00
2
4
6
8
10
12
14
16
18
20
22
24
26
28
30
32
Minutes

BT 1/2 MS m

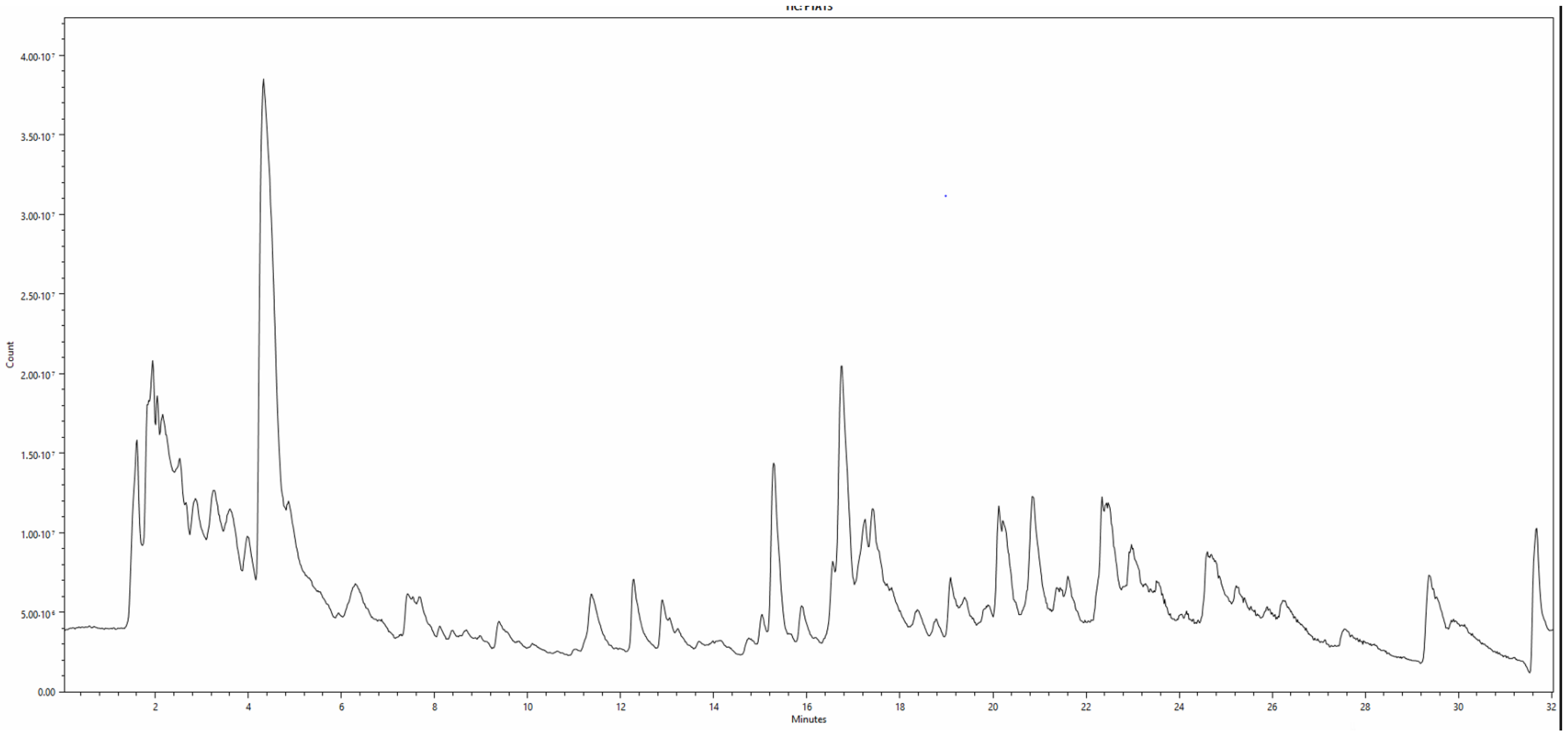

Count
4.00·10⁷
3.50·10⁷
3.00·10⁷
2.50·10⁷
2.00·10⁷
1.50·10⁷
1.00·10⁷
5.00·10⁶
0.00
2
4
6
8
10
12
14
16
18
20
22
24
26
28
30
32
Minutes

BT 1/4 MS m

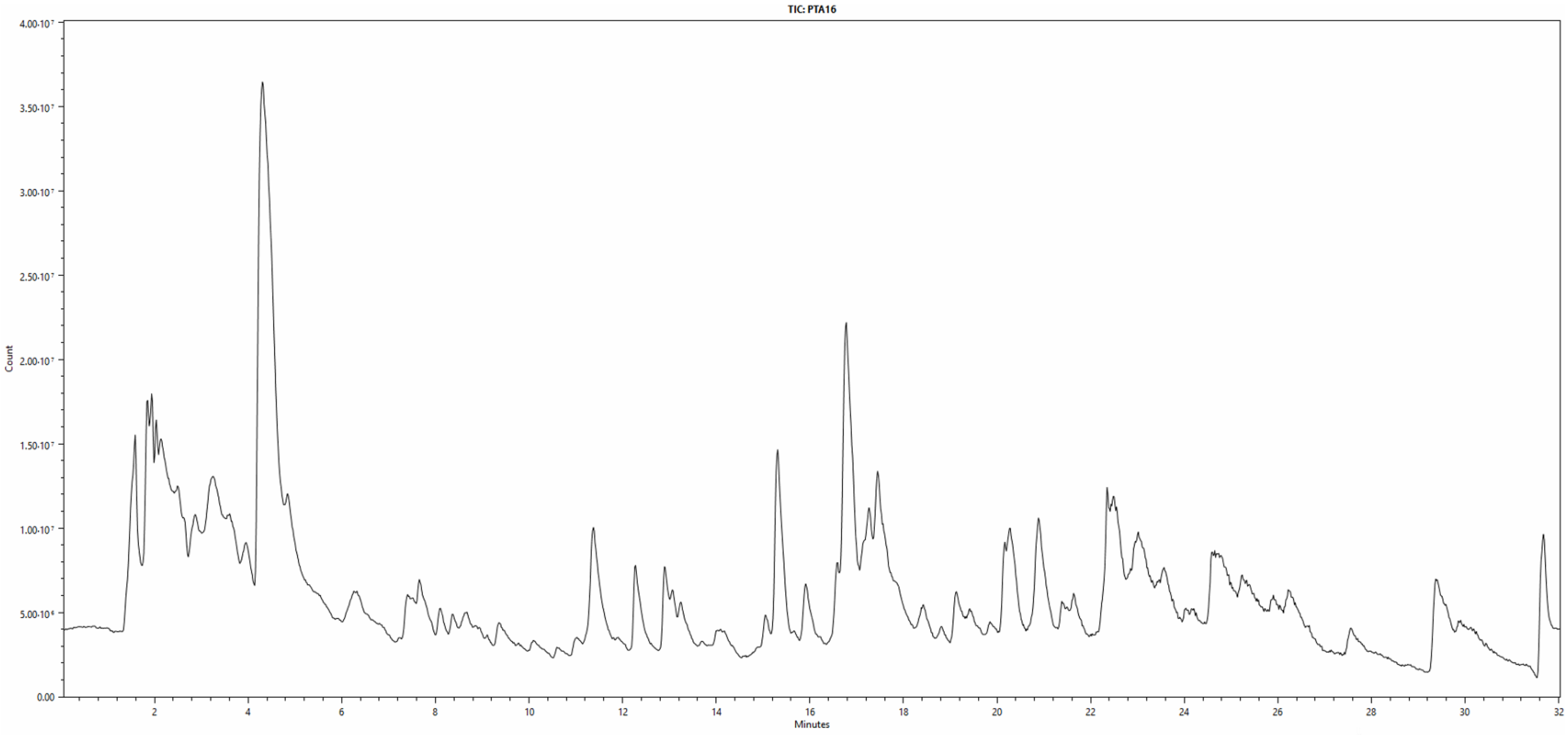

TIC: PTA16
Count
Minutes
4.00·10⁷
3.50·10⁷
3.00·10⁷
2.50·10⁷
2.00·10⁷
1.50·10⁷
1.00·10⁷
5.00·10⁶
0.00
2
4
6
8
10
12
14
16
18
20
22
24
26
28
30
32

BT 1/8 MS m

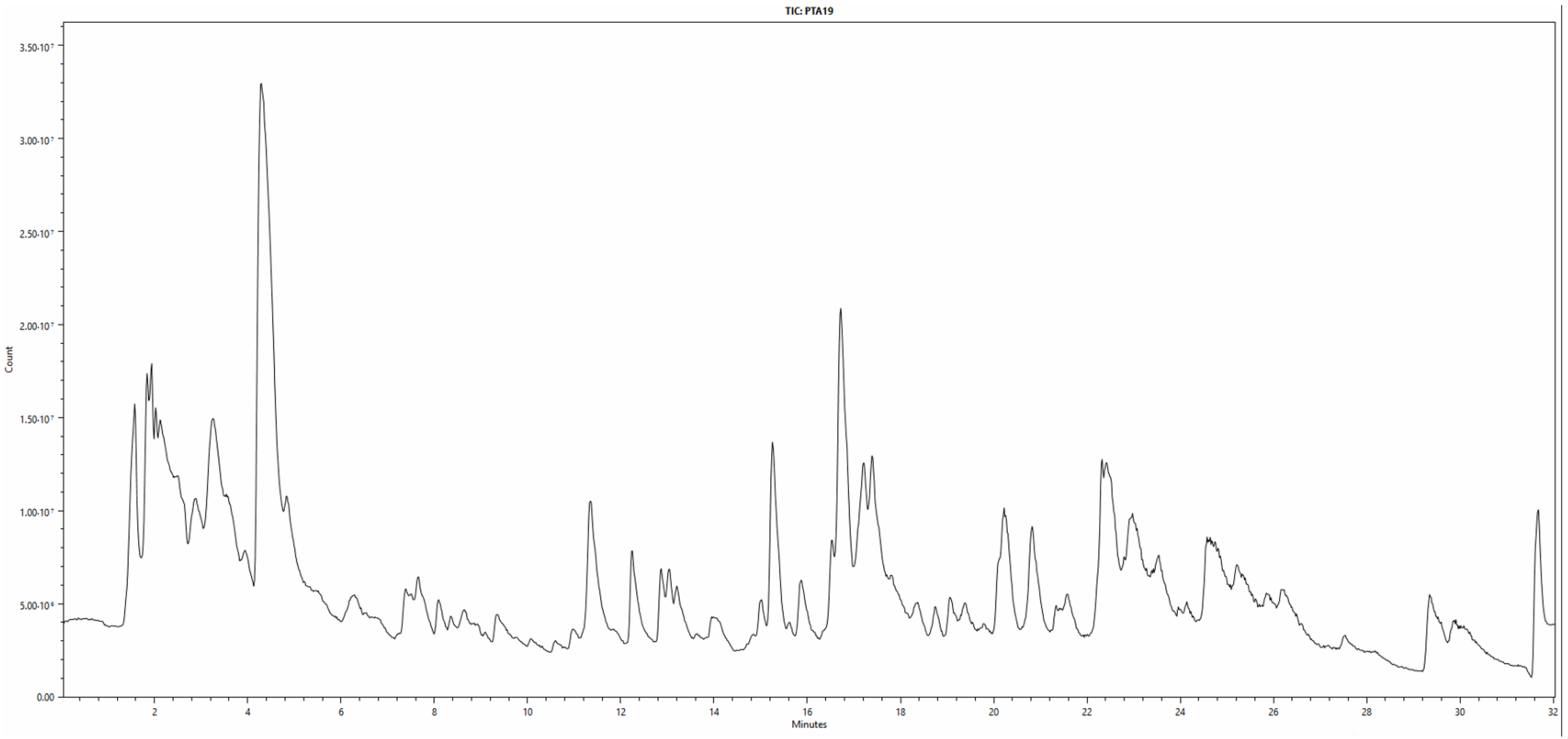

TIC: PTA19
3.50·10⁷
3.00·10⁷
2.50·10⁷
2.00·10⁷
1.50·10⁷
1.00·10⁷
5.00·10⁶
0.00
Count
2
4
6
8
10
12
14
16
18
20
22
24
26
28
30
32
Minutes
